# Life cycle synchronization is a viral drug resistance mechanism

**DOI:** 10.1371/journal.pcbi.1005947

**Published:** 2018-02-15

**Authors:** Iulia A. Neagu, Jason Olejarz, Mark Freeman, Daniel I.S. Rosenbloom, Martin A. Nowak, Alison L. Hill

**Affiliations:** 1 Program for Evolutionary Dynamics, Department of Mathematics and Department of Organismic & Evolutionary Biology, Harvard University, Cambridge, Massachusetts, United States of America; 2 Department of Physics, Harvard University, Cambridge, Massachusetts, United States of America; 3 Department of Biomedical Informatics, Columbia University Medical Center, New York, New York, United States of America; University of California Irvine, UNITED STATES

## Abstract

Viral infections are one of the major causes of death worldwide, with HIV infection alone resulting in over 1.2 million casualties per year. Antiviral drugs are now being administered for a variety of viral infections, including HIV, hepatitis B and C, and influenza. These therapies target a specific phase of the virus’s life cycle, yet their ultimate success depends on a variety of factors, such as adherence to a prescribed regimen and the emergence of viral drug resistance. The epidemiology and evolution of drug resistance have been extensively characterized, and it is generally assumed that drug resistance arises from mutations that alter the virus’s susceptibility to the direct action of the drug. In this paper, we consider the possibility that a virus population can evolve towards synchronizing its life cycle with the pattern of drug therapy. The periodicity of the drug treatment could then allow for a virus strain whose life cycle length is a multiple of the dosing interval to replicate only when the concentration of the drug is lowest. This process, referred to as “drug tolerance by synchronization”, could allow the virus population to maximize its overall fitness without having to alter drug binding or complete its life cycle in the drug’s presence. We use mathematical models and stochastic simulations to show that life cycle synchronization can indeed be a mechanism of viral drug tolerance. We show that this effect is more likely to occur when the variability in both viral life cycle and drug dose timing are low. More generally, we find that in the presence of periodic drug levels, time-averaged calculations of viral fitness do not accurately predict drug levels needed to eradicate infection, even if there is no synchronization. We derive an analytical expression for viral fitness that is sufficient to explain the drug-pattern-dependent survival of strains with any life cycle length. We discuss the implications of these findings for clinically relevant antiviral strategies.

## Author summary

Viral infections such as HIV, hepatitis B, hepatitis C, and influenza may be treated with antiviral drug therapy. These drugs generally block a specific phase of the virus’s life cycle, preventing it from replicating in the body. Often, a virus population can evolve drug resistance by acquiring mutations that prevent the drug from binding and inhibiting it. Here, we propose a new mechanism of drug resistance. We use mathematical models and stochastic simulations to show that virus populations can build resistance to antiviral therapies by synchronizing their life cycle with the dosing pattern of the drug. This process, which we refer to as “drug tolerance by synchronization”, can allow the virus to increase its overall fitness in the presence of the drug without altering drug binding. We show that viral strains whose life cycle lengths are approximately integer multiples of the time between drug doses possess an advantage during drug therapy. They can outcompete unsynchronized strains and lead to therapy failure with drugs that would otherwise have been successful.

## Introduction

Viral infections are a major cause of human morbidity and mortality [1]. While vaccines to prevent viral illnesses have existed for over a century, it is only in the past several decades that drugs directly targeting viral replication have been developed. Antiviral drugs now exist for pathogens including the human immunodeficiency virus (HIV) [2], hepatitis B [3] and C [4] viruses, influenza A and B viruses [5], herpes simplex viruses [6], cytomegalovirus [7], Epstein Barr virus [8], and varicella zoster virus (chickenpox virus) [9]. These drugs each target a specific phase of the virus’s life cycle, and by binding to a viral protein or otherwise interfering with a critical step in viral replication are able to reduce the virus’s growth rate. Examples of viral functions targeted by antiviral drugs include binding of the viral particle to the target cell membrane, transcription of the viral genome, integration of the virus in the host cell genome, or post-translational cleavage of viral proteins.

Antiviral treatments that are initially successful at reducing viral loads may eventually be rendered ineffective, in individual patients or in entire populations, by the emergence of drug-resistant strains [7, 10–15]. Drug resistance occurs when a viral strain gains a mutation that allows it to replicate efficiently despite the presence of the drug, and this strain subsequently outcompetes the wild-type strain to reach high levels in the viral population. Resistance can be conferred by mutations that interfere with the ability of the drug molecule to inhibit the intended viral target. For example, for antivirals that block the fusion of viral particles with the target cell membrane, the mutations which confer resistance can alter the shapes and chemical properties of viral proteins to either prevent drug binding or allow the virus to enter the cell despite the presence of the drug.

There may, however, be another mechanism by which resistance can develop in viral populations. In a 2000 paper [16], Wahl and Nowak hypothesized that a heretofore unobserved effect, which they termed *cryptic resistance*, may prevent a viral population from being suppressed by an antiviral drug, without it needing to evolve the ability to alter drug binding or even complete its life cycle in the presence of the drug. This insight was motivated by the realization that drug levels are not constant during the course of viral treatment, and hence the viral fitness in the presence of the drug is also time-varying. Like most medications, antiviral drugs are administered in discrete doses of constant size separated by approximately equal time intervals. Shortly after a dose, the drug is absorbed and drug levels are high, and the relevant stage of the viral life cycle is maximally inhibited. However, between doses, drug levels decay, and may eventually reach low levels where they are no longer suppressive. This pattern repeats periodically over the course of treatment. These authors suggested that the virus could avoid the effects of the drug by always completing the targeted phase of its life cycle when drug concentrations are at a minimum. If the length of the viral life cycle is a mutable trait, then in the presence of drug treatment it may evolve to become approximately equal to the duration of time between successive doses. The virus population would then become synchronized with the pattern of drug levels. In this manner, the virus could sustain itself indefinitely by “hiding” from the highest concentrations of the drug, even though it has no means of counteracting the effects of drug molecules when they are present.

Lifecycle timing, at least in some bacteriophages, is known to be a mutable trait subject to life history trade-offs [17–24]. Separately, antibiotic “tolerance” in bacteria is contrasted with traditional “resistance” in that it only implies the ability to temporarily survive high drug levels [25]. Tolerance can be heritable and traced back to particular mutations, and in certain *in vitro* settings, can even involve changes to the timing of particular growth cycle-dependent life cycle stages [26, 27]. However, since Wahl and Nowak’s original paper, no *in vivo*, *in vitro*, or *in silico* studies have examined whether cryptic resistance, perhaps more aptly called “tolerance by synchronization”, could actually evolve during antiviral treatment.

Here, we use mathematical models to show that tolerance by synchronization can plausibly arise in a viral population subjected to antiviral treatment. We start by augmenting well-established models for viral infection dynamics to account for distinct phases of the viral life cycle. This model includes a maturation rate, which can take on different values that result in different viral life cycle lengths. Fluctuating drug concentrations are incorporated as a periodic time-dependent infectivity of the virus. We evaluate the success of this tolerance strategy via two different methods. First, we examine viral fitness in a single-strain infection by determining the growth rate and long-term infection size, and we compute the life cycle durations that optimize each of these values. Next, to predict how an infection evolves, we study competition between multiple strains. Specifically, we search for strains that can outcompete others and can persist despite drug treatment. We discover that strains that are synchronized to the drug dose schedule—that is, have life cycle durations that are a near-integer multiple of the time between drug doses—can dominate the infection and cause sustained treatment failure, therefore conferring tolerance. These strains would avoid detection by most genotypic drug resistance assays, which look for mutations only in the viral protein targeted by the drug therapy, and by *in vitro* phenotypic susceptibility assays, which apply constant, not periodic, drug levels. Our results suggest that new experimental methods are needed to identify this potentially important new mechanism of antiviral resistance.

## Model

### Virus dynamics

The standard viral dynamics model makes the simplifying assumption that infected cells produce new virus particles as soon as they are infected [28]. In reality, a virus must complete many stages of its life cycle before new virions are created. These stages may include uncoating of the viral particle, transcription and translation of the viral genome, copying of the viral genome, assembly of viral proteins, or even cell cycle-dependent events. While the exact steps involved in production vary among different viruses, all have in common a delay between infection of a cell and viral production [29]. This intracellular delay is a prerequisite for drug tolerance via synchronization.

There are two common methods for incorporating delays into a dynamic model: first, by introducing a series of maturation phases, each represented by a state variable, and second, by using delay differential equations. We explore both methods in this paper.

**Fig 1:**
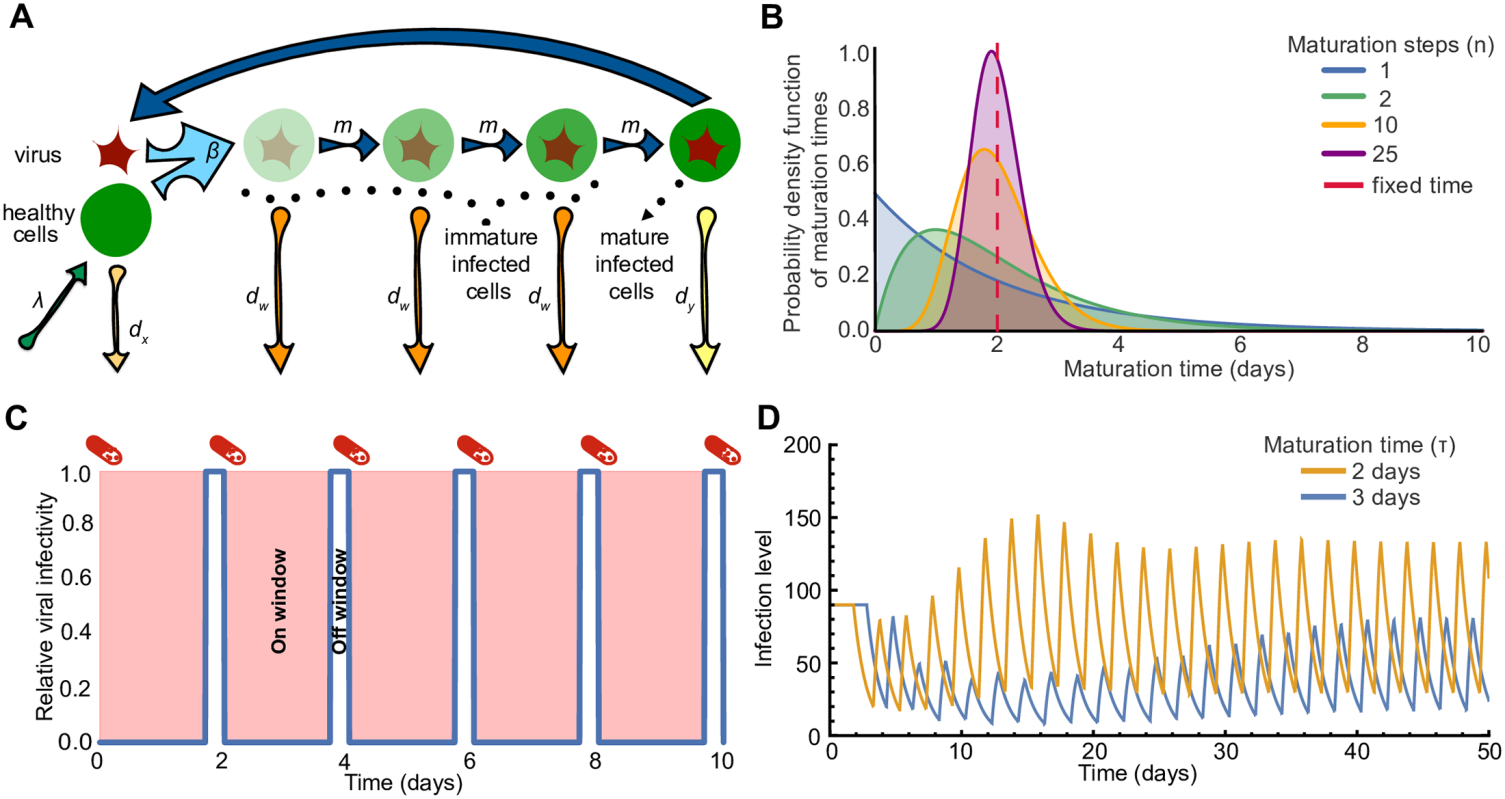
Schematic of model for viral dynamics in a patient undergoing antiviral treatment. **(A)** Diagram of basic viral dynamics model incorporating the infected cell maturation process. The population of cells is comprised of healthy cells *x*, one or more stages of immature infected cells *w*, and mature (virus-producing) infected cells *y*. The maturation time is the time it takes for an infected cell to pass through all the maturation phases. **(B)** Probability distribution functions of maturation times, when the maturation process happens as a series of *n* consecutive steps. The maturation times are gamma-distributed with the same average of 2 days, for different numbers of maturation steps (*n* = 1, 2, 10, 25). **(C)** The viral infectivity *β*(*t*) relative to the viral infectivity in the absence of the drug *β*_0_ fluctuates in response to drug levels (blue line). In the simple on-off model (a step function), drug levels are “on” for a fraction *f* of the time between doses (red shading), reducing viral fitness to zero, and “off” for the rest of the interval (viral fitness returns to baseline). **(D)** Time course of infection levels when the maturation time is fixed to *τ* = 2 days (yellow line) and *τ* = 3 days (blue line), and the drug dosage is modeled as a periodic step function. The synchronized strain (maturation time of 2 days, yellow line) reaches higher time-averaged infection level than the unsynchronized strain. In these examples, we use drug period *T* = 2 days and drug efficacy *f* = 0.85.

For the first method (Fig 1A), we posit a series of *n* immature phases *w*_*i*_, (similar to [30]):

$$\begin{array}{c} \begin{array}{cc} \dot{x}\left(t\right) & =\lambda -\beta \left(t\right)x\left(t\right)y\left(t\right)-d_{x}x\left(t\right)\\ {\dot{w}}_{1}\left(t\right) & =\beta \left(t\right)x\left(t\right)y\left(t\right)-\left(m_{1}+d_{w}\right)w_{1}\left(t\right)\\ {\dot{w}}_{i}\left(t\right) & =m_{i-1}w_{i-1}\left(t\right)-\left(m_{i}+d_{w}\right)w_{i}\left(t\right)\;\forall \;2\leq i\leq n\\ \dot{y}\left(t\right) & =m_{n}w_{n}\left(t\right)-d_{y}y\left(t\right) \end{array} \end{array}$$ (1)

The size of the population of healthy target cells is described by the state variable *x*; these cells are produced at rate λ and die at per capita rate *d*_*x*_. We consider the infected cell population as being subdivided into two subpopulations: “immature” (*w*_*i*_, where the subscript *i* indicates the particular immature phase if there are more than one) and “mature” (*y*) infected cells. Immature cells in phase *i* progress to become immature cells in phase *i* + 1 (or fully mature cells if *i* = *n*) at per capita rate *m*_*i*_, and immature cells in phase *i* die at per capita rate *d*_*w*_. Mature infected cells produce virus and lead to infection of healthy cells at rate proportional to the product of both their levels and the infectivity rate *β*(*t*), and die at per capita rate *d*_*y*_. We allow for the infectivity *β* to be time-dependent, since we assume that drug treatment acts on this parameter and that drug levels may vary over time (detailed in next section).

The maturation time *τ* is the time required for a newly infected cell in the first immature infected state *w*_1_ to progress and become fully mature in state *y*. We assume, for simplicity, that each maturation step occurs at the same rate, so *m*_*i*_ = *m*_*n*_ for all *i*. The probability distribution of maturation times is therefore a Gamma (Erlang) distribution [31] that depends on the number of maturation steps, *n*, and the rate constant, *m*_*n*_:

$$\begin{array}{c} p\left(\tau \right)=\frac{m_{n}^{n}\tau^{n-1}e^{-m_{n}\tau }}{(n-1)!} \end{array}$$ (2)

We set the maturation rate, *m*_*n*_, to be a linear function of the number of maturation steps, *n*, such that *m*_*n*_ = *nm*. With this choice of *m*_*n*_, the average maturation time is independent of the number of maturation steps (i.e., 〈*τ*〉 = *n*/*m*_*n*_ = 1/*m*). The standard deviation in maturation times, however, is inversely proportional to both the maturation rate, *m*, and the square root of the number of maturation steps (i.e., $\sigma_{\tau }=1/\left(m\sqrt{n}\right)$) (Fig 1B). Therefore, for a given number of intermediate phases, *n*, strains with lower expected maturation times (higher maturation rates) have less deviation in their time to maturation than strains with longer expected maturation times. Moreover, for a given maturation rate, *m*, more maturation steps would allow for the virus to better control its maturation time; in the limit of large *n*, the maturation time is fixed and equal to 1/*m*.

The second method for modeling intracellular delay uses delay differential equations with a fixed delay, *τ* = 1/*m*, between infection and start of virion production (similar to [32–34]):

$$\begin{array}{c} \begin{array}{cc} \dot{x}\left(t\right) & =\lambda -\beta \left(t\right)x\left(t\right)y\left(t\right)-d_{x}x\left(t\right)\\ \dot{y}\left(t\right) & =\beta \left(t-\tau \right)x\left(t-\tau \right)y\left(t-\tau \right)e^{-d_{w}\tau }-d_{y}y\left(t\right) \end{array} \end{array}$$ (3)

As for the basic viral dynamics model, we can define the basic reproductive ratio, *R*_0_, as the average number of new infections generated by a single infected cell over the course of its lifetime. If *β*(*t*) = *β* is constant, then, in the multi-stage model, *R*_0_ is given by

$$\begin{array}{c} R_{0}=\frac{\lambda \beta }{d_{x}d_{y}}{\left(\frac{nm}{nm+d_{w}}\right)}^{n} \end{array}$$ (4)

Similarly, in the fixed-delay model, *R*_0_ is given by

$$\begin{array}{c} R_{0}=\frac{\lambda \beta }{d_{x}d_{y}}e^{-d_{w}/m} \end{array}$$ (5)

In the limit of large *n*, the systems described by Eqs (1) and (3) are equivalent, and thus so are the expressions for *R*_0_ given by Eqs (4) and (5). If there is the possibility for an immature cell to die before producing virus (i.e., if *d*_*w*_ > 0), then *R*_0_ is maximized when the maturation rate, *m*, is maximized (so that infected cells mature as quickly as possible). If immature cells do not die (i.e., if *d*_*w*_ = 0), then *R*_0_ is independent of the maturation rate, *m*. As in the basic model, *R*_0_ is sufficient to qualitatively determine the outcome of the system of equations. If *R*_0_ > 1, then the infection will persist and reach an equilibrium, and if *R*_0_ < 1, then the infection will decline and eventually go extinct [30, 35]

For both models of viral dynamics, we also implemented a multi-strain competition between strains with different average maturation times (see S1 Text for equations). When *β*(*t*) = *β* is constant, an *R*_0_ value can be derived for each strain. When *d*_*w*_ = 0, all strains have the same *R*_0_ value and can co-exist at values that can be calculated analytically. When *d*_*w*_ > 0, the strain with the shortest maturation time has the highest *R*_0_ value and eventually outcompetes all other strains.

In addition, we also created stochastic versions of each of the single- and multi-strain models, where the rate of each reaction is equal to that in the differential equation formulation, and each reaction increases or decreases a state variable by 1. The stochastic process was simulated with the Gillespie next reaction method [36]. In stochastic versions of the model, extinction can happen even when *R*_0_ > 1.

Note that throughout this paper, we use a common assumption to simplify the viral dynamics model and reduce the computational burden of simulations. For the vast majority of infections, the dynamics of free virions tend to be much faster than the dynamics of cells. Virus is produced in large quantities and is rapidly cleared *in vivo*. This implies that the free virus population tends to reach a “quasi-steady state” level with respect to the level of infected cells, implying that a separation of timescales can be applied to the system. Consequently, we do not explicitly track free virus but instead assume that its level is proportional to the abundance of infected cells. With this assumption, our infectivity parameter *β*(*t*) is actually a composite parameter given by *β*(*t*) = *b*(*t*)*k*/*c*, where *b*(*t*) is the infectivity of a free virion, *k* is the rate constant for production of free virus by mature infected cells, and *c* is the rate constant for clearance of free virus.

Ordinary differential equations were numerically integrated with the Scipy *odeint* algorithm in Python 2.7 [37], and delay differential equations were numerically integrated with the Scipy-based DDE solver *ddeint* [38]. For results presented in the main text, we choose parameter values that roughly correspond to HIV infection (Table 1). Throughout the paper, we often present results for the case of no death of immature infected cells (i.e., *d*_*w*_ = 0), since in this case, the value of *R*_0_ in the presence of constant drug levels is independent of the maturation rate, *m*, and the number of maturation stages, *n*, which makes it easiest to see how *R*_0_ changes under periodic drug levels. However, we also present results for cases with other values of *d*_*w*_, including the most natural assumption: that immature infected cells die at the same rate as uninfected cells (i.e., *d*_*w*_ = *d*_*x*_). Since immature infected cells are not yet producing new virions, they are less likely to a) experience direct cytotoxic effects of virus production and release, and b) be presenting viral epitopes and triggering cytolytic immune responses, so their death rate may resemble that of healthy uninfected cells.

**Table 1:** Parameter values used in the viral dynamics and drug treatment models.

Parameter	Description	Value	Units
λ	Input rate of healthy cells	100	cell conc^†^. ⋅ days^−1^
*d*_*x*_	Death rate of healthy cells	0.1	days^−1^
*β*_0_	Infection rate in absence of drug	0.01	cell conc.^−1^ days^−1^
*m*	Maturation rate of immature cells	varied	days^−1^
*d*_*w*_	Death rate of immature cells	0 or 0.1	days^−1^
*d*_*y*_	Death rate of mature cells	1.0	days^−1^
*n*	Number of maturation steps	varied	–
*R*_0_	Basic reproductive ratio in absence of drug (*d*_*w*_ = 0), Eqs (4) and (5)	10	–
*T*	Time between drug doses	2	days
*f*	Fraction of time drug is active *	varied	–
*IC*_50_	Drug concentration at which 50% inhibition occurs **	0.05	conc.
*M*	Steepness of dose-response curve **	1.0	–
*C*_max_	Steady-state peak drug concentration **	8.0	conc.
*t*_*h*_	Half-life of drug decay **	varied	days
Ω	System volume ^‡^	5.0	–

* Parameter used for the simple on-off drug dynamics.

** Parameters used for the pharmacological drug dynamics. When compared directly, time-averaged drug efficacy was set to be the same for both models.

^†^ We roughly base these parameter values on HIV infection (e.g. [39]). Cell concentrations would be measured per microliter (*μL*) of blood.

^‡^ When the parameters for the stochastic simulations were initiated, the overall population size was increased by a factor of Ω, such that λ_*new*_ = *Ωλ* and *β*_*new*_ = *β*/Ω. The Gillespie stochastic simulations were performed using the scaled parameter values.

### Drug therapy

We model the effect of an antiviral drug on the infection by assuming that it reduces the infectivity in a manner that depends on the drug concentration, *D*(*t*), which varies over time. We first model the periodic time-dependence of drug levels as an on-off switch. The drug is taken every *T* days and completely inhibits new infections for a time *fT* thereafter, such that *β*(*t*) = 0 for that time; we refer to this time as the *on window*. For the remaining time between doses, we assume the drug to be completely inactive, such that *β*(*t*) = *β*_0_ for that time; we refer to this time as the *off window*. We also refer to *f* as the efficacy of the drug; a perfect drug therapy would have efficacy equal to 1 (see Fig 1C):

$$\begin{array}{c} \begin{array}{cc} D(t) & =\{\begin{array}{cc} 1,\; & \text{if}\;t\;\mathrm{mod}\;T<fT\\ 0,\; & \text{if}\;t\;\mathrm{mod}\;T>fT \end{array}\\ \beta (t) & =\beta_{0}\left(1-D\left(t\right)\right) \end{array} \end{array}$$ (6)

We also considered drug dynamics that follow a simple pharmacological model. When the drug is taken consistently, drug levels *D*(*t*) peak immediately at *C*_max_ following each dose and then decay exponentially with half-life *t*_*h*_, reaching a minimum value $C_{\min}=C_{\max}2^{-T/t_{h}}$, before the next dose. Infectivity is reduced in a concentration-dependent manner that is described by a Hill dose-response curve, where *β*_0_ is the infectivity in the absence of treatment, *IC*_50_ is the concentration at which 50% inhibition occurs, and *M* quantifies the steepness of the decay curve:

$$\begin{array}{l} D\left(t\right)=C_{\text{max}}2^{-t/t_{h}}\\ \beta \left(t\right)=\frac{\beta_{0}}{1+{\left(\frac{D\left(t\right)}{IC_{50}}\right)}^{M}} \end{array}$$ (7)

Drug efficacy can be varied in this model by changing *C*_max_, *t*_*h*_, *IC*_50_, or *M*. However, wherever Eq (7) are used, we choose to vary *t*_*h*_ only. For any parameter combinations used in this model, we can always find a corresponding *f* value for the on-off model in Eq (6) such that the time-average rate of infection is equal between the two models. Indeed, throughout the paper, we express overall drug efficacy values using the single quantity *f* for both models.

In addition to modeling perfect adherence to periodic drug treatment, we consider imperfect adherence. We assume there is a fixed probability of taking each dose at the scheduled time or missing it completely, and that each dose is taken independently. In the on-off model, a missed dose results in a full drug period with *D*(*t*) = 0. In the pharmacological model, drug continues to decay after a missed dose, and a subsequent dose increases the concentration by Δ = *C*_max_ − *C*_min_. Example values of *R*_0_(*t*) under this model are shown in S1 Fig.

When viral fitness is time-varying, as is the case under fluctuating drug concentrations, the above calculations for *R*_0_ no longer hold. A time-averaged *R*_0_ value (${\bar{R}}_{0}$) could be calculated that takes into account the time-dependence of the infectivity, ${\bar{R}}_{0}=R_{0}\left(\langle \beta \left(t\right)\rangle \right)$. However, as we will show below, this average *R*_0_ is no longer sufficient to describe the outcome of the model.

## Results

### Simulation results

#### Synchronized virus reaches highest infection size in a deterministic single-strain model

We first evaluated the performance of individual strains in the presence of fluctuating drug concentrations, as a function of their maturation time. We considered both the multi-step maturation process (Eq (1)), in which maturation times are exponentially- (*n* = 1) or Gamma- (*n* > 1) distributed, and the fixed-delay maturation model (Eq (3)). Drug kinetics were given by the simple on-off model, Eq (6). The initial condition was taken as the drug-free equilibrium of the system, which includes infected cells in all maturation phases (formulas given in S1 Text). The equilibrium infection level in the presence of periodic drug levels was determined by numerically integrating the differential equations until a steady-state was reached.

**Fig 2:**
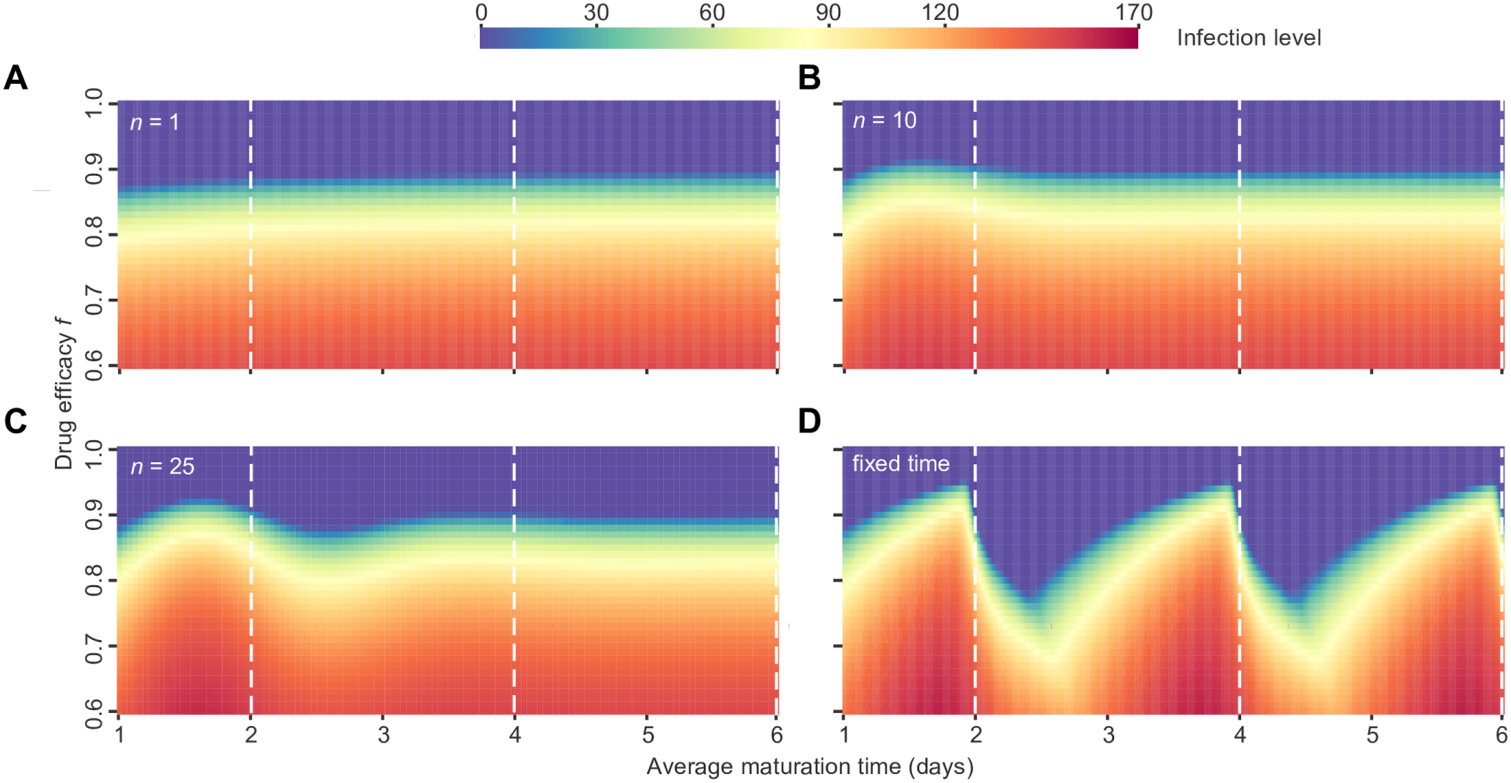
Equilibrium infection level for the single-strain deterministic model, as a function of the average maturation time. Viral dynamics were simulated under periodic antiviral therapy given by the simple on-off model with a period (*T*) of 2 days and varying drug efficacy (*f*). The infection level (heat map color) is measured as the concentration of mature infected cells (*y*) once a steady-state has been reached. Each calculation included only a single virus strain with average maturation time 1/*m* (maturation rate of *nm* for each stage). **(A)** Results with *n* = 1 maturation steps. **(B)** Results with *n* = 10 maturation steps. **(C)** Results with *n* = 25 maturation steps. **(D)** Results with fixed maturation time *τ* = 1/*m*. The white dotted lines show where the average maturation time is equal to an integer multiple of the drug period. For all shown simulations, we assume the death rate of immature cells to be zero (*d*_*w*_ = 0). Results shown for 41 different drug efficacies between *f* = 0.6 and *f* = 1.0, for 101 different strains with average maturation times between 1 and 6 days. A version of the results with more resolution around the threshold drug efficacy (*f* = 0.9) is in S2 Fig and a version with *d*_*w*_ > 0 is in S3 Fig.

When the maturation process happens in a single step (*n* = 1, exponentially-distributed times), we do not observe any benefit for synchronized strains (Fig 2A). The infection size increases slightly with the average maturation time. The intuition for these results is as follows: Since there is a large variability in the maturation time (Fig 1A), infected cells cannot reliably mature during the time the drug is off, especially when the drug efficacy is high and this time is relatively short. If they cannot die before maturing (*d*_*w*_ = 0), the safest strategy is for them to linger in the immature phase as long as possible. That way, they are unlikely to die in the mature infected state without producing any offspring, while waiting for the inhibitory effect of the drug to disappear. This is in contrast to the constant drug level case, in which equilibrium infection size is independent of the maturation time (see S1 Text).

When the maturation time is fixed (dynamics given by Eq (3)), the results change dramatically (Fig 2D). For any drug efficacy, infection levels are highest for viral strains with maturation times near an integer multiple of the drug period. These *synchronized* strains match both the frequency and phase of their life cycle with the drug dosing period, allowing them to persist at drug levels which cause other strains to go extinct, therefore causing *tolerance by synchronization*. Steady-state infection levels seem to only depend on the relationship of the maturation time to the drug period (1/*m* mod *T* or *τ* mod *T*), and not the absolute value of the maturation time itself. However, if there is a fitness cost to lingering in the immature phase longer than necessary, which occurs when immature cells can die (*d*_*w*_ > 0), all “synchronized” strains are not equal: those with maturation times near higher integer multiples of the dosing period reach lower infection levels (S3 Fig). Overall, strains are eliminated at lower drug efficacies.

When the variability in maturation times is intermediate, synchronized strains still do best (Fig 2B and 2C). All synchronized strains are no longer equal: those with average maturation time close to the lowest possible integer multiple of the drug dosing period have the highest advantage relative to neighboring strains in phenotype space. This occurs because as the average maturation time increases, so does its variance ($\sigma_{\tau }=\langle \tau \rangle /\sqrt{n}$), which lowers infection levels in the presence of periodic drug levels. Note that even with many maturation steps, the variance is still quite large (*σ*_*τ*_ = 0.63 for *n* = 10 and 0.4 for *n* = 25 when *m* = 0.5), and so high *n* may best represent a realistic level of control over life cycle length.

#### Synchronized virus outcompetes others in a deterministic multi-strain model

The above results identify a benefit of life cycle synchronization, but do not directly address whether synchronization would emerge in a heterogeneous viral population. To investigate this question, we evaluated whether strains with certain maturation times could outcompete others in the presence of fluctuating drug concentration. Using a multi-strain version of the model (see S1 Text), we started with an initial condition that included all viral strains distributed over all maturation rates. Then, we integrated the differential equations under periodic drug treatment until a steady state was reached. Between 50 and 500 strains were competed for each scenario.

**Fig 3:**
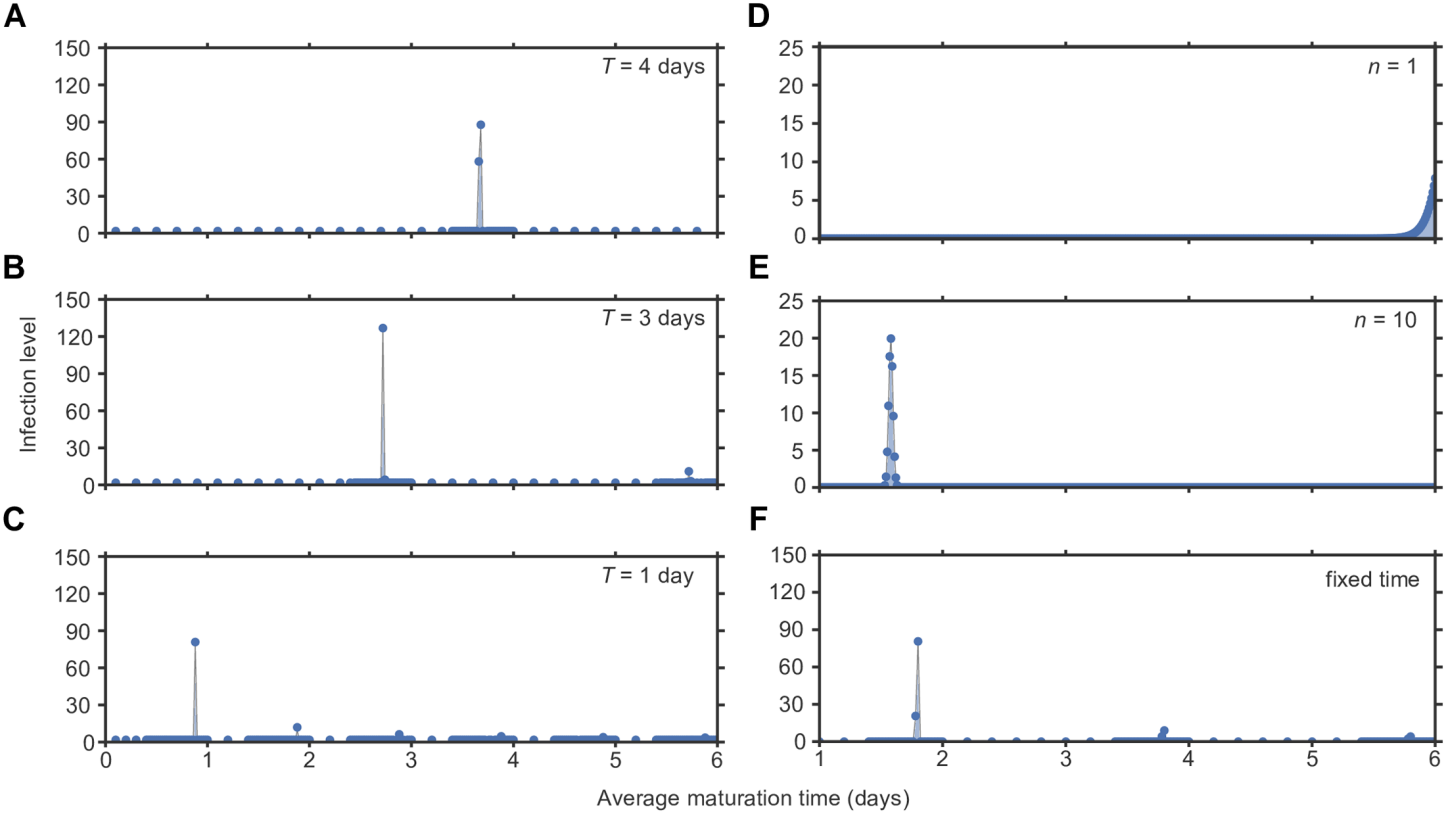
Competition between viral strains with different life cycle times. Viral dynamics were simulated under periodic antiviral therapy given by the simple on-off model with a drug efficacy of 85% (*f* = 0.85). The infection level (y-axis) is measured as the concentration of mature infected cells (*y*) once a steady-state has been reached. Each simulation included a collection of viral strains with the full range of maturation times shown. Each strain is labeled by its average maturation time 1/*m* (maturation rate of *nm* for each stage). **A-C** Fixed maturation time, varying drug dosing period (*T*). **(A)** Drug dosing period *T* = 4 days. **(B)** Drug dosing period *T* = 3 days. **(C)** Drug dosing period *T* = 1 day. **D-F** Drug dosing period (*T*) of 2 days, varying distribution of maturation times. **(D)** Results with *n* = 1 maturation step. **(E)** Results with *n* = 10 maturation steps. **(F)** Results with fixed maturation time *τ* = 1/*m*. For all shown simulations, we assume the death rate of immature cells to be zero (*d*_*w*_ = 0). Data shown for competitions between 57, 85, 165, 501, 501, and 108 different strains, for panels from **A** to **F** respectively, with average maturation times between 1 and 6 days.

The results of the multi-strain competition agreed with the results from individual single strains: The strain(s) that reached highest viral loads when infecting alone were also the strains that won the competition against others (Fig 3), reaching persistent and high infection levels. Therefore, heterogeneous viral populations can evolve towards drug tolerance via synchronization. When the maturation time was fixed or tightly controlled, the surviving strains have maturation times close to integer multiples of the period of drug intake (Fig 3A, 3B, 3C and 3F). Contrary to what we would predict based on the single-strain model, we find that when multiple synchronized strains with different maturation times co-exist at equilibrium, it is the strains with the *lowest* maturation times that reach the highest infection size. This is likely because their faster transition through the infection cycle makes them able to more quickly infect the target cell pool that all viral strains compete for.

**Fig 4:**
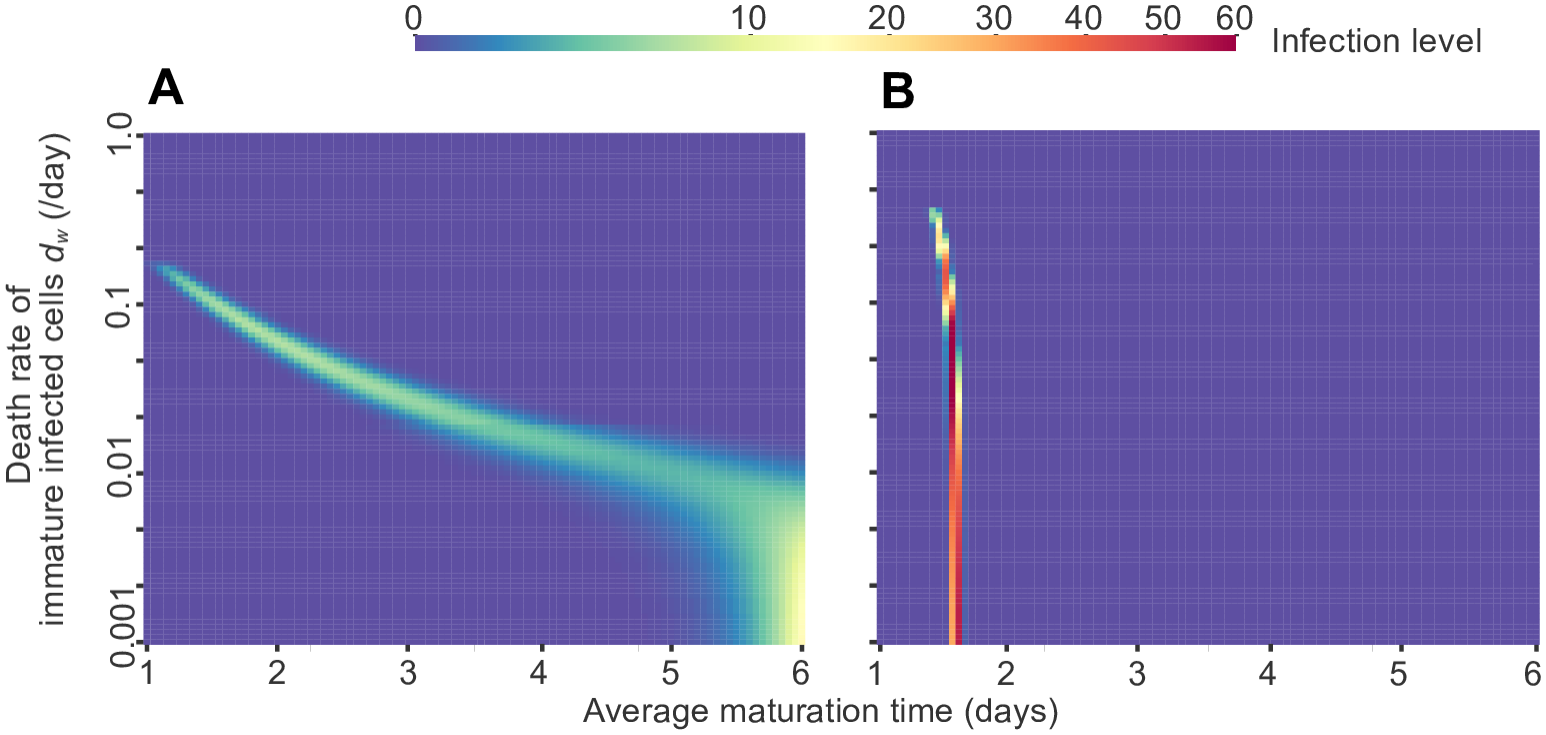
Competition between viral strains with different life cycle times when infected cells can die before maturing. Viral dynamics were simulated under periodic antiviral therapy given by the simple on-off model with a period (*T*) of 2 days, a drug efficacy of 85% (*f* = 0.85), and an immature cell death rate between 0 and *d*_*y*_ (mature infected cell death rate). The infection level (heat map color) is measured as the concentration of mature infected cells (*y*) once a steady-state has been reached. Each simulation included a collection of viral strains with the full range of maturation times shown, all with the same death rate of immature infected cells. Each strain is labeled by its average maturation time 1/*m* (maturation rate of *nm* for each stage). **(A)** Results with *n* = 1 maturation step. **(B)** Results with *n* = 10 maturation steps. Data shown for 110 different immature cell death rates between *d*_*w*_ = 0.001 and *d*_*w*_ = 1, for competitions between 501 different strains with average maturation times between 1 and 6 days.

We again consider the case where infected cells can die while still maturing (*d*_*w*_ > 0), so that there is a fitness cost to lingering in the immature phase longer than necessary. When the life cycle length cannot be well controlled—as in, the maturation process has a low number of steps—then the higher the chance of dying during the maturation phase, the lower the optimal maturation time (Fig 4A). On the other hand, when the maturation time can be well controlled through a high number of maturation steps, the surviving strains remain synchronized and reach higher infection levels (Fig 4B).

#### Synchronized virus fixes in a multi-strain stochastic model

One of the drawbacks of analyzing strain competition and survival using a deterministic model is that such implementations allow for the viral concentrations to become arbitrarily low during the treatment, without the strains ultimately becoming extinct. Therefore, we explore whether synchronization could happen in the realistic situation where viral dynamics occur stochastically, population sizes are finite, and extinction is possible.

We implemented a stochastic simulation of the multi-strain competition model, in which the expected rate of each biological process had the same value as its rate in the deterministic implementation. The initial conditions were based on the drug-free equilibrium and included all viral strains distributed over all maturation rates. The total system size was scaled up from the deterministic implementation (Table 1) so that all infection stages had non-zero integer values initially. We repeated each simulation until only one strain survived (i.e. “fixed”) when taking into account infected cells in all phases of the life cycle, and continued until it reached a steady state (or went extinct). Since we were interested in which strains were most likely to survive treatment and dominate in the population, we recorded the proportion of simulations in which a strain was the last surviving in the population and continued on to reach a steady state. This fixation probability is a standard measure of evolutionary success in population genetics [40] and evolutionary dynamics [41].

**Fig 5:**
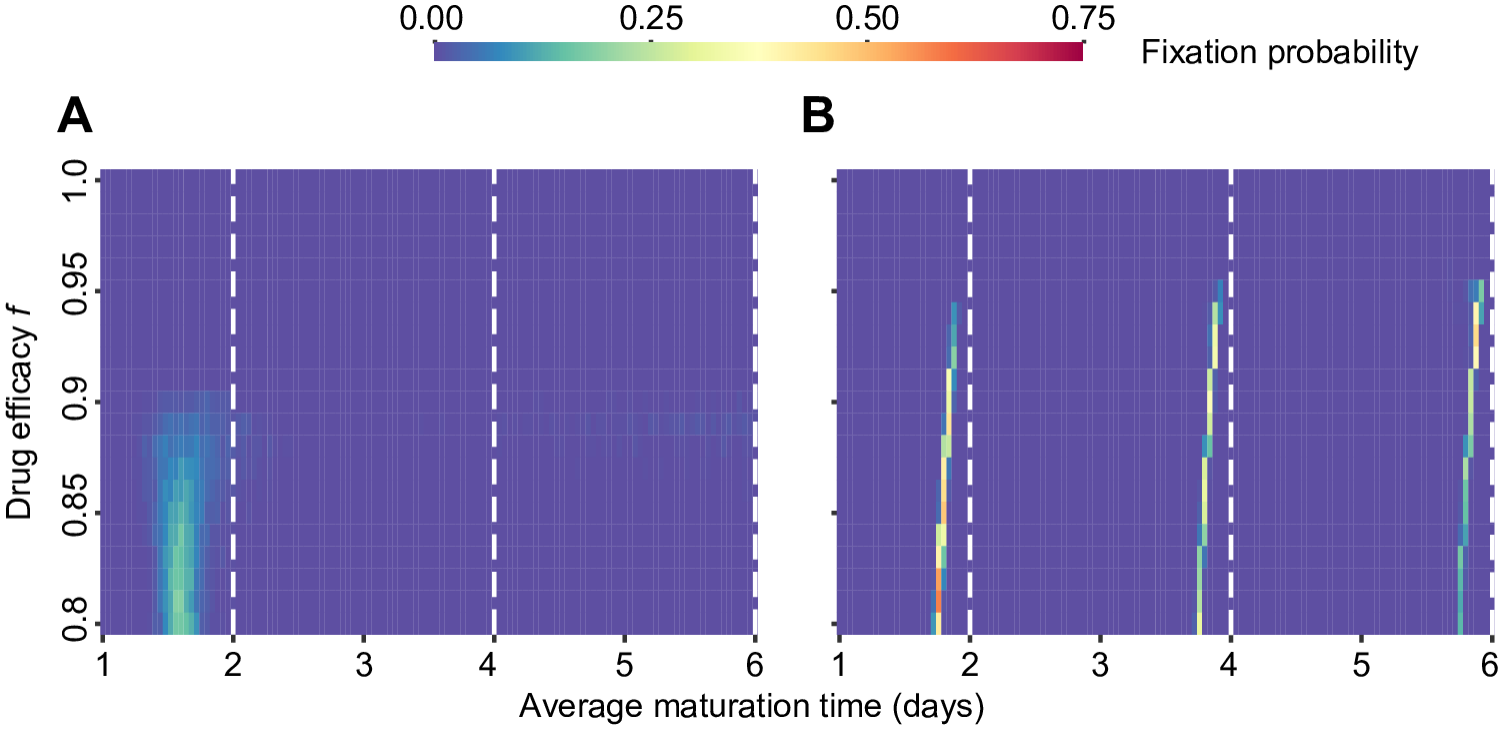
Stochastic competition between viral strains with different life cycle times. Viral dynamics were simulated under periodic antiviral therapy given by the simple on-off model with a period (*T*) of 2 days and varying drug efficacy (*f*). The fixation probability (heat map color) is measured as the fraction of simulations in which a strain was the last surviving in the population and continued on to reach a steady state. **(A)** Results with *n* = 10 maturation steps. **(B)** Results with fixed maturation time *τ* = 1/*m*. The white dotted lines show where the average maturation time is equal to an integer multiple of the drug period. For all shown simulations, we assume the death rate of immature cells to be zero (*d*_*w*_ = 0). 500 simulations were run for each drug efficacy level. Data shown for 21 different drug efficacies between *f* = 0.8 and *f* = 1.0, for competitions between 126 strains with average maturation times between 1 and 6 days. A version of the results for all drug efficacies is in S4 Fig, and a version with *d*_*w*_ > 0 is in S5 Fig.

Overall, the strains with the highest fixation probability are the same as the optimal strains in the deterministic model (Fig 5). However, strains with a broader range of maturation times are able to fix than those that survived in the multi-strain deterministic calculation, since occasionally the true optimal strain will go extinct before being able to fix. Additionally, when maturation time is not perfectly controlled, there are strains that could survive at high drug efficacy when alone (Fig 2) but rarely fixed in a competition (Fig 5). We observed that whichever strain survived the competition at these high drug levels was often at too low of a level to ultimately recover and reach a steady state level. Mirroring the deterministic model, even when the death rate of immature infected cells (*d*_*w*_) is non-zero, strains with the lowest (synchronized) maturation times survive and fix (S5 Fig). Specific to the stochastic model, we saw that if maturation time is fixed and drug efficacy is high, sometimes strains with higher (synchronized) maturation times can survive. The fixation time did not vary much with drug efficacy, except that as expected, it increased substantially when the drug efficacy was very low, as many strains have relatively equal fitness and non-zero fixation probability (S4 Fig).

#### Synchronization is robust to realistic drug kinetics and imperfect adherence

The results of the model are overall maintained when simulating realistic drug kinetics via the simple pharmacological model. The main difference is that the maturation process needs to happen in a higher number of steps than in the case of on-off kinetics, in order for synchronization to occur. Nevertheless, when the number of maturation steps is high (*n* = 25) or the maturation time is fixed, synchronized strains are still able to persist at high drug levels when alone (S6 Fig) or when competing with others (S7 Fig).

Additionally, previous studies have explored the effects of imperfect adherence on the success of antiviral therapies, and showed that non-adherence can lead to treatment failure [42]. When considering life-cycle synchronization, imperfect adherence increases the variance in the times between doses, and therefore may have an impact on the ability of strains to control their life cycle length so as to match the period of the drug. We observe that when maturation time is variable, there is a critical adherence level below which synchronization no longer occurs, and instead, the strain with the highest maturation time is selected (for example, over 80% adherence is needed for synchronization when *n* = 10) (S8A Fig). Effectively, low adherence seems to push results in the same direction as higher variance in maturation time would (lower *n*). When the maturation time is fixed, two switches occur as adherence levels drop. For imperfect adherence as low as 40%, synchronized strains with high maturation times will outcompete those with shorter maturation times (S8B Fig). When adherence is very low (< 10%), other unsynchronized strains will also survive, as the selective effect of the drug treatment is greatly reduced (the drug is taken only 1 out of 10 times).

Finally, we test the realistic scenario in which a patient is perfectly adherent (takes 100% of their prescribed pills), but does not take each dose at exactly the prescribed time (so the time between doses varies). We assume that the doses are still—on average—taken every *T* = 2 days, yet the precise time of the dose is normally-distributed with standard deviation *σ* between 36 minutes and 6 hours. Similar to the baseline case, the drug is effective a time *fT* after each dose, where *f* is the drug efficacy. When the maturation time is variable (*n* = 10), we observe synchronization is maintained even when *σ* = 1.5 hours (S9 Fig). More importantly, when the maturation time is fixed, some synchronized strains survive even when *σ* = 6 hours. Therefore, life cycle synchronization could occur even if the drug is taken at variable times, with the same average dosing period.

#### Synchronization occurs despite viral life cycle trade-offs affecting maturation time

We additionally consider a scenario where there are fitness trade-offs controlling the viral life cycle length, independent of the periodic nature of drug treatment. Our model is augmented so that some type of precursor compound is produced during the immature phase and is required for production of infectious virions in the mature phase (details in S1 Text). Consequently, there is a cost to shorter maturation times, since less precursor will be produced and so the cell will be less infectious upon maturation. In the absence of treatment, and when immature cells don’t die (*d*_*w*_ = 0), *R*_0_ is maximized in this model for longer maturation times. If immature cells can die (*d*_*w*_ > 0), then there is an optimal life cycle length (*τ*_*c*_) that balances precursor production and survival. This finding is reminiscent of models of trade-offs in the evolution of lysis time in bacteriophage [19]. We examined the results of this model under periodic treatment (S10 Fig), using the multi-strain deterministic model with fixed maturation time. When we chose parameters such that the optimal life cycle length in the absence of drugs was close to the dosing period (S10C Fig), we found that the optimum remained the same under periodic drug levels. If we instead chose the treatment-free optimum to be most out of sync with the dosing period (e.g. *T* = 2, *τ*_*c*_ = 3), then we still found that periodic drug treatment selected for synchronized strains, although it might be a strain with a life cycle near a higher integer multiple of the dosing period and that the equilibrium infection level would be lower (S10D Fig). In the case where the treatment-free optimum was near the maximum maturation time simulated, then the strain with the life cycle that was the highest available integer multiple of the drug period dominated (S10E Fig). Overall, these results show that even when there are costs to life cycle length, synchronized strains are optimal. Other models could be constructed to include specific biochemical pathways known to be important for particular viruses (e.g. [43]).

#### Synchronized strains have maturation times slightly less than the drug dosing period

Our results so far have shown that a variety of modeling methods and biological assumptions result in the ability of “synchronized” viral strains to survive drug levels and outcompete other strains. In all cases we have found that the best-performing strain has maturation time near, but not exactly equal to, the drug dosing period. In fact, we consistently find that the optimal maturation time is slightly less than the drug period (S11 Fig). This is mainly because in addition to the maturation time, the total viral generation time also includes the time for virions to be released by a mature infected cell and to contact and infect a healthy target cell. The timing of this second step cannot be controlled by the virus—it can occur throughout the remaining lifetime of an infected cell (which has a random duration in our model) proportionally to the drug-dependent viral fitness at that point. There is no simple way to calculate the length of this post-maturation phase of the life cycle from the model, but we can understand some of its properties.

Consider the case where the maturation time is fixed and the drug follows simple on-off kinetics. A mature infected cell is equally likely to cause a secondary infection at any point during its lifespan when the drug is “off”. If the lifespan of a mature infected cell (1/*d*_*y*_) is much longer than the time the drug is off (*T*(1 − *f*)), then this “off-window” is what limits the time during which new infections can occur. The average time until secondary infection, and therefore the offset between the optimal maturation time and the drug-period, is the midpoint of this interval: *T*(1 − *f*)/2. This trend can be seen in Figs 2D and 3B, and S11 Fig; the optimal maturation time increases slightly with drug efficacy *f*. However, if the death rate of mature infected cells is large (1/*d*_*y*_ > *T*(1 − *f*)), then the mature infected cell lifespan is instead the limiting factor in the time to new infections. In this case, the offset is smaller and given by ≈ 1/(2*d*_*y*_) (S11 Fig). When drug dynamics instead follow a more realistic pharmacologic model, the offset is lower and doesn’t have this predictable dependence on drug efficacy. If viral maturation time is more variable, then the optimal maturation time is even further below the drug period than expected, suggesting that there is some asymmetry which makes it less costly to mature too early versus too late.

Note that in this paper, we purposefully chose biologically realistic parameters such that maturation was the dominant component of the viral life cycle. In this case, maturation is the rate-limiting step in infection; infected cells that could tightly control maturation could implicitly control their overall life cycle length. We could imagine a related model in which the rate-limiting viral life cycle process is that of releasing virions to infect new target cells, with a different mechanism to control life cycle timing (e.g., regulate the rate and timing of virion release).

### Analytical results

#### Basic reproductive ratio fails to predict emergence of life cycle synchronization

The efficacy of antiviral drugs is measured by how well they can reduce the viral fitness; this is usually quantified by determining the basic reproductive ratio *R*_0_ of the virus during the treatment. Since *R*_0_ represents the *average* offspring of an infected cell, the concept can be extended to the case of fluctuating drug concentrations *D*(*t*), by averaging the drug-dependent *R*_0_ over time: ${\bar{R}}_{0}\left(D\right)=\int R_{0}\left(D\left(t\right)\right)dt$. Therefore, in the case of on-off drug dynamics, ${\bar{R}}_{0}\left(D\right)=R_{0}\left(1-f\right)$, where *f* is the drug efficacy (fraction of time that drug is on) and *R*_0_ is the basic reproductive ratio in the absence of drug.

However, our results show that as a result of synchronization, ${\bar{R}}_{0}\left(D\right)>1$ is no longer necessary or sufficient for a viral strain to persist despite therapy. In Fig 2C and 2D, for which *R*_0_ = 10, one can see that when the viral life cycle is well controlled, strains with values of ${\bar{R}}_{0}\left(D\right)<1\;\left(f>0.9\right)$, which would be expected to go extinct, can actually survive when their life cycle time is close to an integer multiple of the drug period (*m* = 2, 4, 6, …). This effect can be seen even more clearly in a zoomed-in version of the figure (S2 Fig). These strains can also outcompete others, fix in the population, and avoid stochastic extinction (Fig 3). On the other hand, strains with life cycle times most out of sync with the drug period (*m* = 1, 3, 5, …) cannot persist despite ${\bar{R}}_{0}\left(D\right)>1$ (*f* < 0.9).

**Fig 6:**
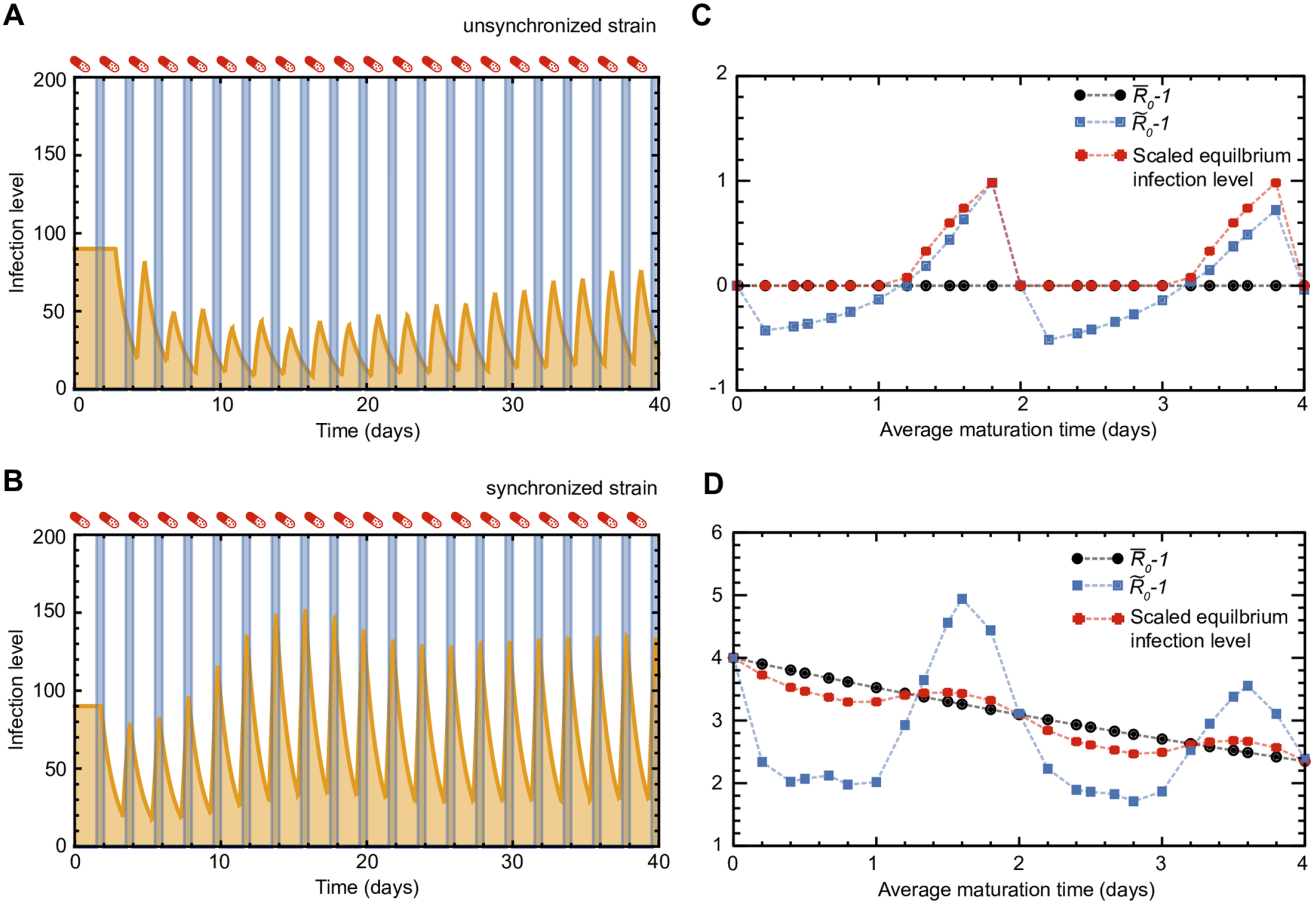
Modified basic reproductive ratio ${\tilde{R}}_{0}$, unlike the time-averaged ${\bar{R}}_{0}$, accurately predicts infection outcome under a periodic drug treatment. **(A)** Time course of infection levels (concentration of mature infected cells, *y*(*t*)) for an unsynchronized strain (*T* = 2 days and *τ* = 5 days). The maturation time is fixed and drug levels are modeled as a periodic step function. Unsynchronized strains are more exposed to the drug effects, as they overlap less with the off-windows of the drug treatment. **(B)** Synchronized strains (*τ* = 2) are less exposed to the drug effects, as they overlap with the off-windows of the drug treatment. The off-windows in the drug treatment are represented by blue shading. In this example, we use drug period *T* = 2 days and drug efficacy *f* = 0.75. **(C)** The time-averaged basic reproductive ratio minus one (${\bar{R}}_{0}-1$) is plotted versus the maturation time (*τ*) for a fixed drug efficacy and the simple on-off model of drug levels (black line). ${\bar{R}}_{0}$ is independent of maturation time when immature cells do not die (*d*_*w*_ = 0 here) and weakly dependent for *d*_*w*_ ≪ *m*. ${\bar{R}}_{0}<1$ versus ${\bar{R}}_{0}>1$ is unable to explain the observed persistence versus extinction of viral strains (e.g. Fig 2). We derived a new quantity, ${\tilde{R}}_{0}$ (blue line), which works in the presence of synchronization to describe the observed behavior. ${\tilde{R}}_{0}$ was obtained via numerical solution of Equation (S.99) for ${\tilde{r}}_{0}$ and substitution of ${\tilde{r}}_{0}$ into Equation (S.100). The equilibrium infection level for a single strain (red line) is scaled to match ${\tilde{R}}_{0}-1$ at *τ* = 1.8. The drug efficacy is set to *f* = 0.9, and the death rate of immature infected cells is zero (*d*_*w*_ = 0). **(D)** Same as (C), except the drug efficacy is set to *f* = 0.5, and the death rate of immature infected cells is non-zero (*d*_*w*_ = 0.1). The equilibrium infection level is scaled to match ${\tilde{R}}_{0}-1$ at *τ* = 0. Connecting lines between points are drawn as a guide for the eye.

This limitation of ${\bar{R}}_{0}\left(D\right)$ can be understood by considering a key assumption it makes: that an infected cell is equally likely to mature at any point in the drug period, and at that point, infectivity is determined by the drug level, *β*(*D*(*t*)). If that were true, then the time-average of the infectivity alone would determine the average basic reproductive ratio. In reality, under periodic drug levels, infection levels also become periodic, and drug levels near the peak of the infection level (when more infected cells mature and more new infection events can occur) are more important than drug levels near the trough infection levels. Time courses of infection for unsynchronized and synchronized strains (Fig 6) show that on average, synchronized strains are less exposed to the drug effects than other strains, because they reach peak infection levels during low drug concentrations. For this reason, it is necessary to determine a quantity analogous to *R*_0_ that could work even in the case of viral synchronization, or at least a way to numerically predict whether a viral strain could survive in the presence of fluctuating drug levels.

#### Modifying the basic reproductive ratio to predict emergence of life cycle synchronization

We derive an analytic expression for a measurement of viral fitness analogous to the basic reproductive ratio *R*_0_. This expression can be numerically integrated, and is sufficient to predict the persistence or eradication of viral strains subjected to a periodic drug treatment. The details of this calculation are given in S1 Text, but summarized here. Working with the delay differential equation formulation of a fixed maturation delay in the viral life cycle (Eq (3)), the basic reproductive ratio in the absence of drug (or presence of a constant drug level) is given by Eq (5). In early infection, before target cells are limited, viral load grows or decays exponentially at rate *r*_0_, where *r*_0_ is the solution to the equation [35]:

$$\begin{array}{c} R_{0}=(1+\frac{r_{0}}{d_{y}})e^{r_{0}\tau } \end{array}$$

We assume that the dynamics for the periodic drug level case follow similar form. In particular, for a single-strain with a fixed time-delay and periodic drug dosage, the infection rate is also periodic such that *β*(*t*) = *β*(*t* + *T*) where *T* is the period of the drug-dosage regime. We use the following expression for the density of mature infected cells in our analysis, which is a solution for the dynamics in the time regime of interest (see S1 Text):

$$\begin{array}{c} y\left(t\right)=Y\left(t\right)e^{{\tilde{r}}_{0}t} \end{array}$$

where ${\tilde{r}}_{0}$ is an exponential growth rate, and *Y*(*t*) is also a function with period *T*. If ${\tilde{r}}_{0}<0$, then, when sampled at times that are integer multiples of the drug-dosing period, the density of infected cells decreases in time, and the virus is eventually eliminated. We define a quantity, ${\tilde{R}}_{0}$, as follows:

$$\begin{array}{c} {\tilde{R}}_{0}=(1+\frac{{\tilde{r}}_{0}}{d_{y}})e^{{\tilde{r}}_{0}\tau } \end{array}$$

We derived an expression for ${\tilde{r}}_{0}$ in terms of the infection parameters and the drug period *T*. Our results show that ${\tilde{R}}_{0}>1$ varies with maturation time in agreement with Fig 2, and is a necessary and sufficient condition for a viral strain to persist during a drug therapy with periodic dosage. While extensively characterizing the utility of ${\tilde{R}}_{0}$ is beyond the scope of this paper, this analysis paves the way for a more detailed analytical exploration of the system and its regimes. Finally, we also obtained a solution for ${\tilde{r}}_{0}$ for the case of a finite number of maturation steps *n*.

## Discussion

In this paper, we showed that it is possible for a virus population to synchronize its life cycle with the pattern of drug therapy. This process allows for strains to persist and cause treatment failure during anti-viral treatments where success would be expected. Although originally called “cryptic resistance” when it was first proposed by Wahl and Nowak [16] in 2000, we have opted for the updated term “tolerance by synchronization”, which reflects the current terminology in microbiology for differentiating between the ability to grow despite sustained (“drug resistant”) versus transiently (“drug tolerant”) high drug levels. Here, tolerant strains survive repeated, transiently high drug levels via heritable life cycle timing. The main condition needed for emergence of tolerance by synchronization is tight control of viral life cycle timing. This tight control (i.e. low variance) may be achieved even if each life cycle stage has random duration, as long as there are sufficiently many stages.

In order for viral synchronization to be a feasible mechanism for drug tolerance, the life cycle length of wild-type virus must be at least close in magnitude to the dose interval (or an integer multiple thereof) since it may be unrealistic to assume that a virus could dramatically change its life cycle length. To this end, we examined the viral generation time—defined as the average time from the moment one cell is newly infected until one of its offspring infects a new cell—and the recommended dosing schedules for viral infections for which targeted therapy is available (Table S1 Table). This includes HIV, hepatitis B virus, hepatitis C virus, herpes simplex virus 1 and 2, cytomegalovirus, and influenzavirus A and B. We found that in all cases, the conditions for possible synchronization were met. Although current experimental methods of characterizing resistance generally preclude identification of synchronization-based mechanisms (detailed below), there are some hints of effects in which synchronization may play a role. For example, for HIV it is known that resistance mutations to protease inhibitors can occur outside the protease gene (e.g. [44]), and for hepatitis C virus, there are many examples of clinical failure without a known resistance mutation, or multi-drug resistant strains with no known mechanism (e.g. [45] and references therein.)

The “resistance” mechanism described here is aptly “cryptic” in the sense that it would evade detection by all existing *in vitro* tests for drug susceptibility. Genotypic resistance tests typically look for amino acid changes in the viral sequence which codes for the protein targeted by the drug, and particularly regions important for drug binding. Since a gene influencing viral life cycle length could appear anywhere in the genome, such tests would likely miss these mutations. In order to adapt these tests, experiments would need to be done to identify genetic loci associated with life cycle length and then adapt resistance screens to look for changes at these sites. Phenotypic resistance tests suffer a similar problem: they measure *in vitro* viral replication against a series of drug levels, but since these levels will be constant within the culture media, synchronization cannot occur and strains conferring tolerance will appear fully susceptible. These tests would need to be conducted in a device, such as a bioreactor or microfluidic chip, that could recreate drug profiles experienced *in vivo* to be able to detect tolerance via synchronization.

While we have shown that tolerance via life cycle synchronization is a possible means of evading therapy *in silico*, to our knowledge this effect has never been evaluated experimentally. A first step would be to create an *in vitro* viral culture system that could deliver periodic drug concentrations, and to find a model viral infection system in which genetic determinants of life cycle control are already established. Additionally, we would like to look for evidence of this strategy emerging in patients on antiviral therapy. A first step could be to identify patients who have only partially suppressed viremia despite high adherence and who have virus that appears drug-sensitive by the genotypic and/or phenotypic resistance tests described above.

Although this work is the first we are aware of to explore the evolution of viral life cycles in response to drug treatment, previous work has explored other determinants of life cycle length evolution, in particular for bacteriophages [17–24]. A classic question has been how long a phage should wait before lysing a host cell, when there is naturally a trade-off between benefit of delaying lysis to accumulate viral progeny within the cell, and the need to rapidly spread to other potential target cells. Models of this process have characterized the determinants of lysis time in terms of, for example, host cell density and intracellular host resources [18, 19], but have not, to our knowledge, considered periodic effects. We have incorporated ideas from this work into our model, showing that drug dosing period acts in combination with trade-offs between slowing maturation (to produce more progeny) and increasing maturation (to either spread faster or avoid death) to determine the optimal life cycle length.

The emergence of tolerance by viral life cycle synchronization can only lead to therapy failure, on its own, if trough drug levels are not fully suppressive. Trough levels depend on drug dose and the kinetics of absorption and clearance (e.g. *C*_max_ and *t*_*h*_), and suppression at trough levels depends on the viral fitness (*R*_0_) in the absence of drug, and the parameters of the dose-response curve (Eq (7)). For drugs with a short half-life, a steep dose-response curve, and a narrow therapeutic window, it is more likely that worries about toxicity prevent reaching *C*_max_ levels high enough to ensure that even trough concentrations prevent viral replication. Cryptic resistance is most likely to occur in this regime. Even if viral replication is suppressed for the wild-type strain throughout the dose interval, synchronization could augment partially-resistant mutations that act by standard mechanisms (e.g. altering drug binding).

Here, we have assumed that the viral infectivity is instantaneously affected by the current drug level. In reality, some drugs may need to undergo further steps, such as breakdown into active forms, active transport into cells, etc., which could delay their effect. These processes may alter the form of the periodic drug levels, but the periodic nature—and hence the potential for viral synchronization—will be preserved. However, if the drug binds irreversibly to a cellular target that does not turnover or intracellularly to a viral target that is not continually produced, or does so reversibly but with a very slow dissociation rate, then there may be no periodicity in drug effect despite a periodicity in concentrations.

We assumed that the drug acts on the infection of new target cells by free virus. However, the drug could also act on another phase of the life cycle, which could be represented as blocking the transition from one stage of immature infected cell to another. Tolerance could potentially occur in these scenarios as well, since the main requirement for its existence is for synchronized strains to possess an evolutionary advantage over the others. We saw that, in general, this is achieved through a tight control over the viral life cycle length.

For some viral infections, treatment is administered as combinations of different drugs, often with the same dosing schedules, that act on different stages of the virus life cycle. In this case it is much harder for synchronization to confer a benefit, as it may be impossible for the virus to complete the multiple targeted stages of its life cycle at the time when both drug levels are low. However, therapy failure could occur by a combination of resistance mechanisms—altered drug binding for one drug, and synchronization for the remaining drug.

In some viral infections, multiple infections of the same target cell may be common. When infection contains multiple viral strains, complex dynamics within multiply-infected cells can alter evolutionary dynamics. For example, recombination, by a variety of mechanisms, can occur and can have complex effects on selection [46]. Phenotypic mixing or multiplicity reactivation can lead to production of virions with mismatches between genotype (nucleic acid carried) and phenotype (structural and functional proteins carried), which also greatly complicates evolutionary predictions [47, 48]. Finally, within-cell competition can select for different traits than between-cell competition, for example leading to competitor colonizer trade-offs [49]. Future models, designed to more precisely capture the details of particular viral infections, could include multiply-infected cells.

Our results show that common methods for calculating *R*_0_ have limited use when considering periodically administered therapies. Even though a version of *R*_0_ can easily be constructed which takes into account the time-averaged viral fitness in the presence of fluctuating drug levels, this quantity does not discriminate between strains that can persist versus go extinct in the presence of the drug. This is because the process of synchronization does not just apply to strains that have life cycle lengths very close to integer multiples of the drug period, and hence benefit most from the effect. It also alters the long-term fitness of all strains. Strains that are most asynchronous do worse than predicted by the time-averaged *R*_0_. This failure of existing methods for *R*_0_ is due to the combined presence of both time-dependent effects and a stage-structured model (e.g. immature versus mature cells). We have derived a method for calculating a version of *R*_0_ that does account for synchronization, and although there appears to be no simple expression for this quantity, it can be calculated numerically and can predict which strains will persist under periodic drug levels.

The system we analyze in this paper has parallels to population-level epidemic models in which there may be periodic fluctuations in disease parameters. While the most common source of periodicity is seasonality [50], which likely occurs on a timescale much longer than the generation time of infection, other periods, such as weekly changes in contact rates due to work/school days versus weekends, may occur on timescales for which interactions with the generation time are more relevant. In fact, mathematicians studying such models have independently suggested constructs for the basic reproductive ratio under periodic model coefficients [51], proved that their definitions represent persistence thresholds [52], suggested algorithms to actually compute these quantities [53], and determined the scenarios under which the simpler time-averaged approach is correct [53]. They have even observed a phenomenon they call “resonance” [54], in which the early growth rate of such models is enhanced if the period of environmental change is close to some natural timescale of the infection, which is analogous to the effect we observe and call synchronization.

While we have discussed tolerance via synchronization in the context of viral infections, this is by no means the only possible case. Other microbial causes of infection, such as bacteria and protozoans, could also use this mechanism to avoid life-cycle-stage-specific drug targeting, assuming their life cycle length is greater than or equal to the drug period. Similarly, cancer chemotherapy may be administered with dose intervals in the right range for cell cycle synchronization to occur. These cellular organisms, as opposed to viruses, may not actually have to evolve synchrony via genetic changes, but may have the cellular machinery to make them capable of regulating cell-cycle length phenotypically.

In fact, in particular laboratory protocols used to grow and evolve bacterial cultures in the presence of antibiotics, an effect called “tolerance by lag” has been observed which bears some similarities to the viral life cycle synchronization that we describe here. In the context of antibiotic resistance, “tolerance” is defined as the ability to temporarily survive high levels of bacteriocidal antibiotic, and can occur by either genetic or non-genetic mechanisms. When bacteria are moved from a stressful environment where growth has been suppressed (for example, crowded culture media), to a resource-rich environment (e.g., diluted into new media), there is a well-documented “lag” phase before cell growth and division resumes. If cultured bacteria are repeatedly allowed to grow until stasis-via-overcrowding before being diluted and transferred, and if the transfer always involves a transient period of (bacteriocidal) antibiotic-treated media, then the population will evolve an altered lag time which matches with the length of antibiotic exposure [25–27]. While this work suggests bacteria too could use synchrony as a resistance mechanism, it remains unclear whether the *in vitro* growth protocol designed to force a lag phase at the time of treatment is relevant to any process that naturally occurs during infection.

## Supporting information

S1 Text Supplementary methods. (PDF)

S1 Table Life cycle data and example drug treatments for common viruses. (TIFF)

S1 Fig Time-course of viral fitness under pharmacologic model of periodic drug treatment. Fitness of the viral strain (measured by the basic reproductive ratio *R*_0_) fluctuates in response to drug levels approximated by the simple pharmacological model, Eq (7). **(A)** Perfect adherence to treatment. **(B)** Imperfect adherence to treatment: each dose is missed with a 30% probability. The model parameters used are drug period *T* = 2 days, drug-free *R*_0_ = 10, drug *IC*_50_ = 0.05 and slope (*M*) = 1.0, maximal drug level *C*_max_ = 8.0, minimum drug level when all doses are taken *C*_min_ = 0.04, dose size Δ = *C*_max_ − *C*_min_ = 7.96, and drug half-life *t*_*h*_ chosen such that the time-averaged drug efficacy is *f* = 0.85 (*t*_*h*_ = 0.25 days).
(TIF)

S2 Fig Equilibrium infection level for the single-strain deterministic model, as a function of the average maturation time. Viral dynamics were simulated under periodic antiviral therapy given by the simple on-off model with a period (*T*) of 2 days and varying drug efficacy (*f*). The infection level (heat map color) is measured as the concentration of mature infected cells (*y*) once a steady-state has been reached. Each calculation included only a single virus strain with average maturation time 1/*m* (maturation rate of *nm* for each stage). **(A)** Results with *n* = 1 maturation step. **(B)** Results with *n* = 10 maturation steps. **(C)** Results with *n* = 25 maturation steps. **(D)** Results with fixed maturation time *τ* = 1/*m*. The white dotted lines show where the average maturation time is equal to an integer multiple of the drug period. For all shown simulations, we assume the death rate of immature cells to be zero (*d*_*w*_ = 0). Results shown for 41 different drug efficacies between *f* = 0.85 and *f* = 0.95, for 41 different strains with average maturation times between 1 and 6 days.
(TIF)

S3 Fig Equilibrium infection level for the single-strain deterministic model, as a function of the average maturation time, when the infected cells can die before maturing. Viral dynamics were simulated under periodic antiviral therapy given by the simple on-off model with a period (*T*) of 2 days and varying drug efficacy (*f*). The infection level (heat map color) is measured as the concentration of mature infected cells (*y*) once a steady-state has been reached. Each calculation included only a single virus strain with average maturation time 1/*m* (maturation rate of *nm* for each stage). **(A)** Results with *n* = 1 maturation step. **(B)** Results with *n* = 10 maturation steps. **(c)** Results with *n* = 25 maturation steps. **(d)** Results with fixed maturation time *τ* = 1/*m*. For all shown simulations, we set the death rate of immature cells to *d*_*w*_ = 0.1. Data shown for 41 different drug efficacies between *f* = 0.6 and *f* = 1.0, for 101 different strains with average maturation times between 1 and 6 days.
(TIF)

S4 Fig Stochastic competition between viral strains with different life cycle times, for the entire range of drug efficacies. Viral dynamics were simulated under periodic antiviral therapy given by the simple on-off model with a period (*T*) of 2 days and varying drug efficacy (*f*). The fixation probability (heat map color) is measured as the fraction of simulations in which a strain was the last surviving in the population and continued on to reach a steady state. **(A)** Results with *n* = 10 maturation steps. **(B)** Results with fixed maturation time *τ* = 1/*m*. For all shown simulations, we assume the death rate of immature cells to be zero (*d*_*w*_ = 0). 100 simulations were run for each drug efficacy level. Data shown for 11 different values of the drug efficacy between *f* = 0.0 and *f* = 1.0, for competitions between 51 different strains with average maturation times between 1 and 6 days.
(TIF)

S5 Fig Stochastic competition between viral strains with different life cycle times, when infected cells can die before maturing. Viral dynamics were simulated under periodic antiviral therapy given by the simple on-off model with a period (*T*) of 2 days and varying drug efficacy (*f*). The fixation probability (heat map color) is measured as the fraction of simulations in which a strain was the last surviving in the population and continued on to reach a steady state. **(A)** Results with *n* = 10 maturation steps. **(B)** Results with fixed maturation time *τ* = 1/*m*. For all shown simulations, we assume the death rate of immature cells to be *d*_*w*_ = 0.1. 100 simulations were run for each drug efficacy level. Data shown for 11 different values of the drug efficacy between *f* = 0.0 and *f* = 1.0, for competitions between 51 different strains with average maturation times between 1 and 6 days.
(TIF)

S6 Fig Average equilibrium infection level for the single-strain competition deterministic model with pharmacological drug kinetics, as a function of the average maturation time (1/*m*). The infection level (heat map color) is measured as the concentration of mature infected cells (*y*) once a steady-state has been reached. Each calculation included only a single virus strain with average maturation time 1/*m* (maturation rate of *nm* for each stage). **(A)** Results with *n* = 1 maturation step. **(B)** Results with *n* = 10 maturation steps. **(C)** Results with *n* = 25 maturation steps. **(D)** Results with fixed maturation time *τ* = 1/*m*. The white dotted lines show where the average maturation time is equal to an integer multiple of the drug period. For all shown simulations, we assume the death rate of immature cells to be zero (*d*_*w*_ = 0). Results shown for 41 different drug efficacies between *f* = 0.6 and *f* = 1.0, for 101 different strains with average maturation times between 1 and 6 days.
(TIF)

S7 Fig Average equilibrium infection level for the multi-strain competition deterministic model with pharmacological drug kinetics, as a function of the average maturation time (1/*m*). The infection level is measured as the concentration of mature infected cells *y*. **(a)** Results of deterministic model with *n* = 10 maturation steps. **(b)** Results of deterministic model with fixed maturation time *τ* = 1/*m*. The model parameters used are period *T* = 2 days, *IC*_50_ = 0.05, *M* = 1.0, *C*_max_ = 8.0, and *τ* chosen such that the time-averaged drug efficacy is *f* = 0.85. For all shown simulations, we assume the death rate of immature cells to be zero (*d*_*w*_ = 0). Results shown for 501, 501, and 108 different strains, for panels from **A** to **C** respectively, with average maturation times between 1 and 6 days.
(TIF)

S8 Fig Average equilibrium infection level for the multi-strain competition deterministic model with imperfect therapy adherence and on/off drug kinetics, as a function of the average maturation time (1/*m*). The infection level is measured as the concentration of mature infected cells *y*, under anti-viral therapy drug efficacy *f* = 0.85 and period *T* = 2 days. **(A)** Results of deterministic model with *n* = 10 maturation steps. **(B)** Results of deterministic model with fixed maturation time *τ* = 1/*m*. For all shown simulations, we assume the death rate of immature cells to be zero (*d*_*w*_ = 0). The adherence level is the probability that any scheduled dose is taken. We assume that each dose is taken independently. Results shown for 501 and 108 different strains, for panels **A** and **B**, with average maturation times between 1 and 6 days.
(TIF)

S9 Fig Average equilibrium infection level for the multi-strain competition deterministic model with on/off drug kinetics, when the drug dosage times are normally distributed, as a function of the average maturation time (1/*m*). The infection level is measured as the concentration of mature infected cells *y*, under anti-viral therapy with drug efficacy *f* = 0.85 and period *T* = 2 days. **(A)** Results of deterministic model with *n* = 10 maturation steps. **(B)** Results of deterministic model with fixed maturation time *τ* = 1/*m*. For all shown simulations, we assume the death rate of immature cells to be zero (*d*_*w*_ = 0). The drug dosage times are drawn from a normal (Gaussian) distribution with mean *μ* = 2 days and standard deviation *σ* between 36 minutes and 6 hours. Results shown for 501 and 101 different strains, for panels **A** and **B**, with average maturation times between 1 and 6 days.
(TIF)

S10 Fig Competition between viral strains with different life cycle times when there are trade-offs in maturation time. We consider a model in which there is a trade-off to the time spent in the immature phase, even in the absence of drug. The longer the time spent in this phase, the higher the eventual viral burst size and infectivity, but the more chance of dying without maturing. **(A)**The relationship between viral infectivity *β* (scaled by *β*_0_) and maturation time *τ* under the model of precursor production during maturation in the absence of drug. **(B)**The relationship between net viral fitness *R*_0_ and maturation time *τ* in the absence of drug. We scale the precursor production rate *α* so that the maximum *R*_0_ is constant across precursor decay rates. For both panels we use *δ* = 1.3, 0.7, or 0.01/day, *d*_*w*_ = 0.1/day. **(C-E)** Equilibrium infection level for the multi-strain deterministic model under periodic drug treatment when there are trade-offs in life cycle length. The infection level (y-axis) is measured as the concentration of mature infected cells (*y*) once a steady-state has been reached. Each simulation included a collection of viral strains with the full range of fixed maturation times (*τ*) shown. Drug dynamics were given by the simple on-off model with a drug efficacy of 85% (*f* = 0.85). **(C)** Precursor decay rate *δ* = 1.3/day. **(D)** Precursor decay rate *δ* = 0.7/day. **(E)** Precursor decay rate *δ* = 0.01/day. For all shown simulations, we assume the death rate of immature cells to be *d*_*w*_ = 0.1.
(TIF)

S11 Fig Maturation time of optimal strains across multiple models. The optimal strain is defined as the one with the highest time-average equilibrium level of mature infected cells (y), either when simulated alone (single strain models) or in competition with other strains (multi-strain model). In all cases we only looked at strains with maturation times less than or equal to the drug period *T* = 2 days. When *d*_*y*_ was altered from 1, *β* was increased by the same amount to keep *R*_0_ in the absence of drug the same. For all shown simulations, we assume the death rate of immature cells to be zero (*d*_*w*_ = 0). The grey line describes the curve *m* = *T* − *T*(1 − *f*)/2. The total viral generation time is the sum of the maturation time (x-axis) and the average time between a cell maturing and a secondary infection occurring. When the average lifespan of a mature infected cell (1/*d*_*y*_) exceeds the time the drug is off (*T*(1 − *f*)) in the simple on-off model, then the average time until secondary infection is *T*(1 − *f*)/2), so the optimal maturation time is expected to be lower than the drug period by this amount. This prediction only works when *d*_*y*_ is small, maturation time is tightly controlled, drug displays simple on-off kinetics, and drug efficacy is relatively high.
(TIF)

S12 FigStream plot of the vector fields $\dot{y}$ and $\dot{x}$ for the infection level *y* and the healthy cells level *x* in the single-strain model with *n* = 2 maturation steps, and constant drug level.**(A)** Stability of no-infection steady state when *R*_0_ < 1. **(B)** Stability of infection steady state when *R*_0_ > 1. The corresponding equations are shown in Eq (1) and Eq. S.1. The red point represents the equilibrium point for the system. For all shown results, we set the death rate of immature cells to *d*_*w*_ = 0.1, and we assume that the concentration of healthy cells *x* and the concentration of immature infected cells *w* are close to steady state.(TIF)

S13 FigStream plot of the vector fields ${\dot{y}}_{1,2}$ and $\dot{x}$ for the infection levels *y*_1,2_ in the 2-strain (*N* = 2) competition model with constant drug level.**(A)** Stability of no-infection steady state when *R*_0_ < 1 for both viral strains. **(B)** Stability of infection steady state when *R*_0_ > 1 for both viral strains. The corresponding equations are shown in Eq. S.3. The red point represents the equilibrium point for the system. For all shown results, we assume the death rate of immature cells to be zero (*d*_*w*_ = 0), and that the concentration of healthy cells *x* and immature infected cells *w*_1,2_ are close to steady state.(TIF)

S14 FigStream plot of the vector fields ${\dot{y}}_{1,2}$ and $\dot{x}$ for the infection levels *y*_1,2_ in the 2-strain (*N* = 2) competition model with constant drug level.**(A)** Stability of no-infection steady state when *R*_0_ < 1 for both viral strains and *m*_1_ > *m*_2_. **(B)** Stability of infection steady state when the basic reproductive ratio *R*_0_ > 1 for both viral strains and *m*_1_ > *m*_2_. **(C)** Stability of infection steady state when the basic reproductive ratio *R*_0_ > 1 for both viral strains and *m*_1_ = *m*_2_. The corresponding equations are shown in Eq. S.3. The red point represents the equilibrium point for the system. For all shown results, we set the death rate of immature cells to *d*_*w*_ = 0.1, and we assume that the concentration of healthy cells *x* and immature infected cells *w*_1,2_ are close to steady state.(TIF)

S15 FigTime course of early infection dynamics and calculated growth rate, ${\tilde{r}}_{0}$.Infection dynamics with the fixed-delay model (Eq (3)) were numerically integrated starting from a small amount of infection introduced to the uninfected equilibrium. The level of mature infected cells is shown (blue line). To estimate ${\tilde{r}}_{0}$ from these dynamics, we took points sampled at intervals of *T* and fit to an exponential curve (red line). We compared this to the value of ${\tilde{r}}_{0}$ predicted from our analytic work (Eq. S.99). a) Maturation time of *τ* = 0.4: infection decreases with drug. The observed value of ${\tilde{r}}_{0}$ (−0.324) matches excellently with the predicted value (−0.324). b) Maturation time of *τ* = 1.6: infection grows despite drug. The observed value of ${\tilde{r}}_{0}$ (0.123) matches excellently with the predicted value (0.123). We used parameters from Table 1 along with *d*_*w*_ = 0.1 and *f* = 0.9. Initial values were *x*(0) = 10^3^ and *y*(0) = 10^−6^. Fits were conducted using points between times of 10 and 30 days using the method of least squares.(TIF)
