# Supplementary material for: Life cycle synchronization is a viral drug resistance mechanism
Source: PLoS Comput Biol. 2018 Feb 15;14(2):e1005947. doi: 10.1371/journal.pcbi.1005947 (PMC5813899; doi:10.1371/journal.pcbi.1005947)
Supplement: S1 Text — (PDF) [file pcbi.1005947.s001.pdf]

SUPPLEMENTARY METHODS

Life Cycle Synchronization is a Viral Drug Resistance Mechanism

Iulia A. Neagu[1,2], Jason Olejarz[1], Mark Freeman[1], Daniel I. S. Rosenbloom[3], Martin A. Nowak[1], and Alison L. Hill[1,*]

[1]Program for Evolutionary Dynamics, Department of Mathematics, Department of Organismic and Evolutionary Biology, Harvard University, Cambridge, MA, USA
[2]Department of Physics, Harvard University, Cambridge, MA, USA
[3]Department of Biomedical Informatics, Columbia University Medical Center, New York, NY, USA
[*] alhill@fas.harvard.edu

Contents

1 Analytic results for single-strain model with constant drug level 3
1.1 Maturation happens in n intermediate steps 3
1.2 Maturation happens after a fixed time delay 3

2 Analytic results for multi-strain model with constant drug levels 4
2.1 Maturation happens in n intermediate steps 4
2.2 Maturation happens after a fixed time delay 5

3 Initial conditions 6

4 Equilibrium conditions 7

5 Alternative model with costs to viral life cycle length 7

6 Derivation of basic reproductive ratio with periodic drug levels 9
6.1 The maturation process happens with no intermediate maturation steps 9
6.2 The maturation process happens in n intermediate maturation steps 13
6.3 The maturation process happens after a fixed time delay 26

1 Analytic results for single-strain model with constant drug level

1.1 Maturation happens in n intermediate steps

Consider the single-strain viral dynamics model with n maturation steps (Eq. (1)) where $m_i = m_n$ for all i. For a constant infection efficacy β, this system of equations has a unique steady state where infection does not occur, for which $x^* = \lambda/d_x$, $y = w_i = 0$, $\forall i \leq n$. This steady state is stable if $R_0 < 1$, where R_0 is the basic reproductive ratio for the multi-stage model as defined in Eq.(4).

The system also has a unique stable steady state where infection occurs (see S12 Fig) when $R_0 > 1$, such that:

$$\begin{aligned}
x^* &= \frac{d_y}{\beta}\left(\frac{nm + d_w}{nm}\right)^n \\
&= \frac{\lambda}{d_x R_0} \\
y^* &= \frac{\lambda}{d_y}\left(\frac{nm}{nm + d_w}\right)^n - \frac{d_x}{\beta} \\
&= \frac{\lambda}{d_y}\left(\frac{nm}{nm + d_w}\right)^n \left(1 - \frac{1}{R_0}\right) \\
w_i^* &= \frac{\lambda}{nm}\left(\frac{nm}{nm + d_w}\right)^i - \frac{d_x d_y}{\beta(nm + d_w)}\left(\frac{nm + d_w}{nm}\right)^{n-i+1} \\
&= \frac{\lambda}{nm}\left(\frac{nm}{nm + d_w}\right)^i \left(1 - \frac{1}{R_0}\right), \ i = 1 \ldots n
\end{aligned} \tag{S.1}$$

1.2 Maturation happens after a fixed time delay

Consider the single-strain viral dynamics model with fixed maturation time $\tau = 1/m$ (Eq.(3)). For a constant infection efficacy β, this system of equations has a unique steady state where infection does not occur, for which $x^* = \lambda/d_x$, $y = 0$. This steady state is stable if $R_0 < 1$, where R_0 is the basic reproductive ratio for the fixed-time model as defined in Eq.(5).

The system also has a unique stable steady state where infection occurs when $R_0 > 1$, such that:

$$\begin{aligned}
x^* &= \frac{d_y}{\beta} e^{d_w \tau} \\
&= \frac{\lambda}{d_x R_0} \\
y^* &= \frac{\lambda}{d_y} e^{-d_w \tau} - \frac{d_x}{\beta} \\
&= \frac{\lambda}{d_y} e^{-d_w \tau}\left(1 - \frac{1}{R_0}\right)
\end{aligned} \tag{S.2}$$

2 Analytic results for multi-strain model with constant drug levels

2.1 Maturation happens in n intermediate steps

We used the following system to model competition between N viral strains with different maturation times that co-infect the same host, when the maturation process happens in n steps:

$$\begin{aligned}
\dot{x} &= \lambda - \beta x \sum_{k=1}^{N} y_k - d_x x \\
\dot{w}_{k1} &= \beta x y_k - (n m_k + d_w) w_{k1} \\
\dot{w}_{ki} &= n m_k w_{k,i-1} - (n m_k + d_w) w_{ki} \quad i = 2 \dots n \\
\dot{y}_k &= m_k w_{kn} - d_y y_k
\end{aligned} \tag{S.3}$$

Here i is the index of the maturation phase ($1 \leq i \leq n$) and k is the index of the strain ($1 \leq k \leq N$). For a constant infectivity β, this model has a unique steady state where infection does not occur, for which $x^* = \lambda/d_x$, $y_k^* = w_{ki}^* = 0$, $\forall\ i \leq n, \forall\ k \leq N$. This steady state is stable if $R_k < 1$, $\forall\ k = 1 \dots N$.

When $d_w = 0$, the system has an infinite number of stable steady-states where infection occurs (see S13 Fig), such that:

$$\begin{aligned}
x^* &= \frac{d_y}{\beta} \\
\sum_{k=1}^{N} y_k^* &= \frac{\lambda}{d_y} - \frac{d_x}{\beta} \\
w_{ki}^* &= w_{kj}^* \equiv w_k^*,\ \forall i,\ j = 1 \dots n \\
n \sum_{k=1}^{N} m_k w_k^* &= \lambda - \frac{d_x d_y}{\beta}
\end{aligned} \tag{S.4}$$

Similarly, when $d_w > 0$, the system has N steady states where infection occurs, for which:

$$\begin{aligned}
x^* &= \frac{d_y}{\beta} \left(\frac{n m_k + d_w}{n m_k} \right)^n \\
&= \frac{\lambda}{d_x R_k} \\
y_k^* &= \frac{\lambda}{d_y} \left(\frac{n m_k}{n m_k + d_w} \right)^n - \frac{d_x}{\beta} \\
&= \frac{\lambda}{d_y} \left(\frac{n m_k}{n m_k + d_w} \right)^n \left(1 - \frac{1}{R_k} \right) \\
w_{ki}^* &= \frac{\lambda}{n m_k} \left(\frac{n m_k}{n m_k + d_w} \right)^i - \frac{d_x d_y}{\beta (n m_k + d_w)} \left(\frac{n m_k + d_w}{n m_k} \right)^{n-i+1} \\
&= \frac{\lambda}{n m_k} \left(\frac{n m_k}{n m_k + d_w} \right)^i \left(1 - \frac{1}{R_k} \right) \text{ for a unique strain } k \\
w_{ji}^* &= y_j^* = 0,\ \forall m_j \neq m_k, i = 1 \dots n, j = 1 \dots N
\end{aligned} \tag{S.5}$$

where

$$R_k = \frac{\lambda\beta}{d_x d_y}\left(\frac{nm_k}{nm_k + d_w}\right)^n \tag{S.6}$$

is the basic reproductive ratio of the strain with maturation rate m_k.

R_k is a monotonically increasing function of m_k for $d_w \neq 0$, and does not depend on m_k when the death rate d_w of immature infected cells is zero ($d_w = 0$). The same thing can be said about the steady-state value of mature infected cells y_k, while the steady-state value of immature infected cells is inversely related to the maturation rate m_k for all d_w.

The equilibrium described by Eq. (S.5) implies competitive exclusion: only one strain survives at equilibrium and all others go extinct. Only 1 out of N of the possible equilibria are stable, and this is the one in which the strain with the highest maturation rate and thus the highest R_k survives. All other infected equilibria are unstable (see S14 Fig).

2.2 Maturation happens after a fixed time delay

We used the following system to model competition between N viral strains with different fixed maturation times $\tau_k = 1/m_k,\ k = 1 \ldots N$, that co-infect the same host:

$$\begin{aligned} \dot{x} &= \lambda - \beta x \sum_{k=1}^{N} y_k - d_x x \\ \dot{y}_k &= \beta x(t-\tau_k) y_k(t-\tau_k) e^{-d_w \tau_k} - d_y y_k,\ k = 1 \ldots N \end{aligned} \tag{S.7}$$

For a constant infectivity β, the multi-strain competition model with fixed maturation times has a unique steady state where infection does not occur, for which $x^* = \lambda/d_x,\ y_k^* = 0,\ \forall k \leq N$. This steady state is stable if $R_k < 1,\ \forall k = 1 \ldots N$, where R_k is the basic reproductive ratio for the model with fixed maturation time as defined in Eq. (5).

When $d_w = 0$, the system has an infinite number of stable steady-states where infection occurs such that:

$$\begin{aligned} x^* &= \frac{d_y}{\beta} \\ \sum_{k=1}^{N} y_k^* &= \frac{\lambda}{d_y} - \frac{d_x}{\beta} \end{aligned} \tag{S.8}$$

When $d_w > 0$, the system has N steady states where infection occurs, for which:

$$\begin{aligned} x^* &= \frac{d_y}{\beta} e^{d_w \tau_k} \\ &= \frac{\lambda}{d_x R_k} \\ y_k^* &= \frac{\lambda}{d_y} e^{-d_w \tau_k} - \frac{d_x}{\beta} \\ &= \frac{d_x}{\beta}(R_k - 1),\ \text{for a unique strain } k \\ y_j^* &= 0,\ \forall \tau_j \neq \tau_k, j = 1 \ldots N \end{aligned} \tag{S.9}$$

Similarly to Eq. (S.5), the only stable equilibrium resulting from Eq. (S.9) is the one where only the strain with the shortest maturation time (highest R_k) survives. Therefore at steady state, $y_j^* = 0$, where $\tau_k < \tau_j,\ \forall j \neq k, j = 1 \ldots N$. All other infected equilibria are unstable.

3 Initial conditions

The initial conditions for our analysis correspond to the levels of uninfected cells and infected cells of different life cycle stages present *before* drug treatment is administered. For the single-strain models, we use as initial conditions the infected equilibrium of the model in the absence of drug (i.e. when the drug efficacy $f = 0$). Explicitly, the initial condition for the single-strain model with n maturation steps is calculated using Eq. (S.1). For the single-strain model with fixed maturation time, the initial condition ($x(t \leq 0)$ and $y(t \leq 0)$) is calculated using Eq. (S.2).

For the multi-strain competition models (with N strains), the situation is more complicated, because either there exists an infinite number of possible equilibria containing all strains ($d_w = 0$) or no equilibrium including all strains ($d_w > 0$). Therefore, we had to either choose or create an initial condition which contained a balanced representation of each strain.

For the multi-strain competition model with n maturation steps, we set the following initial conditions:

$$
\begin{aligned}
x^* &= \frac{1}{N}\sum_{k=1}^{N}\frac{d_y}{\beta}\left(\frac{nm_k + d_w}{nm_k}\right)^n \\
y_k^* &= \frac{1}{N}\left[\frac{\lambda}{d_y}\left(\frac{nm_k}{nm_k + d_w}\right)^n - \frac{d_x}{\beta}\right] \\
w_{ki}^* &= \frac{1}{N}\left[\frac{\lambda}{nm_k}\left(\frac{nm_k}{d_w + nm_k}\right)^i - \frac{d_x d_y}{\beta(nm_k + d_w)}\left(\frac{nm_k + d_w}{nm_k}\right)^{n-i+1}\right] \\
& i = 1 \ldots n,\ k = 1 \ldots N
\end{aligned} \tag{S.10}
$$

For the multi-strain competition model with fixed maturation times, we set the following initial conditions (for all pre-treatment time points):

$$
\begin{aligned}
x^* &= \frac{1}{N}\sum_{k=1}^{N}\frac{d_y}{\beta}e^{d_w \tau_k} \\
y_k^* &= \frac{1}{N}\left(\frac{\lambda}{d_y}e^{-d_w \tau_k} - \frac{d_x}{\beta}\right),\ k = 1 \ldots N
\end{aligned} \tag{S.11}
$$

These initial conditions were set for both the deterministic and stochastic multistrain models; for the stochastic implementation, the cell concentrations were rounded to the nearest integer.

When $d_w = 0$, these expressions represent one of the (infinite) possible no-drug equilibria (Eqs. (S.4) and(S.8)). When $d_w > 0$, the only stable no-drug equilibria are ones where only a single strain persists (Eqs. (S.5) and(S.9)), but here, we construct a distribution of all N strains such that each one exists at $1/N$ of the level it would exist at if it were the only strain in the population.

4 Equilibrium conditions

Both the single-strain and multi-strain deterministic simulations were terminated when all strains had a relative change of less than 5% over consecutive 10 day periods in each cell type (healthy, infected-mature, infected-immature).

The stochastic competition simulation was terminated when a single strain survived in the population, and the relative change in each cell type (healthy, infected-mature, infected-immature) was less that 0.5% over consecutive 5 day periods. These conditions also allow for no strains to fix in the population.

Plots of the cell concentrations as a function of time were used to verify that the above thresholds were necessary and sufficient in identifying the equilibrium state of the simulated systems.

5 Alternative model with costs to viral life cycle length

Throughout this paper we have assumed that time spent by infected cells in immature phases is either cost-less or costly, depending on the death rate of immature infected cells (d_w). However, in reality there may be a benefit to longer time spent in the immature phases, because it is during these phases that the precursor compounds used to construct new viral particles are produced, and hence maturation time may be positively correlated with the eventual viral burst size and infectivity. Here we present a simple model to take these effects into account.

Consider the viral dynamics model with fixed maturation time (Eqs. (3)). Assume that immature infected cells produce a precursor molecule (concentration defined by state variable P), that is needed for infectious virions to be produced when the cell advances to a mature phase. Let's assume that P is produced in a single immature infected cell at rate α, and that it decays at a per capita rate δ. Therefore, in a given immature infected cell, P follows

$$\dot{P} = \alpha - \delta P$$

Since an infected cell only stays in the immature phase for time τ, this equation only holds for $t \in (0, \tau)$. We assume that there is initially no precursor molecule, so $P(0) = 0$. Therefore, the concentration of P over time follows

$$P(t) = \frac{\alpha}{\delta}(1 - e^{-\delta t})$$

We want to be able to vary the production rate of P without varying the eventual steady-state level as $t \to \infty$, and we are not worried about the units of P, so we set $\alpha = \delta$ and have the final concentration of P when the cell becomes mature as

$$P(\tau) = (1 - e^{-\delta \tau})$$

Now we assume that the amount of this precursor molecule directly translates into the amount of virions that can be produced by mature infected cells, and therefore into the viral burst size k and net infectivity β. We then get that viral infectivity depends on the maturation time τ, even in the absence of fluctuating drug levels:

$$\beta(\tau) = \beta_0(1 - e^{-\delta \tau})$$

When drug levels are not constant, β would also depend on the time-dependent drug efficacy, $\epsilon(t)$, which is given by $D(t)$ in the on-off drug model (Eq.(6)), and $1/(1+(D(t)/IC_{50})^M)$ in the pharmacological model (Eq. (7)).

$$\beta(\tau, t) = \beta_0(1 - e^{-\delta\tau})(1 - \epsilon(t))$$

S10 Fig A shows the dependence of viral infectivity on maturation time under this model with constant drug levels. When maturation time τ is less than $1/\delta$, infectivity increases approximately linearly with maturation time, and so there is a clear cost to maturing faster. However when maturation time is much longer than $1/\delta$, infectivity is roughly constant with maturation time, so there is little cost to changing maturation time. Therefore this model can be used to go between situations where there is and where there is not a cost to speeding up maturation.

We can also examine how this cost to maturation time influences net fitness by considering the basic reproductive ratio (R_0). Including $\beta(\tau)$ in the expression for R_0 for the fixed maturation time model, we get

$$R_0 = \frac{\lambda\beta_0}{d_x d_y} e^{-d_w\tau}(1 - e^{-\delta\tau})$$

While allowing death of immature infected cells ($d_w > 0$) increases fitness of strains who mature faster, including production of the precursor *decreases* fitness of quickly maturing strains. In the presence of constant drug levels there is trade-off between these two effects. The optimal maturation time is

$$\tau_c = \frac{1}{\delta}\ln\left(1 + \frac{\delta}{d_w}\right) \tag{S.12}$$

We additionally want to ensure that $R_0(\tau_c)$ is independent of δ, so that δ only controls what the optimal maturation time would be in the absence of drug, but not what the viral fitness would be at this optimum. To do this, we need to choose a different value for the production rate α, and we instead use the following τ-dependent infectivity β

$$\beta(\tau, t) = \beta_0(1 - e^{-\delta\tau})\frac{(1+u)^{1+u}}{u^u}(1 - \epsilon(t))$$
$$u = \frac{d_w}{\delta}$$

and the time-offset version

$$\beta(\tau, t-\tau) = \beta_0(1 - e^{-\delta\tau})\frac{(1+u)^{1+u}}{u^u}(1 - \epsilon(t-\tau)).$$

With this scaling, the basic reproductive ratio in the absence of drug becomes:

$$R_0 = \frac{\lambda\beta_0}{d_x d_y} e^{-d_w\tau}(1 - e^{-\delta\tau})\frac{(1+u)^{1+u}}{u^u} \tag{S.13}$$

S10 Fig B shows the dependence R_0, Eq. (S.13) on maturation time for different parameter values. Keeping all other parameters the same as Table 1, we consider three different regimes based on the rate of precursor production and decay, δ

- $\delta = 1.3$: Drug-free optimum τ_c=3 days, near the optimal synchronized life cycle length of 2 days

- $\delta = 0.7$: Drug-free optimum τ_c=3 days, near the *least optimal* life cycle length

- $\delta = 0.01$: Drug-free optimum τ_c=9.5 days is greater than any of the simulated maturation times

6 Derivation of basic reproductive ratio with periodic drug levels

In this section, we analytically investigate the dynamics of a single-strain model of viral infection with periodic drug levels. First, we consider the case in which an infected cell matures and begins producing new infections immediately. The calculations for this case are simple and foster intuition on our method of analysis. Next, we consider the case in which a newly infected cell matures in n intermediate stages before producing new infections. Finally, we consider the case in which a newly infected cell matures and begins producing new infections after a fixed time delay, τ. The analytical methods in this section are based on Floquet theory [1, 2, 3, 4, 5, 6].

6.1 The maturation process happens with no intermediate maturation steps

It is instructive to first consider the case where $\beta(t) = \beta(t+T)$ is time-dependent and periodic, but there are no intermediate maturation steps, so that newly infected cells begin producing new virions immediately. This simpler case helps to foster intuition for the more intricate calculations that follow. The viral dynamics are specified by the following system:

$$\begin{aligned}\dot{x}(t) &= \lambda - \beta(t)x(t)y(t) - d_x x(t)\\ \dot{y}(t) &= \beta(t)x(t)y(t) - d_y y(t)\end{aligned}$$

We assume that the system starts from the uninfected equilibrium, and a small amount of the virus is introduced. If we focus on the early-time dynamics, when the density of infected cells is low, then we have

$$\dot{y}(t) = \frac{\lambda}{d_x}\beta(t)y(t) - d_y y(t)$$

This equation can be rewritten as

$$\frac{d}{dt}\left(e^{d_y t}y(t)\right) = \frac{\lambda}{d_x}\beta(t)e^{d_y t}y(t) \tag{S.14}$$

Our goal is to use Equation (S.14) to determine how $y(L)$ is related to $y(L+T)$. (Here, L is any early time in the dynamics such that the density of target cells is approximately at its uninfected value, so that Equation (S.14) holds.) There is a simple intuition for why we are interested in how $y(L)$ is related to $y(L+T)$: Depending on the values of the model parameters, when tracked over a long-enough time period, $y(t)$ will tend to either grow or decay, since the particular viral strain under consideration will either establish a persistent infection or go extinct. Moreover, $\beta(L) = \beta(L+T)$ is

periodic, and this periodicity of $\beta(t)$ influences the infection level, $y(t)$. Therefore, we expect that $y(t)$ responds to $\beta(t)$ in an irregularly periodic pattern. Indeed, $y(t)$ shows quasiperiodic behavior in response to $\beta(t)$. Specifically, $y(t)$ has the following form [1, 2]:

$$\begin{aligned} y(t) &= Y(t)e^{\tilde{r}_0 t} \\ Y(t) &= Y(t+T) \end{aligned} \tag{S.15}$$

So, if we determine how $y(L)$ is related to $y(L+T)$, then the periodic prefactor, $Y(L) = Y(L+T)$, cancels out of the analysis (for example, when computing $y(L+T)/y(L)$), and we are able to solve for $\tilde{r}_0$, which is key: If $\tilde{r}_0 > 0$, then, when sampled at times that are integer multiples of the drug-dosing period, the density of infected cells increases in time, and infection is established. If $\tilde{r}_0 < 0$, then, when sampled at times that are integer multiples of the drug-dosing period, the density of infected cells decreases in time, and infection is eliminated.

To find a connection between $y(L)$ and $y(L+T)$, we begin by integrating Equation (S.14) between time L and time $L + t_k$; we obtain

$$e^{d_y t_k} y(L+t_k) = y(L) + \frac{\lambda}{d_x} \int_0^{t_k} dt_{k-1}\, \beta(t_{k-1}) e^{d_y t_{k-1}} y(L+t_{k-1}) \tag{S.16}$$

(In Equation (S.16), similarly to L, t_k is any early time in the dynamics such that the density of infected cells is low, so that Equation (S.14) holds. t_{k-1} is just an integration variable, but the notation t_{k-1} for the integration variable is convenient, as it implies the manipulations that are to follow. The intuition behind the notation for the integration variables will become clearer in the following two sections. Also, for simplicity of notation, we assume that L is equal to an integer multiple of T, so that $\beta(L+t_{k-1}) = \beta(t_{k-1})$.)

We proceed by noting that Equation (S.16) can be repeatedly substituted into itself. To see how this works, we set $k = 0$ and $t_0 = T$ in Equation (S.16); we obtain

$$e^{d_y T} y(L+T) = y(L) + \frac{\lambda}{d_x} \int_0^{T} dt_{-1}\, \beta(t_{-1}) e^{d_y t_{-1}} y(L+t_{-1}) \tag{S.17}$$

Next, we use Equation (S.16) to substitute for $e^{d_y t_{-1}} y(L+t_{-1})$ in Equation (S.17); we obtain

$$\begin{aligned} e^{d_y T} y(L+T) &= y(L) + y(L)\frac{\lambda}{d_x} \int_0^{T} dt_{-1}\, \beta(t_{-1}) \\ &\quad + \left(\frac{\lambda}{d_x}\right)^2 \int_0^{T} dt_{-1}\, \beta(t_{-1}) \int_0^{t_{-1}} dt_{-2}\, \beta(t_{-2}) e^{d_y t_{-2}} y(L+t_{-2}) \end{aligned} \tag{S.18}$$

Continuing, we use Equation (S.16) to substitute for $e^{d_y t_{-2}} y(L+t_{-2})$ in Equation (S.18); we obtain

$$\begin{aligned} e^{d_y T} y(L+T) &= y(L) + y(L)\frac{\lambda}{d_x} \int_0^{T} dt_{-1}\, \beta(t_{-1}) \\ &\quad + y(L)\left(\frac{\lambda}{d_x}\right)^2 \int_0^{T} dt_{-1}\, \beta(t_{-1}) \int_0^{t_{-1}} dt_{-2}\, \beta(t_{-2}) \\ &\quad + \left(\frac{\lambda}{d_x}\right)^3 \int_0^{T} dt_{-1}\, \beta(t_{-1}) \int_0^{t_{-1}} dt_{-2}\, \beta(t_{-2}) \\ &\quad \times \int_0^{t_{-2}} dt_{-3}\, \beta(t_{-3}) e^{d_y t_{-3}} y(L+t_{-3}) \end{aligned} \tag{S.19}$$

Continuing further, we use Equation (S.16) to substitute for $e^{d_y t_{-3}} y(L + t_{-3})$ in Equation (S.19), then we use Equation (S.16) to substitute for $e^{d_y t_{-4}} y(L + t_{-4})$ in the resulting equation, etc.; continuing this process *ad infinitum*, we arrive at

$$y(L+T) = My(L) \tag{S.20}$$

where M is given by

$$M = e^{-d_y T} \left[1 + \sum_{j=1}^{\infty} \left(\frac{\lambda}{d_x} \right)^j \int_0^T dt_{-1}\, \beta(t_{-1}) \prod_{k=2}^{j} \int_0^{t_{-k+1}} dt_{-k}\, \beta(t_{-k}) \right] \tag{S.21}$$

(Throughout this Supplement, we define the empty product, as for $j = 1$ in the product in Equation (S.21), as being equal to 1.) In Equation (S.21), t_{-1}, t_{-2}, t_{-3}, etc. are all integration variables. The lowest-order term on the right-hand side features no integration. The next-lowest-order term on the right-hand side features a single integration over t_{-1}. The next-next-lowest-order term on the right-hand side features a double integration over t_{-2} and t_{-1}. This continues, so that higher-order terms on the right-hand side have higher-dimensional integrals that must be computed.

These integrals over $\beta(t)$ in Equation (S.21) can be done analytically, if possible, or can be performed using, e.g., Monte Carlo integration [7, 8]. Monte Carlo integration is a numerical technique in which random or pseudorandom numbers are used to approximately determine the value of a definite integral. To see how it works, consider $\beta(t)$ given by Equation (6) with $\beta_0 = 1$. For this choice of $\beta(t)$, if we want to numerically evaluate $\int_0^T dt_{-1}\, \beta(t_{-1})$, then we can pick a random number, u_{-1}, between 0 and T. If $\beta(u_{-1}) = 0$, then u_{-1} is recorded as "failure"; otherwise, u_{-1} is recorded as "success". This Bernoulli trial is repeated for many random values of u_{-1}. Our estimate for the value of the definite integral is then given simply by T multiplied by the fraction of trials that were successes. By increasing the number of such Bernoulli trials, we can achieve arbitrarily high precision in our numerical estimate of the definite integral. Also for this choice of $\beta(t)$, if we want to numerically evaluate $\int_0^T dt_{-1}\, \beta(t_{-1}) \int_0^{t_{-1}} dt_{-2}\, \beta(t_{-2})$, then we can pick two random numbers, u_{-1} and u_{-2}, each between 0 and T. If $u_{-2} > u_{-1}$, $\beta(u_{-1}) = 0$, or $\beta(u_{-2}) = 0$, then the trial pair of values u_{-1} and u_{-2} are recorded as "failure"; otherwise, the trial pair of values u_{-1} and u_{-2} are recorded as "success". This Bernoulli trial is repeated for many random pairs of values of u_{-1} and u_{-2}. Our estimate for the value of the definite integral is then given simply by T^2 multiplied by the fraction of trials that were successes. Monte Carlo integration is especially useful for evaluating high-dimensional integrals, which often do not feature nice analytical solutions; indeed, this approach is useful for numerically evaluating the high-dimensional integrals in the high-order terms resulting from the series expansion of Equation (S.21). (Although we assume the simple form of $\beta(t)$ given by Equation (6) for these examples of how to implement Monte Carlo integration, the analytical results in this section and the following two sections hold for arbitrary functional forms of $\beta(t)$, and for any case of $\beta(t)$, a variation of the Monte Carlo integration technique described above can be applied to evaluate the definite integrals that appear in our formulae.)

The series in Equation (S.21) converges to the quantity, M, that we are seeking: $y(L)$, when multiplied by M, yields $y(L + T)$. Thus, if $M > 1$, then, when sampled at times that are integer multiples of the drug-dosing period, the density of infected cells increases in time, and infection is established. Or, if $M < 1$, then, when sampled at times that are integer multiples of the drug-dosing period, the density of infected cells decreases in time, and infection is eliminated.

We can proceed further by substituting Equations (S.15) into Equation (S.20), which allows us to solve for the growth rate, $\tilde{r}_0$, of the viral strain when sampled at integer multiples of the drug-dosing period. Performing these substitutions, we obtain

$$\mathcal{M}y(L) = 0 \tag{S.22}$$

where $\mathcal{M}$ is given by

$$\mathcal{M} = -e^{\tilde{r}_0 T} + e^{-d_y T}\left[1 + \sum_{j=1}^{\infty}\left(\frac{\lambda}{d_x}\right)^j \int_0^T dt_{-1}\, \beta(t_{-1}) \prod_{k=2}^{j} \int_0^{t_{-k+1}} dt_{-k}\, \beta(t_{-k})\right] \tag{S.23}$$

For a nontrivial solution to Equation (S.22) (i.e., a solution for which $y(L)$ is nonzero), we can solve explicitly for $\tilde{r}_0$:

$$\tilde{r}_0 = -d_y + \frac{1}{T}\ln\left[1 + \sum_{j=1}^{\infty}\left(\frac{\lambda}{d_x}\right)^j \int_0^T dt_{-1}\, \beta(t_{-1}) \prod_{k=2}^{j} \int_0^{t_{-k+1}} dt_{-k}\, \beta(t_{-k})\right] \tag{S.24}$$

It is also helpful to define a quantity, $\tilde{R}_0$, which has the following properties:

- $\tilde{R}_0$ is nonnegative.
- $\tilde{R}_0 > 1$ if $\tilde{r}_0 > 0$, $\tilde{R}_0 < 1$ if $\tilde{r}_0 < 0$, and $\tilde{R}_0 = 1$ if $\tilde{r}_0 = 0$. Thus, the condition $\tilde{R}_0 > 1$ indicates the establishment of infection, while the condition $\tilde{R}_0 < 1$ indicates the elimination of infection.
- If $\beta(t) = \beta$ is constant, then $\tilde{R}_0$ simply reduces to the basic reproductive ratio, denoted here as R_0.

Thus, the quantity $\tilde{R}_0$ for the case of a time-varying drug effectiveness, $\beta(t)$, is analogous to the basic reproductive ratio that is widely used for analyzing the more-familiar case of a constant value of β [9]. We therefore define the parameter $\tilde{R}_0$ as

$$\tilde{R}_0 \equiv 1 + \frac{\tilde{r}_0}{d_y} \qquad \text{for instantaneous maturation} \tag{S.25}$$

The exercise above aids in understanding the derivations that follow for more complicated cases, but as a consistency check, $\tilde{r}_0$ in Equation (S.24) and $\tilde{R}_0$ in Equation (S.25) must also be given much more simply by r_0 and R_0 for the case of constant drug levels if we replace β in those simple formulae with the time-average of $\beta(t)$ over a single drug-dosing period, denoted by $\langle\beta(t)\rangle$.

This simplification is quite intuitive. In Equation (S.24), notice that

$$\int_0^T dt_{-1}\, \beta(t_{-1}) \prod_{k=2}^{j} \int_0^{t_{-k+1}} dt_{-k}\, \beta(t_{-k}) = \frac{\langle\beta(t)\rangle^j T^j}{j!} \quad \forall\, j \geq 1$$

Thus, Equation (S.24) becomes

$$\tilde{r}_0 = -d_y + \frac{1}{T}\ln\left[1 + \frac{\lambda\langle\beta(t)\rangle T}{d_x} + \frac{1}{2}\left(\frac{\lambda\langle\beta(t)\rangle T}{d_x}\right)^2 + \frac{1}{3!}\left(\frac{\lambda\langle\beta(t)\rangle T}{d_x}\right)^3 + \cdots\right]$$

We simply have

$$\tilde{r}_0 = -d_y + \frac{\lambda\langle\beta(t)\rangle}{d_x} \tag{S.26}$$

Substituting Equation (S.26) into Equation (S.25), we obtain

$$\tilde{R}_0 = \frac{\lambda\langle\beta(t)\rangle}{d_x d_y} \tag{S.27}$$

Equations (S.26) and (S.27) are exactly as expected if there are no intermediate maturation steps and no maturation delay [5].

6.2 The maturation process happens in n intermediate maturation steps

We now apply a variation of the procedure used for the case of no intermediate maturation steps to the case of a finite number, n, of intermediate maturation steps. Again, we have a time-dependent and periodic $\beta(t) = \beta(t+T)$. The viral dynamics are given by

$$\begin{aligned}
\dot{x}(t) &= \lambda - \beta(t)x(t)y(t) - d_x x(t) \\
\dot{w}_1(t) &= \beta(t)x(t)y(t) - (m_1 + d_1)w_1(t) \\
\dot{w}_i(t) &= m_{i-1}w_{i-1}(t) - (m_i + d_i)w_i(t) \quad \forall\, 2 \leq i \leq n \\
\dot{y}(t) &= m_n w_n(t) - d_y y(t).
\end{aligned}$$

We assume that the system starts from the uninfected equilibrium, and a small amount of the virus is introduced. If we focus on the early-time dynamics, when the density of infected cells is low, then we have

$$\begin{aligned}
\dot{y}(t) &= m_n w_n(t) - d_y y(t) \\
\dot{w}_1(t) &= \frac{\lambda}{d_x}\beta(t)y(t) - (m_1 + d_1)w_1(t) \\
\dot{w}_i(t) &= m_{i-1}w_{i-1}(t) - (m_i + d_i)w_i(t) \quad \forall\, 2 \leq i \leq n
\end{aligned} \tag{S.28}$$

(Here, we choose to write the equation for $\dot{y}(t)$ first simply for notational convenience, as will become apparent.) Similarly to the analysis in the previous section, we can use an integrating factor on each equation and then integrate each resulting equation between time L and time $L + t_k$ to obtain

$$\begin{aligned}
e^{d_y t_k} y(L + t_k) &= y(L) \\
&\quad + m_n \int_0^{t_k} dt_{k-1}\; e^{(d_y - m_n - d_n)t_{k-1}} e^{(m_n + d_n)t_{k-1}} w_n(L + t_{k-1}) \\
e^{(m_1 + d_1)t_k} w_1(L + t_k) &= w_1(L) \\
&\quad + \frac{\lambda}{d_x} \int_0^{t_k} dt_{k-1}\; \beta(t_{k-1}) e^{(m_1 + d_1 - d_y)t_{k-1}} e^{d_y t_{k-1}} y(L + t_{k-1}) \\
e^{(m_i + d_i)t_k} w_i(L + t_k) &= w_i(L) \\
&\quad + m_{i-1} \int_0^{t_k} dt_{k-1}\; e^{(m_i + d_i - m_{i-1} - d_{i-1})t_{k-1}} \\
&\quad \times e^{(m_{i-1} + d_{i-1})t_{k-1}} w_{i-1}(L + t_{k-1}) \quad \forall\, 2 \leq i \leq n
\end{aligned} \tag{S.29}$$

(Here, just as in the previous section, L and t_k are any early times in the dynamics such that the density of infected cells is low, so that Equations (S.28) hold. The notation t_{k-1} for the integration variable is convenient, as it implies the manipulations that are to follow. Also, for simplicity of notation, we assume that L is equal to an integer multiple of T, so that $\beta(L+t_{k-1}) = \beta(t_{k-1})$.)

Our approach is similar to that of the previous section: We would like to find a linear operator that tells us how $y(L)$ and $w_i(L)$ for all $1 \le i \le n$ are related to $y(L+T)$ and $w_i(L+T)$ for all $1 \le i \le n$. It is helpful to start by exploring Equations (S.29) for some small values of n.

Particular case: $n = 1$

For the case $n = 1$, there is a single intermediate maturation step. In this case, Equations (S.29) reduce to the following two equations:

$$
\begin{aligned}
e^{d_y t_k} y(L+t_k) &= y(L) \\
&\quad + m_1 \int_0^{t_k} dt_{k-1}\; e^{(d_y - m_1 - d_1)t_{k-1}} e^{(m_1+d_1)t_{k-1}} w_1(L+t_{k-1}) \\
e^{(m_1+d_1)t_k} w_1(L+t_k) &= w_1(L) \\
&\quad + \frac{\lambda}{d_x} \int_0^{t_k} dt_{k-1}\; \beta(t_{k-1}) e^{(m_1+d_1-d_y)t_{k-1}} e^{d_y t_{k-1}} y(L+t_{k-1})
\end{aligned}
\tag{S.30}
$$

If we set $k = 0$ and $t_0 = T$ in Equations (S.30), then we obtain

$$
\begin{aligned}
e^{d_y T} y(L+T) &= y(L) \\
&\quad + m_1 \int_0^{T} dt_{-1}\; e^{(d_y - m_1 - d_1)t_{-1}} e^{(m_1+d_1)t_{-1}} w_1(L+t_{-1}) \\
e^{(m_1+d_1)T} w_1(L+T) &= w_1(L) \\
&\quad + \frac{\lambda}{d_x} \int_0^{T} dt_{-1}\; \beta(t_{-1}) e^{(m_1+d_1-d_y)t_{-1}} e^{d_y t_{-1}} y(L+t_{-1})
\end{aligned}
\tag{S.31}
$$

Next, we substitute the first of Equations (S.30) into the second of Equations (S.31), and we

substitute the second of Equations (S.30) into the first of Equations (S.31); we obtain

$$
\begin{aligned}
e^{d_y T} y(L+T) &= y(L) \\
&+ w_1(L) m_1 \int_0^T dt_{-1}\, e^{(d_y - m_1 - d_1)t_{-1}} \\
&+ m_1 \frac{\lambda}{d_x} \int_0^T dt_{-1}\, e^{(d_y - m_1 - d_1)t_{-1}} \\
&\times \int_0^{t_{-1}} dt_{-2}\, \beta(t_{-2}) e^{(m_1 + d_1 - d_y)t_{-2}} e^{d_y t_{-2}} y(L + t_{-2}) \\
e^{(m_1+d_1)T} w_1(L+T) &= w_1(L) \\
&+ y(L) \frac{\lambda}{d_x} \int_0^T dt_{-1}\, \beta(t_{-1}) e^{(m_1 + d_1 - d_y)t_{-1}} \\
&+ \frac{\lambda}{d_x} m_1 \int_0^T dt_{-1}\, \beta(t_{-1}) e^{(m_1 + d_1 - d_y)t_{-1}} \\
&\times \int_0^{t_{-1}} dt_{-2}\, e^{(d_y - m_1 - d_1)t_{-2}} e^{(m_1 + d_1)t_{-2}} w_1(L + t_{-2})
\end{aligned}
\tag{S.32}
$$

Next, we substitute the first of Equations (S.30) into the first of Equations (S.32), and we substitute the second of Equations (S.30) into the second of Equations (S.32); we obtain

$$
\begin{aligned}
e^{d_y T} y(L+T) &= y(L) \\
&+ w_1(L) m_1 \int_0^T dt_{-1}\, e^{(d_y - m_1 - d_1)t_{-1}} \\
&+ y(L) m_1 \frac{\lambda}{d_x} \int_0^T dt_{-1}\, e^{(d_y - m_1 - d_1)t_{-1}} \\
&\times \int_0^{t_{-1}} dt_{-2}\, \beta(t_{-2}) e^{(m_1 + d_1 - d_y)t_{-2}} \\
&+ m_1^2 \frac{\lambda}{d_x} \int_0^T dt_{-1}\, e^{(d_y - m_1 - d_1)t_{-1}} \\
&\times \int_0^{t_{-1}} dt_{-2}\, \beta(t_{-2}) e^{(m_1 + d_1 - d_y)t_{-2}} \\
&\times \int_0^{t_{-2}} dt_{-3}\, e^{(d_y - m_1 - d_1)t_{-3}} e^{(m_1 + d_1)t_{-3}} w_1(L + t_{-3}) \\
e^{(m_1 + d_1)T} w_1(L+T) &= w_1(L) \\
&+ y(L) \frac{\lambda}{d_x} \int_0^T dt_{-1}\, \beta(t_{-1}) e^{(m_1 + d_1 - d_y)t_{-1}} \\
&+ w_1(L) \frac{\lambda}{d_x} m_1 \int_0^T dt_{-1}\, \beta(t_{-1}) e^{(m_1 + d_1 - d_y)t_{-1}} \\
&\times \int_0^{t_{-1}} dt_{-2}\, e^{(d_y - m_1 - d_1)t_{-2}} \\
&+ \left(\frac{\lambda}{d_x}\right)^2 m_1 \int_0^T dt_{-1}\, \beta(t_{-1}) e^{(m_1 + d_1 - d_y)t_{-1}} \\
&\times \int_0^{t_{-1}} dt_{-2}\, e^{(d_y - m_1 - d_1)t_{-2}} \\
&\times \int_0^{t_{-2}} dt_{-3}\, \beta(t_{-3}) e^{(m_1 + d_1 - d_y)t_{-3}} e^{d_y t_{-3}} y(L + t_{-3})
\end{aligned}
\tag{S.33}
$$

Next, we substitute the first of Equations (S.30) into the second of Equations (S.33), and we substitute the second of Equations (S.30) into the first of Equations (S.33). Notice that if we continue this procedure *ad infinitum*, then $y(L+T)$ and $w_1(L+T)$ can be expressed as

$$
\begin{pmatrix} y(L+T) \\ w_1(L+T) \end{pmatrix} = \begin{pmatrix} M_{00} & M_{01} \\ M_{10} & M_{11} \end{pmatrix} \begin{pmatrix} y(L) \\ w_1(L) \end{pmatrix}
\tag{S.34}
$$

where the M_{ij} are infinite series that are functions of the model parameters and functionals of the infectivity, $\beta(t)$.

Particular case: $n = 2$

For the case $n = 2$, there are two intermediate maturation steps. In this case, Equations (S.29)

reduce to the following three equations:

$$
\begin{aligned}
e^{d_y t_k} y(L+t_k) &= y(L) \\
&\quad + m_2 \int_0^{t_k} dt_{k-1}\, e^{(d_y-m_2-d_2)t_{k-1}} e^{(m_2+d_2)t_{k-1}} w_2(L+t_{k-1}) \\
e^{(m_1+d_1)t_k} w_1(L+t_k) &= w_1(L) \\
&\quad + \frac{\lambda}{d_x} \int_0^{t_k} dt_{k-1}\, \beta(t_{k-1}) e^{(m_1+d_1-d_y)t_{k-1}} e^{d_y t_{k-1}} y(L+t_{k-1}) \\
e^{(m_2+d_2)t_k} w_2(L+t_k) &= w_2(L) \\
&\quad + m_1 \int_0^{t_k} dt_{k-1}\, e^{(m_2+d_2-m_1-d_1)t_{k-1}} e^{(m_1+d_1)t_{k-1}} w_1(L+t_{k-1})
\end{aligned}
\tag{S.35}
$$

If we set $k=0$ and $t_0=T$ in Equations (S.35), then we obtain

$$
\begin{aligned}
e^{d_y T} y(L+T) &= y(L) \\
&\quad + m_2 \int_0^{T} dt_{-1}\, e^{(d_y-m_2-d_2)t_{-1}} e^{(m_2+d_2)t_{-1}} w_2(L+t_{-1}) \\
e^{(m_1+d_1)T} w_1(L+T) &= w_1(L) \\
&\quad + \frac{\lambda}{d_x} \int_0^{T} dt_{-1}\, \beta(t_{-1}) e^{(m_1+d_1-d_y)t_{-1}} e^{d_y t_{-1}} y(L+t_{-1}) \\
e^{(m_2+d_2)T} w_2(L+T) &= w_2(L) \\
&\quad + m_1 \int_0^{T} dt_{-1}\, e^{(m_2+d_2-m_1-d_1)t_{-1}} e^{(m_1+d_1)t_{-1}} w_1(L+t_{-1})
\end{aligned}
\tag{S.36}
$$

Next, we substitute the first of Equations (S.35) into the second of Equations (S.36), we substitute the second of Equations (S.35) into the third of Equations (S.36), and we substitute the third of

Equations (S.35) into the first of Equations (S.36); we obtain

$$
\begin{aligned}
e^{d_y T} y(L+T) &= y(L) \\
&+ w_2(L) m_2 \int_0^T dt_{-1}\, e^{(d_y - m_2 - d_2)t_{-1}} \\
&+ m_2 m_1 \int_0^T dt_{-1}\, e^{(d_y - m_2 - d_2)t_{-1}} \\
&\times \int_0^{t_{-1}} dt_{-2}\, e^{(m_2 + d_2 - m_1 - d_1)t_{-2}} e^{(m_1 + d_1)t_{-2}} w_1(L + t_{-2}) \\
e^{(m_1+d_1)T} w_1(L+T) &= w_1(L) \\
&+ y(L) \frac{\lambda}{d_x} \int_0^T dt_{-1}\, \beta(t_{-1}) e^{(m_1 + d_1 - d_y)t_{-1}} \\
&+ \frac{\lambda}{d_x} m_2 \int_0^T dt_{-1}\, \beta(t_{-1}) e^{(m_1 + d_1 - d_y)t_{-1}} \\
&\times \int_0^{t_{-1}} dt_{-2}\, e^{(d_y - m_2 - d_2)t_{-2}} e^{(m_2 + d_2)t_{-2}} w_2(L + t_{-2}) \\
e^{(m_2+d_2)T} w_2(L+T) &= w_2(L) \\
&+ w_1(L) m_1 \int_0^T dt_{-1}\, e^{(m_2 + d_2 - m_1 - d_1)t_{-1}} \\
&+ m_1 \frac{\lambda}{d_x} \int_0^T dt_{-1}\, e^{(m_2 + d_2 - m_1 - d_1)t_{-1}} \\
&\times \int_0^{t_{-1}} dt_{-2}\, \beta(t_{-2}) e^{(m_1 + d_1 - d_y)t_{-2}} e^{d_y t_{-2}} y(L + t_{-2})
\end{aligned}
\tag{S.37}
$$

Next, we substitute the first of Equations (S.35) into the third of Equations (S.37), we substitute the second of Equations (S.35) into the first of Equations (S.37), and we substitute the third of

Equations (S.35) into the second of Equations (S.37); we obtain

$$
\begin{aligned}
e^{d_y T} y(L+T) &= y(L) \\
&+ w_2(L) m_2 \int_0^T dt_{-1}\; e^{(d_y - m_2 - d_2)t_{-1}} \\
&+ w_1(L) m_2 m_1 \int_0^T dt_{-1}\; e^{(d_y - m_2 - d_2)t_{-1}} \\
&\times \int_0^{t_{-1}} dt_{-2}\; e^{(m_2 + d_2 - m_1 - d_1)t_{-2}} \\
&+ m_2 m_1 \frac{\lambda}{d_x} \int_0^T dt_{-1}\; e^{(d_y - m_2 - d_2)t_{-1}} \\
&\times \int_0^{t_{-1}} dt_{-2}\; e^{(m_2 + d_2 - m_1 - d_1)t_{-2}} \\
&\times \int_0^{t_{-2}} dt_{-3}\; \beta(t_{-3}) e^{(m_1 + d_1 - d_y)t_{-3}} e^{d_y t_{-3}} y(L + t_{-3}) \\
e^{(m_1+d_1)T} w_1(L+T) &= w_1(L) \\
&+ y(L) \frac{\lambda}{d_x} \int_0^T dt_{-1}\; \beta(t_{-1}) e^{(m_1 + d_1 - d_y)t_{-1}} \\
&+ w_2(L) \frac{\lambda}{d_x} m_2 \int_0^T dt_{-1}\; \beta(t_{-1}) e^{(m_1 + d_1 - d_y)t_{-1}} \\
&\times \int_0^{t_{-1}} dt_{-2}\; e^{(d_y - m_2 - d_2)t_{-2}} \\
&+ \frac{\lambda}{d_x} m_2 m_1 \int_0^T dt_{-1}\; \beta(t_{-1}) e^{(m_1 + d_1 - d_y)t_{-1}} \\
&\times \int_0^{t_{-1}} dt_{-2}\; e^{(d_y - m_2 - d_2)t_{-2}} \\
&\times \int_0^{t_{-2}} dt_{-3}\; e^{(m_2 + d_2 - m_1 - d_1)t_{-3}} e^{(m_1 + d_1)t_{-3}} w_1(L + t_{-3}) \\
e^{(m_2+d_2)T} w_2(L+T) &= w_2(L) \\
&+ w_1(L) m_1 \int_0^T dt_{-1}\; e^{(m_2 + d_2 - m_1 - d_1)t_{-1}} \\
&+ y(L) m_1 \frac{\lambda}{d_x} \int_0^T dt_{-1}\; e^{(m_2 + d_2 - m_1 - d_1)t_{-1}} \\
&\times \int_0^{t_{-1}} dt_{-2}\; \beta(t_{-2}) e^{(m_1 + d_1 - d_y)t_{-2}} \\
&+ m_1 \frac{\lambda}{d_x} m_2 \int_0^T dt_{-1}\; e^{(m_2 + d_2 - m_1 - d_1)t_{-1}} \\
&\times \int_0^{t_{-1}} dt_{-2}\; \beta(t_{-2}) e^{(m_1 + d_1 - d_y)t_{-2}} \\
&\times \int_0^{t_{-2}} dt_{-3}\; e^{(d_y - m_2 - d_2)t_{-3}} e^{(m_2 + d_2)t_{-3}} w_2(L + t_{-3})
\end{aligned}
\tag{S.38}
$$

Next, we substitute the first of Equations (S.35) into the first of Equations (S.38), we substitute the second of Equations (S.35) into the second of Equations (S.38), and we substitute the third of Equations (S.35) into the third of Equations (S.38). Notice that if we continue this procedure *ad infinitum*, then $y(L+T)$, $w_1(L+T)$, and $w_2(L+T)$ can be expressed as

$$\begin{pmatrix} y(L+T) \\ w_1(L+T) \\ w_2(L+T) \end{pmatrix} = \begin{pmatrix} M_{00} & M_{01} & M_{02} \\ M_{10} & M_{11} & M_{12} \\ M_{20} & M_{21} & M_{22} \end{pmatrix} \begin{pmatrix} y(L) \\ w_1(L) \\ w_2(L) \end{pmatrix} \tag{S.39}$$

where the M_{ij} are infinite series that are functions of the model parameters and functionals of the infectivity, $\beta(t)$.

Case of any $n \geq 1$

Following the procedure described above, for any $n \geq 1$, we obtain

$$\begin{pmatrix} y(L+T) \\ w_1(L+T) \\ \vdots \\ w_n(L+T) \end{pmatrix} = \begin{pmatrix} M_{00} & M_{01} & \dots & M_{0n} \\ M_{10} & M_{11} & & \vdots \\ \vdots & & \ddots & \vdots \\ M_{n0} & \dots & \dots & M_{nn} \end{pmatrix} \begin{pmatrix} y(L) \\ w_1(L) \\ \vdots \\ w_n(L) \end{pmatrix} \tag{S.40}$$

Recall that our goal is to solve for the linear operator, M_{ij}. Although the procedure outlined above shows us how to do this, it is evident that the mathematical expressions are symbolically quite cumbersome. Therefore, we simplify notation substantially by making several definitions.

First, the notation in Equations (S.40) is more natural if we define

$$w_0(t) \equiv y(t)$$

Then, Equations (S.40) simply feature $w_i(L)$ and $w_i(L+T)$ for $0 \leq i \leq n$, which is notationally much more convenient.

Next, since the dynamical quantities, $w_i(t)$, are indexed by $0 \leq i \leq n$, and since each M_{ij} is an infinite series in which factors of $w_i(L)$ appear in terms in cyclical fashion, it is natural to define

$$\Lambda(i,n) \equiv i \bmod (n+1)$$

Then, for any integer value of i, the function $\Lambda(i,n)$ simply outputs an integer between 0 inclusive and n inclusive.

Again, noticing that each M_{ij} is an infinite series in which factors appear in terms in cyclical fashion, we strive to simplify notation in the series expansion of each M_{ij} to the maximum extent possible. We therefore make the following definitions, whose notational utility will become apparent:

$$\Gamma_i \equiv \begin{cases} \frac{\lambda}{d_x}, & \text{if } \Lambda(i,n) = 0 \\ m_{\Lambda(i,n)}, & \text{if } 1 \leq \Lambda(i,n) \leq n \end{cases}$$

$$\gamma_i \equiv \begin{cases} d_y, & \text{if } \Lambda(i,n) = 0 \\ m_{\Lambda(i,n)} + d_{\Lambda(i,n)}, & \text{if } 1 \leq \Lambda(i,n) \leq n \end{cases}$$

$$K_i(t_k) \equiv \begin{cases} \beta(t_k) e^{(\gamma_1 - \gamma_0) t_k}, & \text{if } \Lambda(i,n) = 0 \\ e^{(\gamma_{i+1} - \gamma_i) t_k}, & \text{if } 1 \leq \Lambda(i,n) \leq n \end{cases}$$

$$\xi_i(q,t_0) \equiv \Gamma_q \times \begin{cases} \int_0^{t_0} dt_{-1}\ K_q(t_{-1}), & \text{if } i=-1 \\ \int_0^{t_{i+1}} dt_i\ K_q(t_i), & \text{if } i<-1 \end{cases}$$

$$F_q(t_k) \equiv e^{\gamma_q t_k} w_q(L+t_k)$$

Now, with the preceding definitions, notice that Equations (S.29) can be written very compactly:

$$F_{\Lambda(q,n)}(t_k) = F_{\Lambda(q,n)}(0) + \xi_{k-1}(q-1,t_k) F_{\Lambda(q-1,n)}(t_{k-1}) \tag{S.41}$$

In Equations (S.41), $k \le 0$, and q can be any integer.

Finally, notice that in Equations (S.33) for $n=1$ and in Equations (S.38) for $n=2$, each integral over t_{-1} has an upper limit of $t_{-1}=T$. Therefore, it simplifies notation further if we define

$$\xi_i(q) \equiv \xi_i(q,T)$$

Let us now revisit our calculations for the simple cases of $n=1$ and $n=2$ to see how the notation with the above substitutions is dramatically simplified.

Particular case: $n=1$

For the case $n=1$, recall that, depending on the value of i, the function $\Lambda(i,n)$ reduces to 0 or 1. Therefore, with the definitions above, Equations (S.41) reduce to the following two equations:

$$\begin{aligned} F_{\Lambda(0,1)}(t_k) &= F_{\Lambda(0,1)}(0) \\ &\quad + \xi_{k-1}(-1) F_{\Lambda(-1,1)}(t_{k-1}) \\ F_{\Lambda(1,1)}(t_k) &= F_{\Lambda(1,1)}(0) \\ &\quad + \xi_{k-1}(0) F_{\Lambda(0,1)}(t_{k-1}) \end{aligned} \tag{S.42}$$

If we set $k=0$ and $t_0=T$ in Equations (S.42), then we obtain

$$\begin{aligned} F_{\Lambda(0,1)}(T) &= F_{\Lambda(0,1)}(0) \\ &\quad + \xi_{-1}(-1) F_{\Lambda(-1,1)}(t_{-1}) \\ F_{\Lambda(1,1)}(T) &= F_{\Lambda(1,1)}(0) \\ &\quad + \xi_{-1}(0) F_{\Lambda(0,1)}(t_{-1}) \end{aligned} \tag{S.43}$$

Next, we substitute the first of Equations (S.42) into the second of Equations (S.43), and we substitute the second of Equations (S.42) into the first of Equations (S.43); we obtain

$$\begin{aligned} F_{\Lambda(0,1)}(T) &= F_{\Lambda(0,1)}(0) \\ &\quad + \xi_{-1}(-1) F_{\Lambda(-1,1)}(0) \\ &\quad + \xi_{-1}(-1)\xi_{-2}(-2) F_{\Lambda(-2,1)}(t_{-2}) \\ F_{\Lambda(1,1)}(T) &= F_{\Lambda(1,1)}(0) \\ &\quad + \xi_{-1}(0) F_{\Lambda(0,1)}(0) \\ &\quad + \xi_{-1}(0)\xi_{-2}(-1) F_{\Lambda(-1,1)}(t_{-2}) \end{aligned} \tag{S.44}$$

Next, we substitute the first of Equations (S.42) into the first of Equations (S.44), and we substitute the second of Equations (S.42) into the second of Equations (S.44); we obtain

$$\begin{aligned}
F_{\Lambda(0,1)}(T) &= F_{\Lambda(0,1)}(0) \\
&\quad + \xi_{-1}(-1)F_{\Lambda(-1,1)}(0) \\
&\quad + \xi_{-1}(-1)\xi_{-2}(-2)F_{\Lambda(-2,1)}(0) \\
&\quad + \xi_{-1}(-1)\xi_{-2}(-2)\xi_{-3}(-3)F_{\Lambda(-3,1)}(t_{-3}) \\
F_{\Lambda(1,1)}(T) &= F_{\Lambda(1,1)}(0) \\
&\quad + \xi_{-1}(0)F_{\Lambda(0,1)}(0) \\
&\quad + \xi_{-1}(0)\xi_{-2}(-1)F_{\Lambda(-1,1)}(0) \\
&\quad + \xi_{-1}(0)\xi_{-2}(-1)\xi_{-3}(-2)F_{\Lambda(-2,1)}(t_{-3})
\end{aligned} \tag{S.45}$$

Next, we substitute the first of Equations (S.42) into the second of Equations (S.45), and we substitute the second of Equations (S.42) into the first of Equations (S.45). We continue this procedure *ad infinitum*. Using the definitions above, it can be verified by direct substitution that Equations (S.42), (S.43), (S.44), and (S.45) reduce to Equations (S.30), (S.31), (S.32), and (S.33), respectively. However, the notational and conceptual utility of the substitutions described above for simplifying the writing is evident.

We see that the M_{ij} in Equations (S.34) are given by

$$\begin{aligned}
M_{00} &= e^{-\gamma_0 T}\left[1 + \xi_{-1}(-1)\xi_{-2}(-2) + \cdots\right] \\
M_{01} &= e^{-\gamma_0 T}\left[\xi_{-1}(-1) + \xi_{-1}(-1)\xi_{-2}(-2)\xi_{-3}(-3) + \cdots\right] \\
M_{10} &= e^{-\gamma_1 T}\left[\xi_{-1}(0) + \xi_{-1}(0)\xi_{-2}(-1)\xi_{-3}(-2) + \cdots\right] \\
M_{11} &= e^{-\gamma_1 T}\left[1 + \xi_{-1}(0)\xi_{-2}(-1) + \cdots\right]
\end{aligned}$$

Particular case: $n = 2$

For the case $n = 2$, recall that, depending on the value of i, the function $\Lambda(i, n)$ reduces to 0, 1, or 2. Therefore, with the definitions above, Equations (S.41) reduce to the following three equations:

$$\begin{aligned}
F_{\Lambda(0,2)}(t_k) &= F_{\Lambda(0,2)}(0) \\
&\quad + \xi_{k-1}(-1)F_{\Lambda(-1,2)}(t_{k-1}) \\
F_{\Lambda(1,2)}(t_k) &= F_{\Lambda(1,2)}(0) \\
&\quad + \xi_{k-1}(0)F_{\Lambda(0,2)}(t_{k-1}) \\
F_{\Lambda(2,2)}(t_k) &= F_{\Lambda(2,2)}(0) \\
&\quad + \xi_{k-1}(1)F_{\Lambda(1,2)}(t_{k-1})
\end{aligned} \tag{S.46}$$

If we set $k = 0$ and $t_0 = T$ in Equations (S.46), then we obtain

$$\begin{aligned} F_{\Lambda(0,2)}(T) &= F_{\Lambda(0,2)}(0) \\ &\quad + \xi_{-1}(-1)F_{\Lambda(-1,2)}(t_{-1}) \\ F_{\Lambda(1,2)}(T) &= F_{\Lambda(1,2)}(0) \\ &\quad + \xi_{-1}(0)F_{\Lambda(0,2)}(t_{-1}) \\ F_{\Lambda(2,2)}(T) &= F_{\Lambda(2,2)}(0) \\ &\quad + \xi_{-1}(1)F_{\Lambda(1,2)}(t_{-1}) \end{aligned} \tag{S.47}$$

Next, we substitute the first of Equations (S.46) into the second of Equations (S.47), we substitute the second of Equations (S.46) into the third of Equations (S.47), and we substitute the third of Equations (S.46) into the first of Equations (S.47); we obtain

$$\begin{aligned} F_{\Lambda(0,2)}(T) &= F_{\Lambda(0,2)}(0) \\ &\quad + \xi_{-1}(-1)F_{\Lambda(-1,2)}(0) \\ &\quad + \xi_{-1}(-1)\xi_{-2}(-2)F_{\Lambda(-2,2)}(t_{-2}) \\ F_{\Lambda(1,2)}(T) &= F_{\Lambda(1,2)}(0) \\ &\quad + \xi_{-1}(0)F_{\Lambda(0,2)}(0) \\ &\quad + \xi_{-1}(0)\xi_{-2}(-1)F_{\Lambda(-1,2)}(t_{-2}) \\ F_{\Lambda(2,2)}(T) &= F_{\Lambda(2,2)}(0) \\ &\quad + \xi_{-1}(1)F_{\Lambda(1,2)}(0) \\ &\quad + \xi_{-1}(1)\xi_{-2}(0)F_{\Lambda(0,2)}(t_{-2}) \end{aligned} \tag{S.48}$$

Next, we substitute the first of Equations (S.46) into the third of Equations (S.48), we substitute the second of Equations (S.46) into the first of Equations (S.48), and we substitute the third of Equations (S.46) into the second of Equations (S.48); we obtain

$$\begin{aligned} F_{\Lambda(0,2)}(T) &= F_{\Lambda(0,2)}(0) \\ &\quad + \xi_{-1}(-1)F_{\Lambda(-1,2)}(0) \\ &\quad + \xi_{-1}(-1)\xi_{-2}(-2)F_{\Lambda(-2,2)}(0) \\ &\quad + \xi_{-1}(-1)\xi_{-2}(-2)\xi_{-3}(-3)F_{\Lambda(-3,2)}(t_{-3}) \\ F_{\Lambda(1,2)}(T) &= F_{\Lambda(1,2)}(0) \\ &\quad + \xi_{-1}(0)F_{\Lambda(0,2)}(0) \\ &\quad + \xi_{-1}(0)\xi_{-2}(-1)F_{\Lambda(-1,2)}(0) \\ &\quad + \xi_{-1}(0)\xi_{-2}(-1)\xi_{-3}(-2)F_{\Lambda(-2,2)}(t_{-3}) \\ F_{\Lambda(2,2)}(T) &= F_{\Lambda(2,2)}(0) \\ &\quad + \xi_{-1}(1)F_{\Lambda(1,2)}(0) \\ &\quad + \xi_{-1}(1)\xi_{-2}(0)F_{\Lambda(0,2)}(0) \\ &\quad + \xi_{-1}(1)\xi_{-2}(0)\xi_{-3}(-1)F_{\Lambda(-1,2)}(t_{-3}) \end{aligned} \tag{S.49}$$

Next, we substitute the first of Equations (S.46) into the first of Equations (S.49), we substitute the second of Equations (S.46) into the second of Equations (S.49), and we substitute the third of Equations (S.46) into the third of Equations (S.49). We continue this procedure *ad infinitum.* Using the definitions above, it can be verified by direct substitution that Equations (S.46), (S.47), (S.48), and (S.49) reduce to Equations (S.35), (S.36), (S.37), and (S.38), respectively. However, the notational and conceptual utility of the substitutions described above for simplifying the writing is again evident.

We see that the M_{ij} in Equations (S.39) are given by

$$
\begin{aligned}
M_{00} &= e^{-\gamma_0 T}\left[1 + \xi_{-1}(-1)\xi_{-2}(-2)\xi_{-3}(-3) + \cdots\right] \\
M_{01} &= e^{-\gamma_0 T}\left[\xi_{-1}(-1)\xi_{-2}(-2) + \xi_{-1}(-1)\xi_{-2}(-2)\xi_{-3}(-3)\xi_{-4}(-4)\xi_{-5}(-5) + \cdots\right] \\
M_{02} &= e^{-\gamma_0 T}\left[\xi_{-1}(-1) + \xi_{-1}(-1)\xi_{-2}(-2)\xi_{-3}(-3)\xi_{-4}(-4) + \cdots\right] \\
M_{10} &= e^{-\gamma_1 T}\left[\xi_{-1}(0) + \xi_{-1}(0)\xi_{-2}(-1)\xi_{-3}(-2)\xi_{-4}(-3) + \cdots\right] \\
M_{11} &= e^{-\gamma_1 T}\left[1 + \xi_{-1}(0)\xi_{-2}(-1)\xi_{-3}(-2) + \cdots\right] \\
M_{12} &= e^{-\gamma_1 T}\left[\xi_{-1}(0)\xi_{-2}(-1) + \xi_{-1}(0)\xi_{-2}(-1)\xi_{-3}(-2)\xi_{-4}(-3)\xi_{-5}(-4) + \cdots\right] \\
M_{20} &= e^{-\gamma_2 T}\left[\xi_{-1}(1)\xi_{-2}(0) + \xi_{-1}(1)\xi_{-2}(0)\xi_{-3}(-1)\xi_{-4}(-2)\xi_{-5}(-3) + \cdots\right] \\
M_{21} &= e^{-\gamma_2 T}\left[\xi_{-1}(1) + \xi_{-1}(1)\xi_{-2}(0)\xi_{-3}(-1)\xi_{-4}(-2) + \cdots\right] \\
M_{22} &= e^{-\gamma_2 T}\left[1 + \xi_{-1}(1)\xi_{-2}(0)\xi_{-3}(-1) + \cdots\right]
\end{aligned}
$$

Case of any $n \geq 1$

If we set $k = 0$ and $t_0 = T$ in Equations (S.41), then we obtain $n + 1$ equations:

$$F_{\Lambda(q,n)}(T) = F_{\Lambda(q,n)}(0) + \xi_{-1}(q-1)F_{\Lambda(q-1,n)}(t_{-1}) \tag{S.50}$$

By repeatedly substituting for $F_{\Lambda(q-1,n)}(t_{-1})$, $F_{\Lambda(q-2,n)}(t_{-2})$, etc. in Equations (S.50), the $n + 1$ equations become

$$F_{\Lambda(q,n)}(T) = F_{\Lambda(q,n)}(0) + \sum_{p=1}^{\infty} F_{\Lambda(q-p,n)}(0) \prod_{k=1}^{p} \xi_{-k}(q-k)$$

Equivalently,

$$w_{\Lambda(q,n)}(L+T) = e^{-\gamma_q T}\left[w_{\Lambda(q,n)}(L) + \sum_{p=1}^{\infty} w_{\Lambda(q-p,n)}(L) \prod_{k=1}^{p} \xi_{-k}(q-k)\right] \tag{S.51}$$

From Equations (S.51), we see that the M_{ij} in Equations (S.40) are given by

$$
\begin{aligned}
M_{ij} = e^{-\gamma_i T} &\left(\prod_{\ell=1}^{\Lambda(i-j,n)} \xi_{-\ell}(i-\ell)\right) \\
&\times \sum_{p=0}^{\infty} \prod_{q=0}^{p-1} \prod_{k=1}^{n+1} \xi_{-\Lambda(i-j,n)-(n+1)q-k}(i - \Lambda(i-j,n) - (n+1)q - k)
\end{aligned}
\tag{S.52}
$$

The integrals over $\beta(t)$ in Equation (S.52) can be done analytically, if possible, or can be performed using, e.g., Monte Carlo integration. (Please see the section on the derivation of $\tilde{r}_0$ for the case of no intermediate maturation steps for a description of how Monte Carlo integration works.)

The series in Equation (S.52) converges to the linear operator, M_{ij}, that we are seeking. (In the language of Floquet theory, M_{ij} is the monodromy matrix.) Thus, if the largest eigenvalue of M_{ij} is greater than 1, then, when sampled at times that are integer multiples of the drug-dosing period, the density of infected cells increases in time, and infection is established. Or, if the largest eigenvalue of M_{ij} is less than 1, then, when sampled at times that are integer multiples of the drug-dosing period, the density of infected cells decreases in time, and infection is eliminated.

Similarly to the case of no intermediate maturation steps, each $w_i(t)$ shows quasiperiodic behavior in response to $\beta(t)$. Specifically, $w_i(t)$ for $0 \leq i \leq n$ have the following form [1, 2]:

$$\begin{aligned} w_i(t) &= W_i(t)e^{\tilde{r}_0 t} \\ W_i(t) &= W_i(t+T) \end{aligned} \tag{S.53}$$

We can proceed further by substituting Equations (S.53) into Equation (S.40), which allows us to solve for the growth rate, $\tilde{r}_0$, of the viral strain when sampled at integer multiples of the drug-dosing period. Performing these substitutions, we obtain

$$\begin{pmatrix} \mathcal{M}_{00} & \mathcal{M}_{01} & \dots & \mathcal{M}_{0n} \\ \mathcal{M}_{10} & \mathcal{M}_{11} & & \vdots \\ \vdots & & \ddots & \vdots \\ \mathcal{M}_{n0} & \dots & \dots & \mathcal{M}_{nn} \end{pmatrix} \begin{pmatrix} w_0(L) \\ w_1(L) \\ \vdots \\ w_n(L) \end{pmatrix} = 0 \tag{S.54}$$

Equation (S.54) admits a nontrivial solution (i.e., a solution for which all $w_i(L)$ are nonzero) and a solution for $\tilde{r}_0$ if

$$\det\left(\mathcal{M}_{ij}\right) = 0 \tag{S.55}$$

The maximum value of $\tilde{r}_0$ for which Equation (S.55) is satisfied specifies the growth rate of the virus when each $w_i(t)$ is sampled at times that are integer multiples of the drug-dosing period, T. If $\tilde{r}_0 > 0$, then, when sampled at times that are integer multiples of the drug-dosing period, the density of infected cells increases in time, and infection is established. If $\tilde{r}_0 < 0$, then, when sampled at times that are integer multiples of the drug-dosing period, the density of infected cells decreases in time, and infection is eliminated.

Using what we have already derived, we can write $\mathcal{M}_{ij}$ for the simple cases of $n = 1$ and $n = 2$.

Particular case: $n = 1$

If $n = 1$, then the $\mathcal{M}_{ij}$ are given by

$$\begin{aligned} \mathcal{M}_{00} &= -e^{\tilde{r}_0 T} + e^{-\gamma_0 T}\left[1 + \xi_{-1}(-1)\xi_{-2}(-2) + \cdots\right] \\ \mathcal{M}_{01} &= e^{-\gamma_0 T}\left[\xi_{-1}(-1) + \xi_{-1}(-1)\xi_{-2}(-2)\xi_{-3}(-3) + \cdots\right] \\ \mathcal{M}_{10} &= e^{-\gamma_1 T}\left[\xi_{-1}(0) + \xi_{-1}(0)\xi_{-2}(-1)\xi_{-3}(-2) + \cdots\right] \\ \mathcal{M}_{11} &= -e^{\tilde{r}_0 T} + e^{-\gamma_1 T}\left[1 + \xi_{-1}(0)\xi_{-2}(-1) + \cdots\right] \end{aligned}$$

Particular case: $n = 2$

If $n = 2$, then the $\mathcal{M}_{ij}$ are given by

$$\begin{aligned}
\mathcal{M}_{00} &= -e^{\tilde{r}_0 T} + e^{-\gamma_0 T}\left[1 + \xi_{-1}(-1)\xi_{-2}(-2)\xi_{-3}(-3) + \cdots\right] \\
\mathcal{M}_{01} &= e^{-\gamma_0 T}\left[\xi_{-1}(-1)\xi_{-2}(-2) + \xi_{-1}(-1)\xi_{-2}(-2)\xi_{-3}(-3)\xi_{-4}(-4)\xi_{-5}(-5) + \cdots\right] \\
\mathcal{M}_{02} &= e^{-\gamma_0 T}\left[\xi_{-1}(-1) + \xi_{-1}(-1)\xi_{-2}(-2)\xi_{-3}(-3)\xi_{-4}(-4) + \cdots\right] \\
\mathcal{M}_{10} &= e^{-\gamma_1 T}\left[\xi_{-1}(0) + \xi_{-1}(0)\xi_{-2}(-1)\xi_{-3}(-2)\xi_{-4}(-3) + \cdots\right] \\
\mathcal{M}_{11} &= -e^{\tilde{r}_0 T} + e^{-\gamma_1 T}\left[1 + \xi_{-1}(0)\xi_{-2}(-1)\xi_{-3}(-2) + \cdots\right] \\
\mathcal{M}_{12} &= e^{-\gamma_1 T}\left[\xi_{-1}(0)\xi_{-2}(-1) + \xi_{-1}(0)\xi_{-2}(-1)\xi_{-3}(-2)\xi_{-4}(-3)\xi_{-5}(-4) + \cdots\right] \\
\mathcal{M}_{20} &= e^{-\gamma_2 T}\left[\xi_{-1}(1)\xi_{-2}(0) + \xi_{-1}(1)\xi_{-2}(0)\xi_{-3}(-1)\xi_{-4}(-2)\xi_{-5}(-3) + \cdots\right] \\
\mathcal{M}_{21} &= e^{-\gamma_2 T}\left[\xi_{-1}(1) + \xi_{-1}(1)\xi_{-2}(0)\xi_{-3}(-1)\xi_{-4}(-2) + \cdots\right] \\
\mathcal{M}_{22} &= -e^{\tilde{r}_0 T} + e^{-\gamma_2 T}\left[1 + \xi_{-1}(1)\xi_{-2}(0)\xi_{-3}(-1) + \cdots\right]
\end{aligned}$$

Case of any $n \geq 1$

For any $n \geq 1$, the $\mathcal{M}_{ij}$ are given by

$$\begin{aligned}
\mathcal{M}_{ij} = -\delta_{ij} e^{\tilde{r}_0 T} + e^{-\gamma_i T} &\left(\prod_{\ell=1}^{\Lambda(i-j,n)} \xi_{-\ell}(i-\ell)\right) \\
\times \sum_{p=0}^{\infty} \prod_{q=0}^{p-1} \prod_{k=1}^{n+1} &\xi_{-\Lambda(i-j,n)-(n+1)q-k}(i - \Lambda(i-j,n) - (n+1)q - k)
\end{aligned} \tag{S.56}$$

(Here, δ_{ij} is the Kronecker delta.) Just as for Equation (S.52), the integrals over $\beta(t)$ in Equation (S.56) can be done analytically, if possible, or can be performed using, e.g., Monte Carlo integration. (Please see the section on the derivation of $\tilde{r}_0$ for the case of no intermediate maturation steps for a description of how Monte Carlo integration works.)

With the same reasoning described in the previous section, we define a parameter, $\tilde{R}_0$, inspired by the basic reproductive ratio R_0 that can be calculated for the case of constant β (Eqs. (1),(4)):

$$\tilde{R}_0 \equiv \left(1 + \frac{\tilde{r}_0}{d_y}\right) \prod_{i=1}^{n} \left(1 + \frac{\tilde{r}_0}{m_i + d_i}\right) \qquad \text{for } n \text{ intermediate maturation steps} \tag{S.57}$$

6.3 The maturation process happens after a fixed time delay

If the number of intermediate maturation steps, n, is large, then the distribution of maturation times for newly infected cells becomes sharply peaked about the mean maturation time. Therefore, we suppose that an infected cell only begins producing new virions after a fixed maturation time, τ, has elapsed since its infection. We thus consider the viral dynamics specified by the following system of equations:

$$\begin{aligned}
\dot{x}(t) &= \lambda - \beta(t)y(t)x(t) - d_x x(t) \\
\dot{y}(t) &= \beta(t-\tau)y(t-\tau)x(t-\tau)e^{-d_w \tau} - d_y y(t)
\end{aligned}$$

We assume that the system starts from the uninfected equilibrium, and a small amount of the virus is introduced. If we focus on the early-time dynamics, when the density of infected cells is low, then the equation for $\dot{y}(t)$ becomes

$$\dot{y}(t) = \frac{\lambda}{d_x} e^{-d_w \tau} \beta(t-\tau) y(t-\tau) - d_y y(t) \tag{S.58}$$

Similarly to the analysis in the previous section, we can use an integrating factor on Equation (S.58) and then integrate this equation between time t and time t' to obtain

$$e^{d_y t'} y(t') = e^{d_y t} y(t) + \frac{\lambda}{d_x} e^{(d_y - d_w)\tau} \int_{t-\tau}^{t'-\tau} ds\, \beta(s) e^{d_y s} y(s) \tag{S.59}$$

(Here, t and t' are any early times in the dynamics such that the density of infected cells is low, so that Equation (S.58) holds.)

Before proceeding, it is helpful to explain our intuition for how to determine the evolutionary success of a viral strain with a fixed maturation time.

In the previous section, for n intermediate maturation steps, our aim was to solve for a linear operator, M_{ij}, that, when acting on the vector $w_i(L)$, produces the vector $w_i(L+T)$ (where recall that $w_0(t)$ is simply a convenient notation for $y(t)$). The largest eigenvalue of M_{ij} for a particular patient, viral strain, and drug-dosing regimen then determines if that viral strain establishes persistent infection or goes extinct. Such an approach is not directly applicable here because, for a fixed delay, τ, we are only considering the evolution of $y(t)$. However, notice that we also cannot use the simple procedure for the case with no intermediate maturation steps because Equation (S.59) contains an integration in which the upper and lower integration limits are both offset by an amount τ. We thus need a slightly different approach.

Physical intuition is helpful. Suppose that the system begins evolving according to Equation (S.58) at time $t = 0$. The initial data for this problem is given by specifying $\beta(t)$ and $y(t)$ between times $t = -\tau$ and $t = 0$. At times shortly after the evolution begins, the initial data will certainly affect the evolution of the density of infected cells. As the evolution progresses, the initial data will become less important, and eventually, the density of infected cells will settle into a quasiperiodic temporal pattern that is determined by the form of the periodic infectivity, $\beta(t)$. But at times that are too long after the evolution begins, the density of target cells can no longer be assumed to be at its uninfected level.

For performing a meaningful and tractable analytical analysis of this problem, we therefore want to focus on times that are sufficiently long after the evolution begins that the influence of the initial data on the temporal pattern of $y(t)$ has decayed away, yet we must still assume that times are sufficiently short that the density of target cells remains approximately at its uninfected level. Within this time regime, $y(t)$ has a similar form as was described in the previous two sections:

$$\begin{aligned} y(t) &= Y(t) e^{\tilde{r}_0 t} \\ Y(t) &= Y(t+T) \end{aligned} \tag{S.60}$$

To see how to simplify the analysis of Equations (S.59) and (S.60), it is helpful to begin by example.

Particular case: $\tau = T$

Let us first consider the case where $\tau = T$, which turns out to be relatively simple analytically. Consider Equation (S.59) with the following substitutions: $t' = L\tau + t_k$, $t = L\tau$, and $s = (L -$

$1)\tau + t_{k-1}$:

$$
\begin{aligned}
e^{d_y t_k} y(L\tau + t_k) = {} & y(L\tau) \\
& + \frac{\lambda}{d_x} e^{-d_w \tau} \int_0^{t_k} dt_{k-1}\, \beta((L-1)\tau + t_{k-1}) \\
& \times e^{d_y t_{k-1}} y((L-1)\tau + t_{k-1})
\end{aligned} \tag{S.61}
$$

Here, $L\tau$ and t_k are any times within the time regime of interest. The notation t_{k-1} for the integration variable is convenient, as it implies the manipulations that are to follow.

Now, let us set $k = 0$ and $t_0 = \tau$ in Equation (S.61); we have

$$
\begin{aligned}
e^{d_y \tau} y((L+1)\tau) = {} & y(L\tau) \\
& + \frac{\lambda}{d_x} e^{-d_w \tau} \int_0^{\tau} dt_{-1}\, \beta((L-1)\tau + t_{-1}) \\
& \times e^{d_y t_{-1}} y((L-1)\tau + t_{-1})
\end{aligned} \tag{S.62}
$$

Next, we can substitute Equation (S.61) into Equation (S.62); we have

$$
\begin{aligned}
e^{d_y \tau} y((L+1)\tau) = {} & y(L\tau) \\
& + y((L-1)\tau) \frac{\lambda}{d_x} e^{-d_w \tau} \int_0^{\tau} dt_{-1}\, \beta((L-1)\tau + t_{-1}) \\
& + \left(\frac{\lambda}{d_x} e^{-d_w \tau} \right)^2 \\
& \times \int_0^{\tau} dt_{-1}\, \beta((L-1)\tau + t_{-1}) \int_0^{t_{-1}} dt_{-2}\, \beta((L-2)\tau + t_{-2}) \\
& \times e^{d_y t_{-2}} y((L-2)\tau + t_{-2})
\end{aligned} \tag{S.63}
$$

Next, we can substitute Equation (S.61) into Equation (S.63); we have

$$
\begin{aligned}
e^{d_y \tau} y((L+1)\tau) = {} & y(L\tau) \\
& + y((L-1)\tau) \frac{\lambda}{d_x} e^{-d_w \tau} \int_0^{\tau} dt_{-1}\, \beta((L-1)\tau + t_{-1}) \\
& + y((L-2)\tau) \left(\frac{\lambda}{d_x} e^{-d_w \tau} \right)^2 \\
& \times \int_0^{\tau} dt_{-1}\, \beta((L-1)\tau + t_{-1}) \int_0^{t_{-1}} dt_{-2}\, \beta((L-2)\tau + t_{-2}) \\
& + \left(\frac{\lambda}{d_x} e^{-d_w \tau} \right)^3 \\
& \times \int_0^{\tau} dt_{-1}\, \beta((L-1)\tau + t_{-1}) \int_0^{t_{-1}} dt_{-2}\, \beta((L-2)\tau + t_{-2}) \\
& \times \int_0^{t_{-2}} dt_{-3}\, \beta((L-3)\tau + t_{-3}) e^{d_y t_{-3}} y((L-3)\tau + t_{-3})
\end{aligned} \tag{S.64}
$$

Next, we can substitute Equation (S.61) into Equation (S.64), etc. If we continue this process *ad infinitum*, then we obtain

$$\begin{aligned}e^{d_y\tau}y((L+1)\tau) &= y(L\tau)\\&\quad+\sum_{j=1}^{\infty}y((L-j)\tau)\left(\frac{\lambda}{d_x}e^{-d_w\tau}\right)^j\\&\quad\times\int_0^{\tau}dt_{-1}\;\beta((L-1)\tau+t_{-1})\prod_{k=2}^{j}\int_0^{t_{-k+1}}dt_{-k}\;\beta((L-k)\tau+t_{-k})\end{aligned}\tag{S.65}$$

Notice that we can use Equations (S.60) in Equation (S.65). With these substitutions, and since $\tau = T$, we have $Y((L+1)\tau) = Y(L\tau)$, $Y(L\tau) = Y((L-1)\tau)$, $Y((L-1)\tau) = Y((L-2)\tau)$, etc., and Equation (S.65) becomes

$$\mathcal{M}'_{00}Y((L+1)\tau) = 0\tag{S.66}$$

where

$$\begin{aligned}\mathcal{M}'_{00} &= -e^{(d_y+\tilde{r}_0)\tau}+1+\frac{\lambda}{d_x}e^{-(d_w+\tilde{r}_0)\tau}\int_0^{\tau}dt_{-1}\;\beta((L-1)\tau+t_{-1})\\&\quad+\left(\frac{\lambda}{d_x}e^{-(d_w+\tilde{r}_0)\tau}\right)^2\int_0^{\tau}dt_{-1}\;\beta((L-1)\tau+t_{-1})\int_0^{t_{-1}}dt_{-2}\;\beta((L-2)\tau+t_{-2})\\&\quad+\cdots\end{aligned}\tag{S.67}$$

Particular case: $2\tau = T$

Let us next consider the case where $2\tau = T$. We proceed similarly. Consider Equation (S.59) with the following substitutions: $t' = L\tau + t_k$, $t = L\tau$, and $s = (L-1)\tau + t_{k-1}$:

$$\begin{aligned}e^{d_y t_k}y(L\tau+t_k) &= y(L\tau)\\&\quad+\frac{\lambda}{d_x}e^{-d_w\tau}\int_0^{t_k}dt_{k-1}\;\beta((L-1)\tau+t_{k-1})\\&\quad\times e^{d_y t_{k-1}}y((L-1)\tau+t_{k-1})\end{aligned}\tag{S.68}$$

Also, consider Equation (S.59) with the following substitutions: $t' = (L+1)\tau + t_k$, $t = (L+1)\tau$, and $s = L\tau + t_{k-1}$:

$$\begin{aligned}e^{d_y t_k}y((L+1)\tau+t_k) &= y((L+1)\tau)\\&\quad+\frac{\lambda}{d_x}e^{-d_w\tau}\int_0^{t_k}dt_{k-1}\;\beta(L\tau+t_{k-1})\\&\quad\times e^{d_y t_{k-1}}y(L\tau+t_{k-1})\end{aligned}\tag{S.69}$$

Here, $L\tau$ and t_k are any times within the time regime of interest. The notation t_{k-1} for the integration variable is convenient, as it implies the manipulations that are to follow.

Now, let us set $k = 0$ and $t_0 = \tau$ in Equations (S.68) and (S.69); we have

$$\begin{aligned}
e^{d_y \tau} y((L+1)\tau) &= y(L\tau) \\
&\quad + \frac{\lambda}{d_x} e^{-d_w \tau} \int_0^\tau dt_{-1}\, \beta((L-1)\tau + t_{-1}) \\
&\quad \times e^{d_y t_{-1}} y((L-1)\tau + t_{-1}) \\
e^{d_y \tau} y((L+2)\tau) &= y((L+1)\tau) \\
&\quad + \frac{\lambda}{d_x} e^{-d_w \tau} \int_0^\tau dt_{-1}\, \beta(L\tau + t_{-1}) \\
&\quad \times e^{d_y t_{-1}} y(L\tau + t_{-1})
\end{aligned} \tag{S.70}$$

Next, we can substitute Equation (S.68) into Equations (S.70); we have

$$\begin{aligned}
e^{d_y \tau} y((L+1)\tau) &= y(L\tau) \\
&\quad + y((L-1)\tau) \frac{\lambda}{d_x} e^{-d_w \tau} \int_0^\tau dt_{-1}\, \beta((L-1)\tau + t_{-1}) \\
&\quad + \left(\frac{\lambda}{d_x} e^{-d_w \tau} \right)^2 \\
&\quad \times \int_0^\tau dt_{-1}\, \beta((L-1)\tau + t_{-1}) \int_0^{t_{-1}} dt_{-2}\, \beta((L-2)\tau + t_{-2}) \\
&\quad \times e^{d_y t_{-2}} y((L-2)\tau + t_{-2}) \\
e^{d_y \tau} y((L+2)\tau) &= y((L+1)\tau) \\
&\quad + y(L\tau) \frac{\lambda}{d_x} e^{-d_w \tau} \int_0^\tau dt_{-1}\, \beta(L\tau + t_{-1}) \\
&\quad + \left(\frac{\lambda}{d_x} e^{-d_w \tau} \right)^2 \\
&\quad \times \int_0^\tau dt_{-1}\, \beta(L\tau + t_{-1}) \int_0^{t_{-1}} dt_{-2}\, \beta((L-1)\tau + t_{-2}) \\
&\quad \times e^{d_y t_{-2}} y((L-1)\tau + t_{-2})
\end{aligned} \tag{S.71}$$

Next, we can substitute Equation (S.68) into Equations (S.71); we have

$$
\begin{aligned}
e^{d_y\tau}y((L+1)\tau) &= y(L\tau) \\
&+ y((L-1)\tau)\frac{\lambda}{d_x}e^{-d_w\tau}\int_0^{\tau} dt_{-1}\,\beta((L-1)\tau+t_{-1}) \\
&+ y((L-2)\tau)\left(\frac{\lambda}{d_x}e^{-d_w\tau}\right)^2 \\
&\times \int_0^{\tau} dt_{-1}\,\beta((L-1)\tau+t_{-1})\int_0^{t_{-1}} dt_{-2}\,\beta((L-2)\tau+t_{-2}) \\
&+ \left(\frac{\lambda}{d_x}e^{-d_w\tau}\right)^3 \\
&\times \int_0^{\tau} dt_{-1}\,\beta((L-1)\tau+t_{-1})\int_0^{t_{-1}} dt_{-2}\,\beta((L-2)\tau+t_{-2}) \\
&\times \int_0^{t_{-2}} dt_{-3}\,\beta((L-3)\tau+t_{-3})e^{d_y t_{-3}}y((L-3)\tau+t_{-3}) \\
e^{d_y\tau}y((L+2)\tau) &= y((L+1)\tau) \\
&+ y(L\tau)\frac{\lambda}{d_x}e^{-d_w\tau}\int_0^{\tau} dt_{-1}\,\beta(L\tau+t_{-1}) \\
&+ y((L-1)\tau)\left(\frac{\lambda}{d_x}e^{-d_w\tau}\right)^2 \\
&\times \int_0^{\tau} dt_{-1}\,\beta(L\tau+t_{-1})\int_0^{t_{-1}} dt_{-2}\,\beta((L-1)\tau+t_{-2}) \\
&+ \left(\frac{\lambda}{d_x}e^{-d_w\tau}\right)^3 \\
&\times \int_0^{\tau} dt_{-1}\,\beta(L\tau+t_{-1})\int_0^{t_{-1}} dt_{-2}\,\beta((L-1)\tau+t_{-2}) \\
&\times \int_0^{t_{-2}} dt_{-3}\,\beta((L-2)\tau+t_{-3})e^{d_y t_{-3}}y((L-2)\tau+t_{-3})
\end{aligned}
\tag{S.72}
$$

Next, we can substitute Equation (S.68) into Equations (S.72), etc. If we continue this process *ad*

infinitum, then we obtain

$$
\begin{aligned}
e^{d_y\tau} y((L+1)\tau) &= y(L\tau) \\
&+ \sum_{j=1}^{\infty} y((L-j)\tau) \left(\frac{\lambda}{d_x} e^{-d_w\tau}\right)^j \\
&\times \int_0^\tau dt_{-1}\, \beta((L-1)\tau + t_{-1}) \prod_{k=2}^{j} \int_0^{t_{-k+1}} dt_{-k}\, \beta((L-k)\tau + t_{-k}) \\
e^{d_y\tau} y((L+2)\tau) &= y((L+1)\tau) \\
&+ \sum_{j=1}^{\infty} y((L-j+1)\tau) \left(\frac{\lambda}{d_x} e^{-d_w\tau}\right)^j \\
&\times \int_0^\tau dt_{-1}\, \beta(L\tau + t_{-1}) \prod_{k=2}^{j} \int_0^{t_{-k+1}} dt_{-k}\, \beta((L-k+1)\tau + t_{-k})
\end{aligned}
\tag{S.73}
$$

Notice that we can use Equations (S.60) in Equations (S.73). With these substitutions, and since $2\tau = T$, we have $Y((L+2)\tau) = Y(L\tau)$, $Y((L+1)\tau) = Y((L-1)\tau)$, $Y(L\tau) = Y((L-2)\tau)$, etc., and Equations (S.73) become

$$
\begin{pmatrix} \mathcal{M}'_{00} & \mathcal{M}'_{01} \\ \mathcal{M}'_{10} & \mathcal{M}'_{11} \end{pmatrix} \begin{pmatrix} Y((L+1)\tau) \\ Y((L+2)\tau) \end{pmatrix} = 0 \tag{S.74}
$$

The $\mathcal{M}'_{ij}$ are given by

$$
\begin{aligned}
\mathcal{M}'_{00} &= -e^{(d_y+\tilde{r}_0)\tau} + \frac{\lambda}{d_x} e^{-(d_w+\tilde{r}_0)\tau} \int_0^\tau dt_{-1}\, \beta((L-1)\tau + t_{-1}) \\
&+ \left(\frac{\lambda}{d_x} e^{-(d_w+\tilde{r}_0)\tau}\right)^3 \int_0^\tau dt_{-1}\, \beta((L-1)\tau + t_{-1}) \int_0^{t_{-1}} dt_{-2}\, \beta((L-2)\tau + t_{-2}) \\
&\times \int_0^{t_{-2}} dt_{-3}\, \beta((L-3)\tau + t_{-3}) + \cdots \\
\mathcal{M}'_{01} &= 1 + \left(\frac{\lambda}{d_x} e^{-(d_w+\tilde{r}_0)\tau}\right)^2 \int_0^\tau dt_{-1}\, \beta((L-1)\tau + t_{-1}) \int_0^{t_{-1}} dt_{-2}\, \beta((L-2)\tau + t_{-2}) \\
&+ \cdots \\
\mathcal{M}'_{10} &= 1 + \left(\frac{\lambda}{d_x} e^{-(d_w+\tilde{r}_0)\tau}\right)^2 \int_0^\tau dt_{-1}\, \beta(L\tau + t_{-1}) \int_0^{t_{-1}} dt_{-2}\, \beta((L-1)\tau + t_{-2}) \\
&+ \cdots \\
\mathcal{M}'_{11} &= -e^{(d_y+\tilde{r}_0)\tau} + \frac{\lambda}{d_x} e^{-(d_w+\tilde{r}_0)\tau} \int_0^\tau dt_{-1}\, \beta(L\tau + t_{-1}) \\
&+ \left(\frac{\lambda}{d_x} e^{-(d_w+\tilde{r}_0)\tau}\right)^3 \int_0^\tau dt_{-1}\, \beta(L\tau + t_{-1}) \int_0^{t_{-1}} dt_{-2}\, \beta((L-1)\tau + t_{-2}) \\
&\times \int_0^{t_{-2}} dt_{-3}\, \beta((L-2)\tau + t_{-3}) + \cdots
\end{aligned}
\tag{S.75}
$$

Particular case: $3\tau = T$

Let us next consider the case where $3\tau = T$. We proceed similarly. Consider Equation (S.59) with the following substitutions: $t' = L\tau + t_k$, $t = L\tau$, and $s = (L-1)\tau + t_{k-1}$:

$$\begin{aligned} e^{d_y t_k} y(L\tau + t_k) = y(L\tau) \\ + \frac{\lambda}{d_x} e^{-d_w \tau} \int_0^{t_k} dt_{k-1}\, \beta((L-1)\tau + t_{k-1}) \\ \times e^{d_y t_{k-1}} y((L-1)\tau + t_{k-1}) \end{aligned} \tag{S.76}$$

Also, consider Equation (S.59) with the following substitutions: $t' = (L+1)\tau + t_k$, $t = (L+1)\tau$, and $s = L\tau + t_{k-1}$:

$$\begin{aligned} e^{d_y t_k} y((L+1)\tau + t_k) = y((L+1)\tau) \\ + \frac{\lambda}{d_x} e^{-d_w \tau} \int_0^{t_k} dt_{k-1}\, \beta(L\tau + t_{k-1}) \\ \times e^{d_y t_{k-1}} y(L\tau + t_{k-1}) \end{aligned} \tag{S.77}$$

In addition, consider Equation (S.59) with the following substitutions: $t' = (L+2)\tau + t_k$, $t = (L+2)\tau$, and $s = (L+1)\tau + t_{k-1}$:

$$\begin{aligned} e^{d_y t_k} y((L+2)\tau + t_k) = y((L+2)\tau) \\ + \frac{\lambda}{d_x} e^{-d_w \tau} \int_0^{t_k} dt_{k-1}\, \beta((L+1)\tau + t_{k-1}) \\ \times e^{d_y t_{k-1}} y((L+1)\tau + t_{k-1}) \end{aligned} \tag{S.78}$$

Here, $L\tau$ and t_k are any times within the time regime of interest. The notation t_{k-1} for the integration variable is convenient, as it implies the manipulations that are to follow.

Now, let us set $k = 0$ and $t_0 = \tau$ in Equations (S.76), (S.77), and (S.78); we have

$$\begin{aligned} e^{d_y \tau} y((L+1)\tau) = y(L\tau) \\ + \frac{\lambda}{d_x} e^{-d_w \tau} \int_0^{\tau} dt_{-1}\, \beta((L-1)\tau + t_{-1}) \\ \times e^{d_y t_{-1}} y((L-1)\tau + t_{-1}) \\ e^{d_y \tau} y((L+2)\tau) = y((L+1)\tau) \\ + \frac{\lambda}{d_x} e^{-d_w \tau} \int_0^{\tau} dt_{-1}\, \beta(L\tau + t_{-1}) \\ \times e^{d_y t_{-1}} y(L\tau + t_{-1}) \\ e^{d_y \tau} y((L+3)\tau) = y((L+2)\tau) \\ + \frac{\lambda}{d_x} e^{-d_w \tau} \int_0^{\tau} dt_{-1}\, \beta((L+1)\tau + t_{-1}) \\ \times e^{d_y t_{-1}} y((L+1)\tau + t_{-1}) \end{aligned} \tag{S.79}$$

Next, we can substitute Equation (S.76) into Equations (S.79); we have

$$\begin{aligned}
e^{d_y\tau}y((L+1)\tau) &= y(L\tau) \\
&+ y((L-1)\tau)\frac{\lambda}{d_x}e^{-d_w\tau}\int_0^\tau dt_{-1}\ \beta((L-1)\tau+t_{-1}) \\
&+ \left(\frac{\lambda}{d_x}e^{-d_w\tau}\right)^2 \\
&\times \int_0^\tau dt_{-1}\ \beta((L-1)\tau+t_{-1})\int_0^{t_{-1}} dt_{-2}\ \beta((L-2)\tau+t_{-2}) \\
&\times e^{d_y t_{-2}}y((L-2)\tau+t_{-2}) \\
e^{d_y\tau}y((L+2)\tau) &= y((L+1)\tau) \\
&+ y(L\tau)\frac{\lambda}{d_x}e^{-d_w\tau}\int_0^\tau dt_{-1}\ \beta(L\tau+t_{-1}) \\
&+ \left(\frac{\lambda}{d_x}e^{-d_w\tau}\right)^2 \\
&\times \int_0^\tau dt_{-1}\ \beta(L\tau+t_{-1})\int_0^{t_{-1}} dt_{-2}\ \beta((L-1)\tau+t_{-2}) \\
&\times e^{d_y t_{-2}}y((L-1)\tau+t_{-2}) \\
e^{d_y\tau}y((L+3)\tau) &= y((L+2)\tau) \\
&+ y((L+1)\tau)\frac{\lambda}{d_x}e^{-d_w\tau}\int_0^\tau dt_{-1}\ \beta((L+1)\tau+t_{-1}) \\
&+ \left(\frac{\lambda}{d_x}e^{-d_w\tau}\right)^2 \\
&\times \int_0^\tau dt_{-1}\ \beta((L+1)\tau+t_{-1})\int_0^{t_{-1}} dt_{-2}\ \beta(L\tau+t_{-2}) \\
&\times e^{d_y t_{-2}}y(L\tau+t_{-2})
\end{aligned} \tag{S.80}$$

Next, we can substitute Equation (S.76) into Equations (S.80); we have

$$
\begin{aligned}
e^{d_y\tau} y((L+1)\tau) &= y(L\tau) \\
&+ y((L-1)\tau)\frac{\lambda}{d_x}e^{-d_w\tau}\int_0^\tau dt_{-1}\ \beta((L-1)\tau+t_{-1}) \\
&+ y((L-2)\tau)\left(\frac{\lambda}{d_x}e^{-d_w\tau}\right)^2 \\
&\times \int_0^\tau dt_{-1}\ \beta((L-1)\tau+t_{-1})\int_0^{t_{-1}} dt_{-2}\ \beta((L-2)\tau+t_{-2}) \\
&+ \left(\frac{\lambda}{d_x}e^{-d_w\tau}\right)^3 \\
&\times \int_0^\tau dt_{-1}\ \beta((L-1)\tau+t_{-1})\int_0^{t_{-1}} dt_{-2}\ \beta((L-2)\tau+t_{-2}) \\
&\times \int_0^{t_{-2}} dt_{-3}\ \beta((L-3)\tau+t_{-3})e^{d_y t_{-3}}y((L-3)\tau+t_{-3}) \\
e^{d_y\tau} y((L+2)\tau) &= y((L+1)\tau) \\
&+ y(L\tau)\frac{\lambda}{d_x}e^{-d_w\tau}\int_0^\tau dt_{-1}\ \beta(L\tau+t_{-1}) \\
&+ y((L-1)\tau)\left(\frac{\lambda}{d_x}e^{-d_w\tau}\right)^2 \\
&\times \int_0^\tau dt_{-1}\ \beta(L\tau+t_{-1})\int_0^{t_{-1}} dt_{-2}\ \beta((L-1)\tau+t_{-2}) \\
&+ \left(\frac{\lambda}{d_x}e^{-d_w\tau}\right)^3 \\
&\times \int_0^\tau dt_{-1}\ \beta(L\tau+t_{-1})\int_0^{t_{-1}} dt_{-2}\ \beta((L-1)\tau+t_{-2}) \\
&\times \int_0^{t_{-2}} dt_{-3}\ \beta((L-2)\tau+t_{-3})e^{d_y t_{-3}}y((L-2)\tau+t_{-3}) \\
e^{d_y\tau} y((L+3)\tau) &= y((L+2)\tau) \\
&+ y((L+1)\tau)\frac{\lambda}{d_x}e^{-d_w\tau}\int_0^\tau dt_{-1}\ \beta((L+1)\tau+t_{-1}) \\
&+ y(L\tau)\left(\frac{\lambda}{d_x}e^{-d_w\tau}\right)^2 \\
&\times \int_0^\tau dt_{-1}\ \beta((L+1)\tau+t_{-1})\int_0^{t_{-1}} dt_{-2}\ \beta(L\tau+t_{-2}) \\
&+ \left(\frac{\lambda}{d_x}e^{-d_w\tau}\right)^3 \\
&\times \int_0^\tau dt_{-1}\ \beta((L+1)\tau+t_{-1})\int_0^{t_{-1}} dt_{-2}\ \beta(L\tau+t_{-2}) \\
&\times \int_0^{t_{-2}} dt_{-3}\ \beta((L-1)\tau+t_{-3})e^{d_y t_{-3}}y((L-1)\tau+t_{-3})
\end{aligned}
\tag{S.81}
$$

Next, we can substitute Equation (S.76) into Equations (S.81), etc. If we continue this process *ad infinitum*, then we obtain

$$
\begin{aligned}
e^{d_y\tau} y((L+1)\tau) &= y(L\tau) \\
&+ \sum_{j=1}^{\infty} y((L-j)\tau) \left(\frac{\lambda}{d_x} e^{-d_w\tau}\right)^j \\
&\times \int_0^{\tau} dt_{-1}\, \beta((L-1)\tau + t_{-1}) \prod_{k=2}^{j} \int_0^{t_{-k+1}} dt_{-k}\, \beta((L-k)\tau + t_{-k}) \\
e^{d_y\tau} y((L+2)\tau) &= y((L+1)\tau) \\
&+ \sum_{j=1}^{\infty} y((L-j+1)\tau) \left(\frac{\lambda}{d_x} e^{-d_w\tau}\right)^j \\
&\times \int_0^{\tau} dt_{-1}\, \beta(L\tau + t_{-1}) \prod_{k=2}^{j} \int_0^{t_{-k+1}} dt_{-k}\, \beta((L-k+1)\tau + t_{-k}) \\
e^{d_y\tau} y((L+3)\tau) &= y((L+2)\tau) \\
&+ \sum_{j=1}^{\infty} y((L-j+2)\tau) \left(\frac{\lambda}{d_x} e^{-d_w\tau}\right)^j \\
&\times \int_0^{\tau} dt_{-1}\, \beta((L+1)\tau + t_{-1}) \prod_{k=2}^{j} \int_0^{t_{-k+1}} dt_{-k}\, \beta((L-k+2)\tau + t_{-k})
\end{aligned}
\tag{S.82}
$$

Notice that we can use Equations (S.60) in Equations (S.82). With these substitutions, and since $3\tau = T$, we have $Y((L+3)\tau) = Y(L\tau)$, $Y((L+2)\tau) = Y((L-1)\tau)$, $Y((L+1)\tau) = Y((L-2)\tau)$, etc., and Equations (S.82) become

$$
\begin{pmatrix} \mathcal{M}'_{00} & \mathcal{M}'_{01} & \mathcal{M}'_{02} \\ \mathcal{M}'_{10} & \mathcal{M}'_{11} & \mathcal{M}'_{12} \\ \mathcal{M}'_{20} & \mathcal{M}'_{21} & \mathcal{M}'_{22} \end{pmatrix} \begin{pmatrix} Y((L+1)\tau) \\ Y((L+2)\tau) \\ Y((L+3)\tau) \end{pmatrix} = 0 \tag{S.83}
$$

The $\mathcal{M}'_{ij}$ are given by

$$
\begin{aligned}
\mathcal{M}'_{00} &= -e^{(d_y+\tilde{r}_0)\tau} + \left(\frac{\lambda}{d_x} e^{-(d_w+\tilde{r}_0)\tau}\right)^2 \\
&\quad \times \int_0^{\tau} dt_{-1}\, \beta((L-1)\tau + t_{-1}) \int_0^{t_{-1}} dt_{-2}\, \beta((L-2)\tau + t_{-2}) + \cdots \\
\mathcal{M}'_{01} &= \frac{\lambda}{d_x} e^{-(d_w+\tilde{r}_0)\tau} \int_0^{\tau} dt_{-1}\, \beta((L-1)\tau + t_{-1}) \\
&\quad + \left(\frac{\lambda}{d_x} e^{-(d_w+\tilde{r}_0)\tau}\right)^4 \int_0^{\tau} dt_{-1}\, \beta((L-1)\tau + t_{-1}) \int_0^{t_{-1}} dt_{-2}\, \beta((L-2)\tau + t_{-2}) \\
&\quad \times \int_0^{t_{-2}} dt_{-3}\, \beta((L-3)\tau + t_{-3}) \int_0^{t_{-3}} dt_{-4}\, \beta((L-4)\tau + t_{-4}) + \cdots \\
\mathcal{M}'_{02} &= 1 + \left(\frac{\lambda}{d_x} e^{-(d_w+\tilde{r}_0)\tau}\right)^3 \int_0^{\tau} dt_{-1}\, \beta((L-1)\tau + t_{-1}) \int_0^{t_{-1}} dt_{-2}\, \beta((L-2)\tau + t_{-2}) \\
&\quad \times \int_0^{t_{-2}} dt_{-3}\, \beta((L-3)\tau + t_{-3}) + \cdots \\
\mathcal{M}'_{10} &= 1 + \left(\frac{\lambda}{d_x} e^{-(d_w+\tilde{r}_0)\tau}\right)^3 \int_0^{\tau} dt_{-1}\, \beta(L\tau + t_{-1}) \int_0^{t_{-1}} dt_{-2}\, \beta((L-1)\tau + t_{-2}) \\
&\quad \times \int_0^{t_{-2}} dt_{-3}\, \beta((L-2)\tau + t_{-3}) + \cdots \\
\mathcal{M}'_{11} &= -e^{(d_y+\tilde{r}_0)\tau} + \left(\frac{\lambda}{d_x} e^{-(d_w+\tilde{r}_0)\tau}\right)^2 \\
&\quad \times \int_0^{\tau} dt_{-1}\, \beta(L\tau + t_{-1}) \int_0^{t_{-1}} dt_{-2}\, \beta((L-1)\tau + t_{-2}) + \cdots \qquad \text{(S.84)} \\
\mathcal{M}'_{12} &= \frac{\lambda}{d_x} e^{-(d_w+\tilde{r}_0)\tau} \int_0^{\tau} dt_{-1}\, \beta(L\tau + t_{-1}) \\
&\quad + \left(\frac{\lambda}{d_x} e^{-(d_w+\tilde{r}_0)\tau}\right)^4 \int_0^{\tau} dt_{-1}\, \beta(L\tau + t_{-1}) \int_0^{t_{-1}} dt_{-2}\, \beta((L-1)\tau + t_{-2}) \\
&\quad \times \int_0^{t_{-2}} dt_{-3}\, \beta((L-2)\tau + t_{-3}) \int_0^{t_{-3}} dt_{-4}\, \beta((L-3)\tau + t_{-4}) + \cdots \\
\mathcal{M}'_{20} &= \frac{\lambda}{d_x} e^{-(d_w+\tilde{r}_0)\tau} \int_0^{\tau} dt_{-1}\, \beta((L+1)\tau + t_{-1}) \\
&\quad + \left(\frac{\lambda}{d_x} e^{-(d_w+\tilde{r}_0)\tau}\right)^4 \int_0^{\tau} dt_{-1}\, \beta((L+1)\tau + t_{-1}) \int_0^{t_{-1}} dt_{-2}\, \beta(L\tau + t_{-2}) \\
&\quad \times \int_0^{t_{-2}} dt_{-3}\, \beta((L-1)\tau + t_{-3}) \int_0^{t_{-3}} dt_{-4}\, \beta((L-2)\tau + t_{-4}) + \cdots \\
\mathcal{M}'_{21} &= 1 + \left(\frac{\lambda}{d_x} e^{-(d_w+\tilde{r}_0)\tau}\right)^3 \int_0^{\tau} dt_{-1}\, \beta((L+1)\tau + t_{-1}) \int_0^{t_{-1}} dt_{-2}\, \beta(L\tau + t_{-2}) \\
&\quad \times \int_0^{t_{-2}} dt_{-3}\, \beta((L-1)\tau + t_{-3}) + \cdots \\
\mathcal{M}'_{22} &= -e^{(d_y+\tilde{r}_0)\tau} + \left(\frac{\lambda}{d_x} e^{-(d_w+\tilde{r}_0)\tau}\right)^2 \\
&\quad \times \int_0^{\tau} dt_{-1}\, \beta((L+1)\tau + t_{-1}) \int_0^{t_{-1}} dt_{-2}\, \beta(L\tau + t_{-2}) + \cdots
\end{aligned}
$$

Case of any τ

Notice from above that regardless of the particular value of τ, we obtain a solution for $\tilde{r}_0$ by solving

$$\det\left(\mathcal{M}'_{ij}\right) = 0$$

Also notice that the dimensionality of the matrix $\mathcal{M}'_{ij}$ depends on the value of the maturation time, τ, in relation to the drug-dosing period, T. We therefore denote the dimensionality of the matrix $\mathcal{M}'_{ij}$ as $U(\tau, T)$.

Let us again consider the simple cases described above. If $\tau = T$, then the dimensionality of $\mathcal{M}'_{ij}$ is equal to 1. If $2\tau = T$, then the dimensionality of $\mathcal{M}'_{ij}$ is equal to 2. If $3\tau = T$, then the dimensionality of $\mathcal{M}'_{ij}$ is equal to 3. In each of these cases, we have that $U(\tau, T)$ is given by the lowest positive integer such that $\tau U(\tau, T)$ is equal to an integer multiple of T. This holds generally. For example, if $2\tau = 3T$, then the dimensionality of $\mathcal{M}'_{ij}$ is equal to 2. As another example, if $3\tau = 2T$, then the dimensionality of $\mathcal{M}'_{ij}$ is equal to 3.

Following the procedure described above, for any value of τ, we obtain

$$\begin{pmatrix} \mathcal{M}'_{00} & \mathcal{M}'_{01} & \cdots & \mathcal{M}'_{0,U(\tau,T)-1} \\ \mathcal{M}'_{10} & \mathcal{M}'_{11} & & \vdots \\ \vdots & & \ddots & \vdots \\ \mathcal{M}'_{U(\tau,T)-1,0} & \cdots & \cdots & \mathcal{M}'_{U(\tau,T)-1,U(\tau,T)-1} \end{pmatrix} \begin{pmatrix} Y((L+1)\tau) \\ Y((L+2)\tau) \\ \vdots \\ Y((L+U(\tau,T))\tau) \end{pmatrix} = 0 \tag{S.85}$$

Although the procedure outlined above shows us how to solve for $\mathcal{M}'_{ij}$, it is evident that the mathematical expressions for $\mathcal{M}'_{ij}$ are symbolically quite cumbersome. Therefore, we simplify notation substantially by making several definitions.

First, rather than writing $Y((L+1)\tau)$, $Y((L+2)\tau)$, $Y((L+3)\tau)$, etc., we can more simply write Y_{L+0}, Y_{L+1}, Y_{L+2}, etc. Therefore, we define

$$Y_{L+q} \equiv Y((L+q+1)\tau)$$

Next, since only the Y values Y_{L+0}, Y_{L+1}, ..., $Y_{U(\tau,T)-2}$, $Y_{U(\tau,T)-1}$ appear in Equations (S.85), it is notationally useful to have a function, $\Lambda'(q, \tau, T)$, that takes an integer, q, along with τ and T as inputs and that outputs an integer between 0 and $U(\tau, T) - 1$. We therefore define

$$\Lambda'(q, \tau, T) \equiv (q-1) \bmod U(\tau, T)$$

We would also like to simplify the notation for the integrations appearing in the infinite series in the solution for $\mathcal{M}'_{ij}$. To this end, consider Equation (S.59) with the following substitutions: $t' = (L+q)\tau + t_k$, $t = (L+q)\tau$, and $s = (L+q-1)\tau + t_{k-1}$:

$$\begin{aligned} e^{d_y t_k} y((L+q)\tau + t_k) = {} & y((L+q)\tau) \\ & + \frac{\lambda}{d_x} e^{-d_w \tau} \int_0^{t_k} dt_{k-1}\, \beta((L+q-1)\tau + t_{k-1}) \\ & \times e^{d_y t_{k-1}} y((L+q-1)\tau + t_{k-1}) \end{aligned} \tag{S.86}$$

Here, $L\tau$ and t_k are any times within the time regime of interest. Since we will be using the function $\Lambda'(q, \tau, T)$ to simplify the writing, q can be any integer in Equation (S.86). The notation t_{k-1} for the integration variable is convenient, as it implies the manipulations that are to follow.

Since each $\mathcal{M}'_{ij}$ is an infinite series in which factors appear in terms in cyclical fashion, we strive to simplify notation in the series expansion of each $\mathcal{M}'_{ij}$ to the maximum extent possible. We therefore make the following definitions, whose notational utility will become apparent:

$$\xi'_i(q, t_0) \equiv \frac{\lambda}{d_x} e^{-d_w \tau} \times \begin{cases} \int_0^{t_0} dt_{-1}\, \beta((L+q)\tau + t_{-1}), & \text{if } i = -1 \\ \int_0^{t_{i+1}} dt_i\, \beta((L+q)\tau + t_i), & \text{if } i < -1 \end{cases}$$

$$F'_q(t_k) \equiv e^{d_y t_k} y((L+q)\tau + t_k)$$

Now, with the preceding definitions, notice that Equations (S.86) can be written very compactly:

$$F'_q(t_k) = F'_q(0) + \xi'_{k-1}(q-1, t_k) F'_{q-1}(t_{k-1}) \tag{S.87}$$

In Equations (S.87), $k \leq 0$, and q can be any integer.

Also, notice that in Equation (S.65) for $U(\tau, T) = 1$, in Equations (S.73) for $U(\tau, T) = 2$, and in Equations (S.82) for $U(\tau, T) = 3$, each integral over t_{-1} has an upper limit of $t_{-1} = \tau$. Therefore, it simplifies notation further if we define

$$\xi'_i(q) \equiv \xi'_i(q, \tau)$$

Setting $k = 0$ and $t_0 = \tau$ in Equation (S.87), we have

$$F'_q(\tau) = F'_q(0) + \xi'_{-1}(q-1) F'_{q-1}(t_{-1}) \tag{S.88}$$

We then perform the repeated substitution of Equation (S.87) into Equation (S.88):

$$F'_q(\tau) = F'_q(0) + \sum_{p=1}^{\infty} F'_{q-p}(0) \prod_{k=1}^{p} \xi'_{-k}(q-k) \tag{S.89}$$

Again invoking our intuition that $y(t)$ is quasiperiodic in the time regime of interest, we substitute Equations (S.60) in Equation (S.89). Notice that Equation (S.89) with the substitutions of Equations (S.60) then yields $U(\tau, T)$ equations. Considering integer values of q between 0 and $U(\tau, T) - 1$ in Equation (S.89) with the substitutions of Equations (S.60), we obtain

$$\begin{aligned} e^{(d_y + \tilde{r}_0)\tau} Y_{L+\Lambda'(q+1,\tau,T)} &= Y_{L+\Lambda'(q,\tau,T)} \\ &\quad + \sum_{p=1}^{\infty} e^{-p\tilde{r}_0 \tau} Y_{L+\Lambda'(q-p,\tau,T)} \prod_{k=1}^{p} \xi'_{-k}(q-k) \end{aligned} \tag{S.90}$$

Let us now revisit our calculations for the simple cases of $\tau = T$, $2\tau = T$, and $3\tau = T$. More generally, since the respective mathematical steps are identical, let us consider the calculations for the simple cases of $U(\tau, T) = 1$, $U(\tau, T) = 2$, and $U(\tau, T) = 3$ to see how the notation with the above substitutions is dramatically simplified.

Particular case: $U(\tau, T) = 1$

For the case $U(\tau, T) = 1$, recall that the function $\Lambda'(q, \tau, T)$ reduces to 0. Therefore, with the definitions above, Equation (S.90) reduces to the following equation:

$$\mathcal{M}'_{00} Y_{L+0} = 0 \tag{S.91}$$

where

$$\mathcal{M}'_{00} = -e^{(d_y+\tilde{r}_0)\tau} + 1 + e^{-\tilde{r}_0\tau}\xi'_{-1}(-1) + e^{-2\tilde{r}_0\tau}\xi'_{-1}(-1)\xi'_{-2}(-2) + \cdots \tag{S.92}$$

It can be verified by direct substitution that Equations (S.91) and (S.92) reduce to Equations (S.66) and (S.67), respectively, for the case $\tau = T$. However, the notational and conceptual utility of the substitutions described above for simplifying the writing is evident.

Particular case: $U(\tau, T) = 2$

For the case $U(\tau, T) = 2$, recall that the function $\Lambda'(q, \tau, T)$ reduces to 0 or 1. Therefore, with the definitions above, Equation (S.90) reduces to the following two equations:

$$\begin{pmatrix} \mathcal{M}'_{00} & \mathcal{M}'_{01} \\ \mathcal{M}'_{10} & \mathcal{M}'_{11} \end{pmatrix} \begin{pmatrix} Y_{L+0} \\ Y_{L+1} \end{pmatrix} = 0 \tag{S.93}$$

where

$$\begin{aligned} \mathcal{M}'_{00} &= -e^{(d_y+\tilde{r}_0)\tau} + e^{-\tilde{r}_0\tau}\xi'_{-1}(-1) + e^{-3\tilde{r}_0\tau}\xi'_{-1}(-1)\xi'_{-2}(-2)\xi'_{-3}(-3) + \cdots \\ \mathcal{M}'_{01} &= 1 + e^{-2\tilde{r}_0\tau}\xi'_{-1}(-1)\xi'_{-2}(-2) + \cdots \\ \mathcal{M}'_{10} &= 1 + e^{-2\tilde{r}_0\tau}\xi'_{-1}(0)\xi'_{-2}(-1) + \cdots \\ \mathcal{M}'_{11} &= -e^{(d_y+\tilde{r}_0)\tau} + e^{-\tilde{r}_0\tau}\xi'_{-1}(0) + e^{-3\tilde{r}_0\tau}\xi'_{-1}(0)\xi'_{-2}(-1)\xi'_{-3}(-2) + \cdots \end{aligned} \tag{S.94}$$

It can be verified by direct substitution that Equations (S.93) and (S.94) reduce to Equations (S.74) and (S.75), respectively, for the case $2\tau = T$. However, the notational and conceptual utility of the substitutions described above for simplifying the writing is evident.

Particular case: $U(\tau, T) = 3$

For the case $U(\tau, T) = 3$, recall that the function $\Lambda'(q, \tau, T)$ reduces to 0, 1, or 2. Therefore, with the definitions above, Equation (S.90) reduces to the following three equations:

$$\begin{pmatrix} \mathcal{M}'_{00} & \mathcal{M}'_{01} & \mathcal{M}'_{02} \\ \mathcal{M}'_{10} & \mathcal{M}'_{11} & \mathcal{M}'_{12} \\ \mathcal{M}'_{20} & \mathcal{M}'_{21} & \mathcal{M}'_{22} \end{pmatrix} \begin{pmatrix} Y_{L+0} \\ Y_{L+1} \\ Y_{L+2} \end{pmatrix} = 0 \tag{S.95}$$

where

$$\begin{aligned} \mathcal{M}'_{00} &= -e^{(d_y+\tilde{r}_0)\tau} + e^{-2\tilde{r}_0\tau}\xi'_{-1}(-1)\xi'_{-2}(-2) \\ &\quad + e^{-5\tilde{r}_0\tau}\xi'_{-1}(-1)\xi'_{-2}(-2)\xi'_{-3}(-3)\xi'_{-4}(-4)\xi'_{-5}(-5) + \cdots \\ \mathcal{M}'_{01} &= e^{-\tilde{r}_0\tau}\xi'_{-1}(-1) + e^{-4\tilde{r}_0\tau}\xi'_{-1}(-1)\xi'_{-2}(-2)\xi'_{-3}(-3)\xi'_{-4}(-4) + \cdots \\ \mathcal{M}'_{02} &= 1 + e^{-3\tilde{r}_0\tau}\xi'_{-1}(-1)\xi'_{-2}(-2)\xi'_{-3}(-3) + \cdots \\ \mathcal{M}'_{10} &= 1 + e^{-3\tilde{r}_0\tau}\xi'_{-1}(0)\xi'_{-2}(-1)\xi'_{-3}(-2) + \cdots \\ \mathcal{M}'_{11} &= -e^{(d_y+\tilde{r}_0)\tau} + e^{-2\tilde{r}_0\tau}\xi'_{-1}(0)\xi'_{-2}(-1) \\ &\quad + e^{-5\tilde{r}_0\tau}\xi'_{-1}(0)\xi'_{-2}(-1)\xi'_{-3}(-2)\xi'_{-4}(-3)\xi'_{-5}(-4) + \cdots \\ \mathcal{M}'_{12} &= e^{-\tilde{r}_0\tau}\xi'_{-1}(0) + e^{-4\tilde{r}_0\tau}\xi'_{-1}(0)\xi'_{-2}(-1)\xi'_{-3}(-2)\xi'_{-4}(-3) + \cdots \\ \mathcal{M}'_{20} &= e^{-\tilde{r}_0\tau}\xi'_{-1}(1) + e^{-4\tilde{r}_0\tau}\xi'_{-1}(1)\xi'_{-2}(0)\xi'_{-3}(-1)\xi'_{-4}(-2) + \cdots \\ \mathcal{M}'_{21} &= 1 + e^{-3\tilde{r}_0\tau}\xi'_{-1}(1)\xi'_{-2}(0)\xi'_{-3}(-1) + \cdots \\ \mathcal{M}'_{22} &= -e^{(d_y+\tilde{r}_0)\tau} + e^{-2\tilde{r}_0\tau}\xi'_{-1}(1)\xi'_{-2}(0) \\ &\quad + e^{-5\tilde{r}_0\tau}\xi'_{-1}(1)\xi'_{-2}(0)\xi'_{-3}(-1)\xi'_{-4}(-2)\xi'_{-5}(-3) + \cdots \end{aligned} \tag{S.96}$$

It can be verified by direct substitution that Equations (S.95) and (S.96) reduce to Equations (S.83) and (S.84), respectively, for the case $3\tau = T$. However, the notational and conceptual utility of the substitutions described above for simplifying the writing is evident.

Solution for any $U(\tau, T) \geq 1$

For any $U(\tau, T) \geq 1$, we follow the same steps to obtain $U(\tau, T)$ equations:

$$\begin{pmatrix} \mathcal{M}'_{00} & \mathcal{M}'_{01} & \cdots & \mathcal{M}'_{0,U(\tau,T)-1} \\ \mathcal{M}'_{10} & \mathcal{M}'_{11} & & \vdots \\ \vdots & & \ddots & \vdots \\ \mathcal{M}'_{U(\tau,T)-1,0} & \cdots & \cdots & \mathcal{M}'_{U(\tau,T)-1,U(\tau,T)-1} \end{pmatrix} \begin{pmatrix} Y_{L+0} \\ Y_{L+1} \\ \vdots \\ Y_{L+U(\tau,T)-1} \end{pmatrix} = 0 \tag{S.97}$$

The $\mathcal{M}'_{ij}$ are given by

$$\begin{aligned} \mathcal{M}'_{ij} = -\delta_{ij} e^{(d_y+\tilde{r}_0)\tau} + e^{-\Lambda'(i-j,\tau,T)\tilde{r}_0\tau} \left(\prod_{\ell=1}^{\Lambda'(i-j,\tau,T)} \xi'_{-\ell}(i-\ell) \right) \\ \times \sum_{p=0}^{\infty} e^{-pU(\tau,T)\tilde{r}_0\tau} \\ \times \prod_{q=0}^{p-1} \prod_{k=1}^{U(\tau,T)} \xi'_{-\Lambda'(i-j,\tau,T)-U(\tau,T)q-k}(i - \Lambda'(i-j,\tau,T) - U(\tau,T)q - k) \end{aligned} \tag{S.98}$$

(Here, δ_{ij} is the Kronecker delta.) The integrals over $\beta(t)$ in Equation (S.98) can be done analytically, if possible, or can be performed using, e.g., Monte Carlo integration. (Please see the section on the derivation of $\tilde{r}_0$ for the case of no intermediate maturation steps for a description of how Monte Carlo integration works.)

Equation (S.97) admits a nontrivial solution and a solution for $\tilde{r}_0$ if

$$\det\left(\mathcal{M}'_{ij}\right) = 0 \tag{S.99}$$

In S15 Fig, for two different values of τ, we plot the infection level versus time obtained from simulations of the dynamics. In both cases, we also show the growth rate, $\tilde{r}_0$, of the infection level as predicted from Equation (S.99) when the infection level is sampled at integer multiples of the drug-dosing period, T. We find excellent agreement between theory and simulation.

With the same reasoning described in the previous two sections, we define a parameter, $\tilde{R}_0$, inspired by the basic reproductive ratio R_0 that can be calculated for the case of constant β (Eqs. (3),(5)):

$$\tilde{R}_0 \equiv \left(1 + \frac{\tilde{r}_0}{d_y}\right) e^{\tilde{r}_0\tau} \qquad \text{for a fixed time delay, } \tau \tag{S.100}$$

References

[1] Hale JK. Ordinary Differential Equations. Vol. XXI, Pure and applied mathematics. Wiley-Interscience, New York; 1969.

[2] Chicone C. Ordinary Differential Equations with Applications. Volume 34, Texts in Applied Mathematics. Springer, New York; 1999.

[3] Simmendinger C, Wunderlin A. Analytical approach for the Floquet theory of delay differential equations. Physical Review E. 1999; p. 5344–5353. doi:10.1103/PhysRevE.59.5344.

[4] Tian JP, Wang J. Some results in Floquet theory, with application to periodic epidemic models. Applicable Analysis. 2014; p. 5344–5353. doi:10.1080/00036811.2014.918606.

[5] Mitchell C, Kribs C. A Comparison of Methods for Calculating the Basic Reproductive Number for Periodic Epidemic Systems. Bulletin of Mathematical Biology. 2017;79(8):1846–1869. doi:10.1007/s11538-017-0309-y.

[6] Zhao XQ. Basic Reproduction Ratios for Periodic Compartmental Models with Time Delay. Journal of Dynamics and Differential Equations. 2017; p. 67–82. doi:10.1007/s10884-015-9425-2.

[7] Caflisch RE. Monte Carlo and quasi-Monte Carlo methods. Acta Numerica, Cambridge University Press. 1998; p. 1–49. doi:10.1017/S0962492900002804.

[8] Newman MEJ, Barkema GT. Monte Carlo Methods in Statistical Physics. Oxford University Press, Oxford; 1999.

[9] Nowak MA, May RM. Virus dynamics: Mathematical principles of immunology and virology. Oxford University Press; 2000.
