# Supplementary figures and images for: Life cycle synchronization is a viral drug resistance mechanism
Source: PLoS Comput Biol. 2018 Feb 15;14(2):e1005947. doi: 10.1371/journal.pcbi.1005947 (PMC5813899; doi:10.1371/journal.pcbi.1005947)

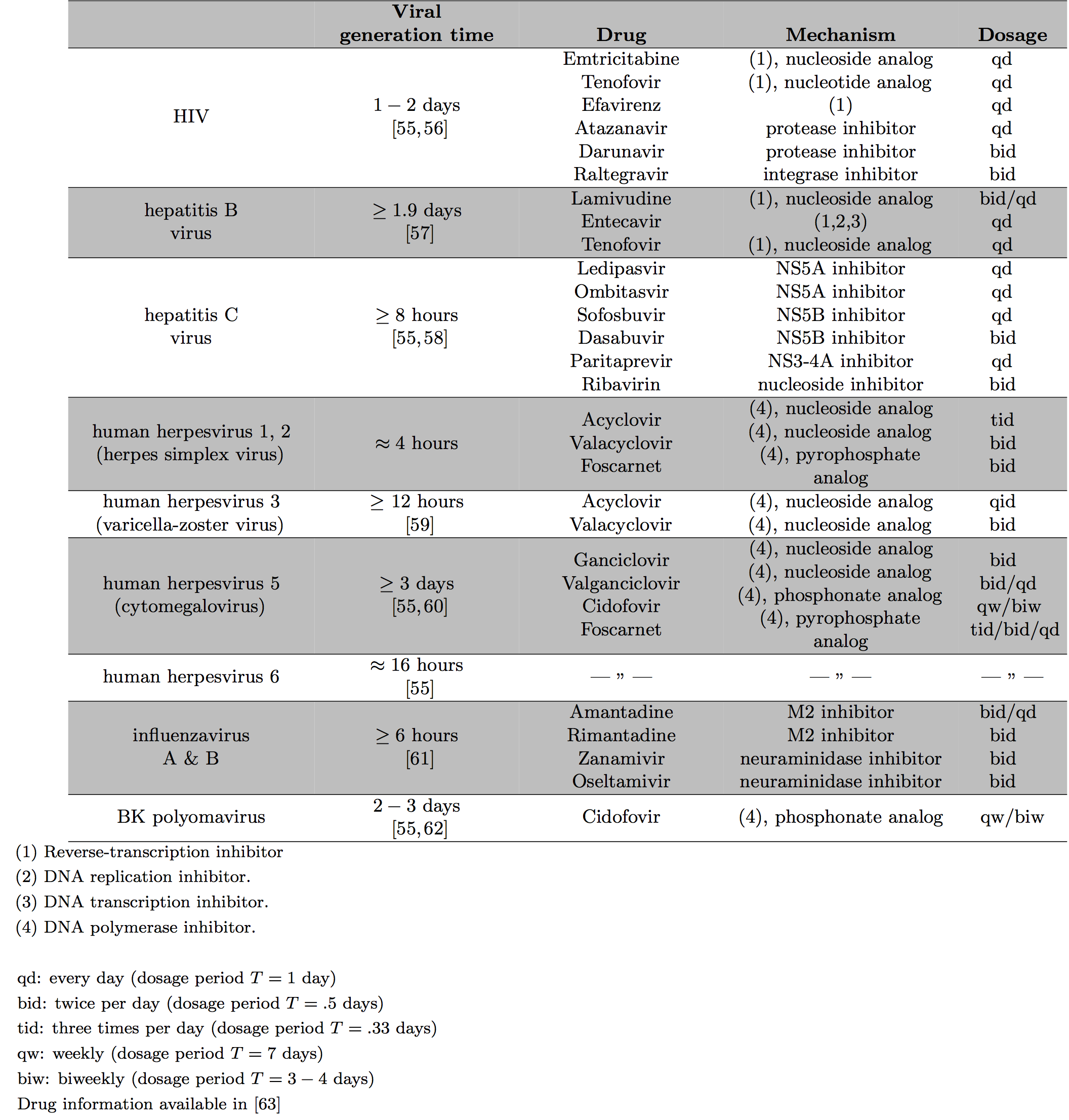

Supplement: S1 Table — (TIFF) [file pcbi.1005947.s002.tiff]

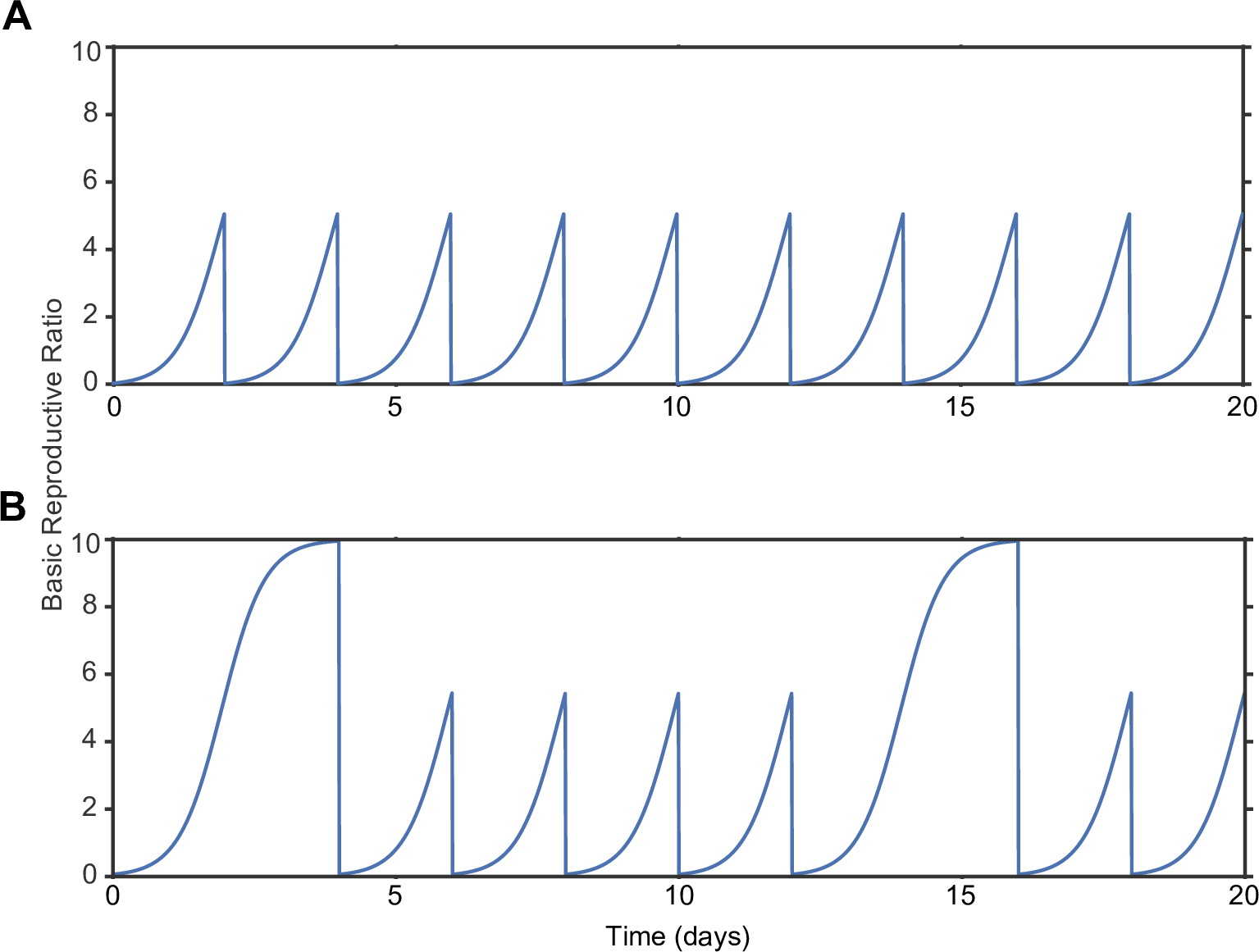

Supplement: S1 Fig — Fitness of the viral strain (measured by the basic reproductive ratio R0) fluctuates in response to drug levels approximated by the simple pharmacological model, Eq (7). (A) Perfect adherence to treatment. (B) Imperfect adherence to treatment: each dose is missed with a 30% probability. The model parameters used are drug period T = 2 days, drug-free R0 = 10, drug IC50 = 0.05 and slope (M) = 1.0, maximal drug level Cmax = 8.0, minimum drug level when all doses are taken Cmin = 0.04, dose size Δ = Cmax − Cmin = 7.96, and drug half-life th chosen such that the time-averaged drug efficacy is f = 0.85 (th = 0.25 days). (TIF) [file pcbi.1005947.s003.tif]

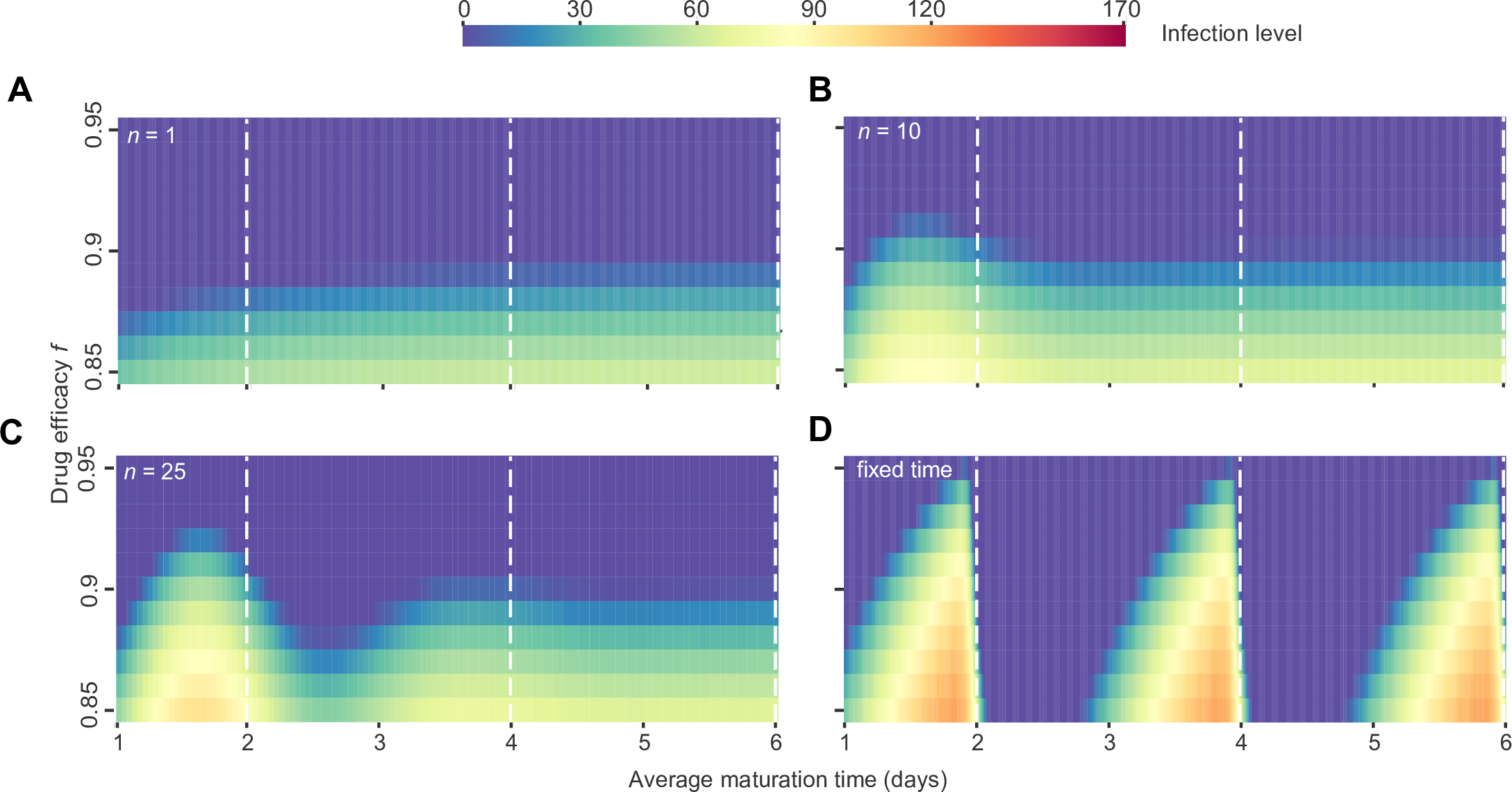

Supplement: S2 Fig — Viral dynamics were simulated under periodic antiviral therapy given by the simple on-off model with a period (T) of 2 days and varying drug efficacy (f). The infection level (heat map color) is measured as the concentration of mature infected cells (y) once a steady-state has been reached. Each calculation included only a single virus strain with average maturation time 1/m (maturation rate of nm for each stage). (A) Results with n = 1 maturation step. (B) Results with n = 10 maturation steps. (C) Results with n = 25 maturation steps. (D) Results with fixed maturation time τ = 1/m. The white dotted lines show where the average maturation time is equal to an integer multiple of the drug period. For all shown simulations, we assume the death rate of immature cells to be zero (dw = 0). Results shown for 41 different drug efficacies between f = 0.85 and f = 0.95, for 41 different strains with average maturation times between 1 and 6 days. (TIF) [file pcbi.1005947.s004.tif]

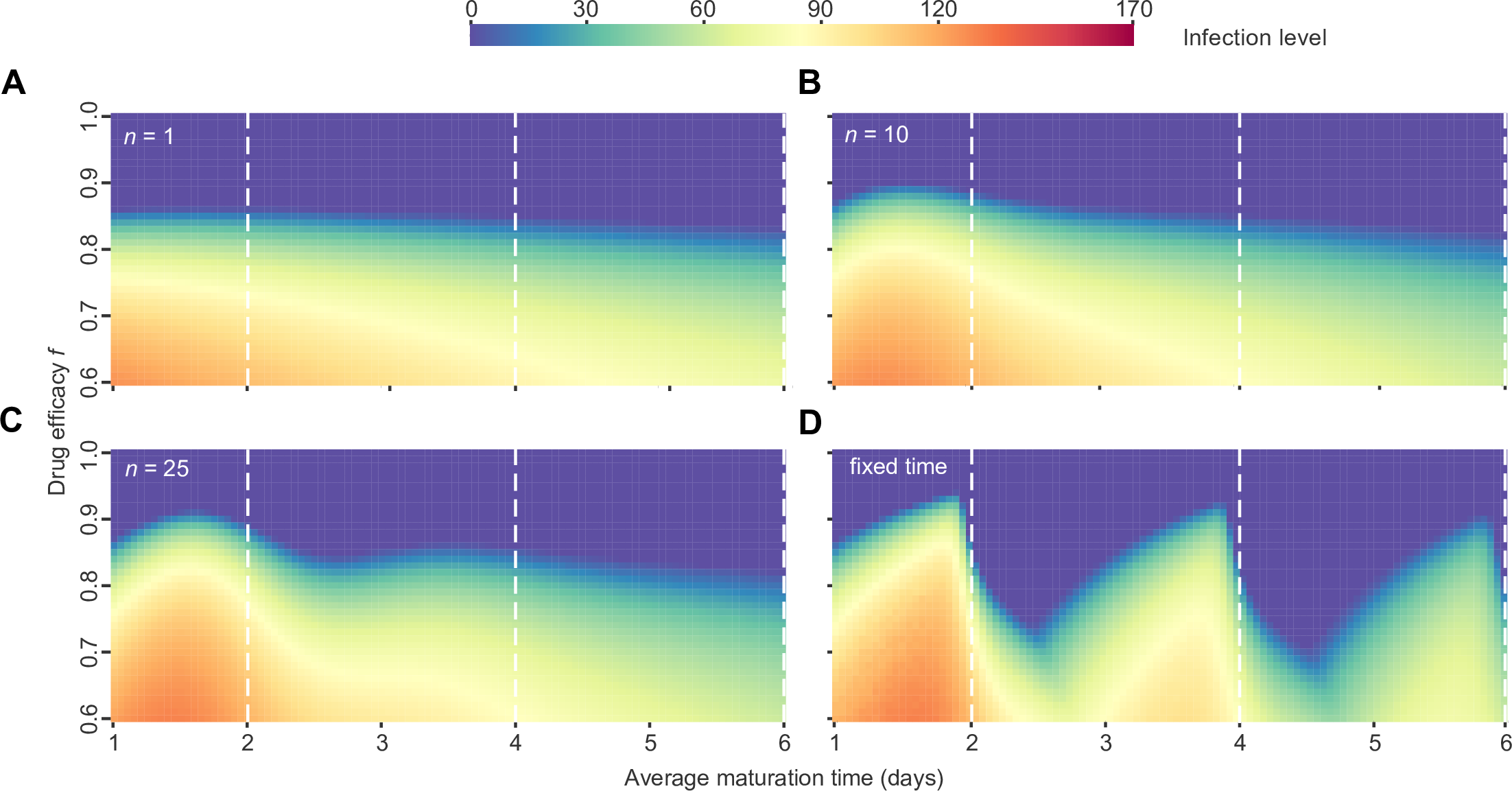

Supplement: S3 Fig — Viral dynamics were simulated under periodic antiviral therapy given by the simple on-off model with a period (T) of 2 days and varying drug efficacy (f). The infection level (heat map color) is measured as the concentration of mature infected cells (y) once a steady-state has been reached. Each calculation included only a single virus strain with average maturation time 1/m (maturation rate of nm for each stage). (A) Results with n = 1 maturation step. (B) Results with n = 10 maturation steps. (c) Results with n = 25 maturation steps. (d) Results with fixed maturation time τ = 1/m. For all shown simulations, we set the death rate of immature cells to dw = 0.1. Data shown for 41 different drug efficacies between f = 0.6 and f = 1.0, for 101 different strains with average maturation times between 1 and 6 days. (TIF) [file pcbi.1005947.s005.tif]

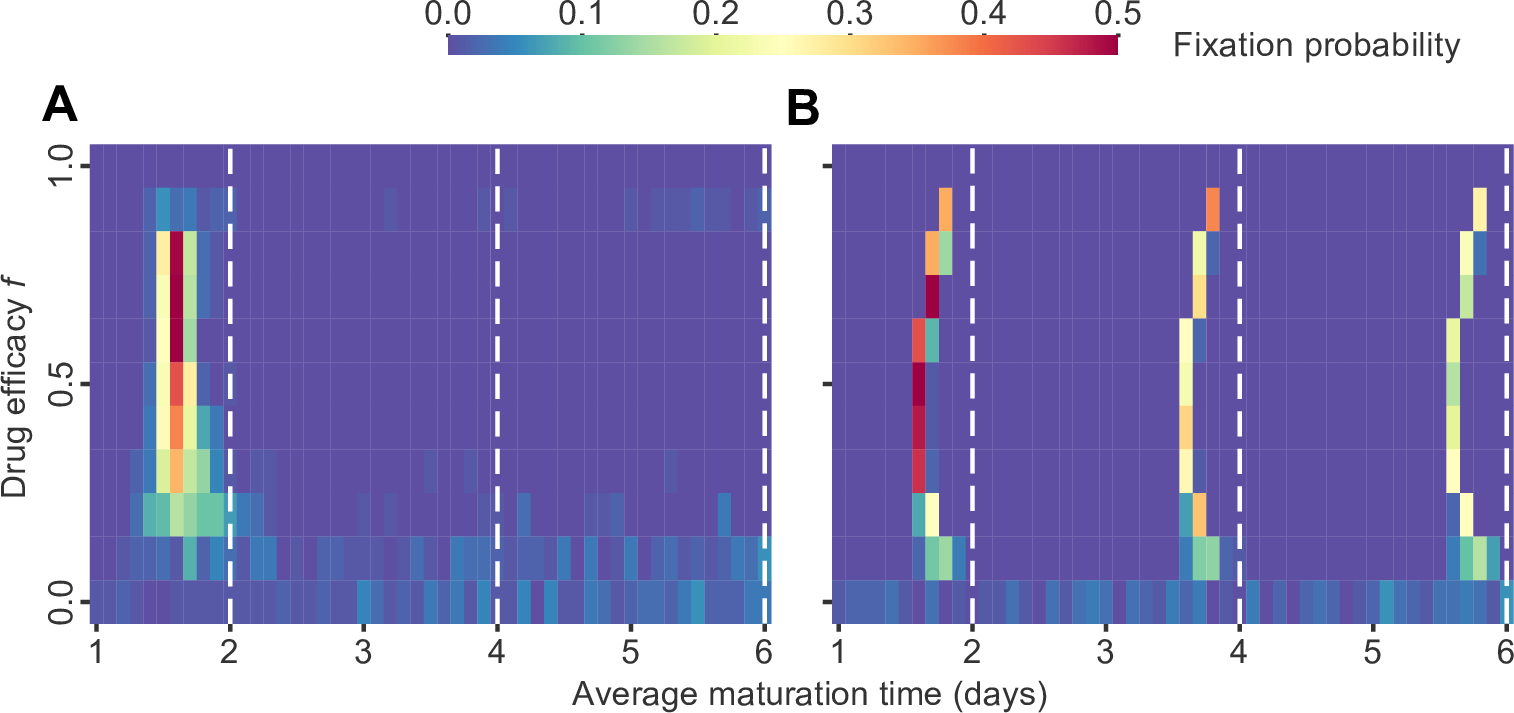

Supplement: S4 Fig — Viral dynamics were simulated under periodic antiviral therapy given by the simple on-off model with a period (T) of 2 days and varying drug efficacy (f). The fixation probability (heat map color) is measured as the fraction of simulations in which a strain was the last surviving in the population and continued on to reach a steady state. (A) Results with n = 10 maturation steps. (B) Results with fixed maturation time τ = 1/m. For all shown simulations, we assume the death rate of immature cells to be zero (dw = 0). 100 simulations were run for each drug efficacy level. Data shown for 11 different values of the drug efficacy between f = 0.0 and f = 1.0, for competitions between 51 different strains with average maturation times between 1 and 6 days. (TIF) [file pcbi.1005947.s006.tif]

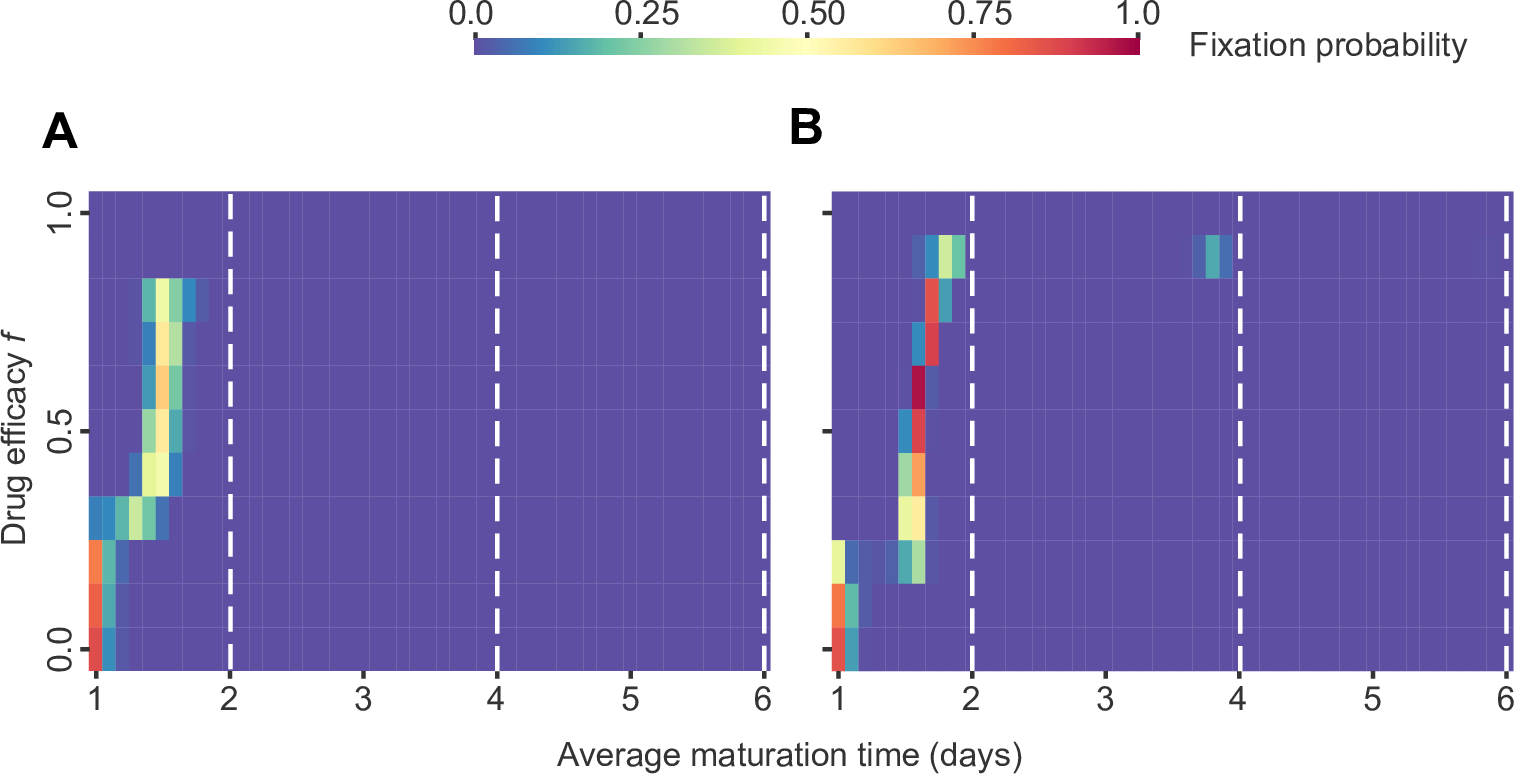

Supplement: S5 Fig — Viral dynamics were simulated under periodic antiviral therapy given by the simple on-off model with a period (T) of 2 days and varying drug efficacy (f). The fixation probability (heat map color) is measured as the fraction of simulations in which a strain was the last surviving in the population and continued on to reach a steady state. (A) Results with n = 10 maturation steps. (B) Results with fixed maturation time τ = 1/m. For all shown simulations, we assume the death rate of immature cells to be dw = 0.1. 100 simulations were run for each drug efficacy level. Data shown for 11 different values of the drug efficacy between f = 0.0 and f = 1.0, for competitions between 51 different strains with average maturation times between 1 and 6 days. (TIF) [file pcbi.1005947.s007.tif]

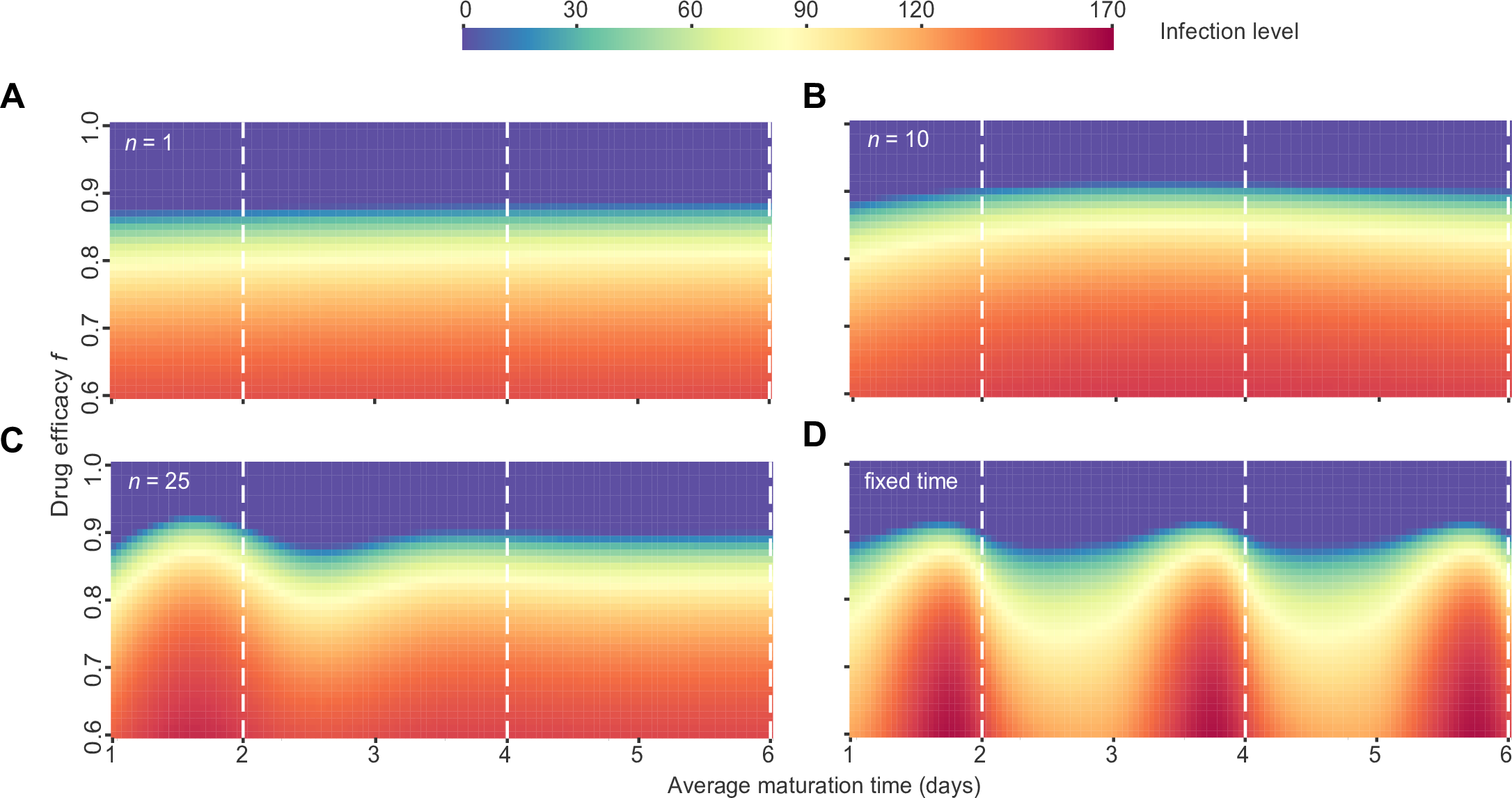

Supplement: S6 Fig — The infection level (heat map color) is measured as the concentration of mature infected cells (y) once a steady-state has been reached. Each calculation included only a single virus strain with average maturation time 1/m (maturation rate of nm for each stage). (A) Results with n = 1 maturation step. (B) Results with n = 10 maturation steps. (C) Results with n = 25 maturation steps. (D) Results with fixed maturation time τ = 1/m. The white dotted lines show where the average maturation time is equal to an integer multiple of the drug period. For all shown simulations, we assume the death rate of immature cells to be zero (dw = 0). Results shown for 41 different drug efficacies between f = 0.6 and f = 1.0, for 101 different strains with average maturation times between 1 and 6 days. (TIF) [file pcbi.1005947.s008.tif]

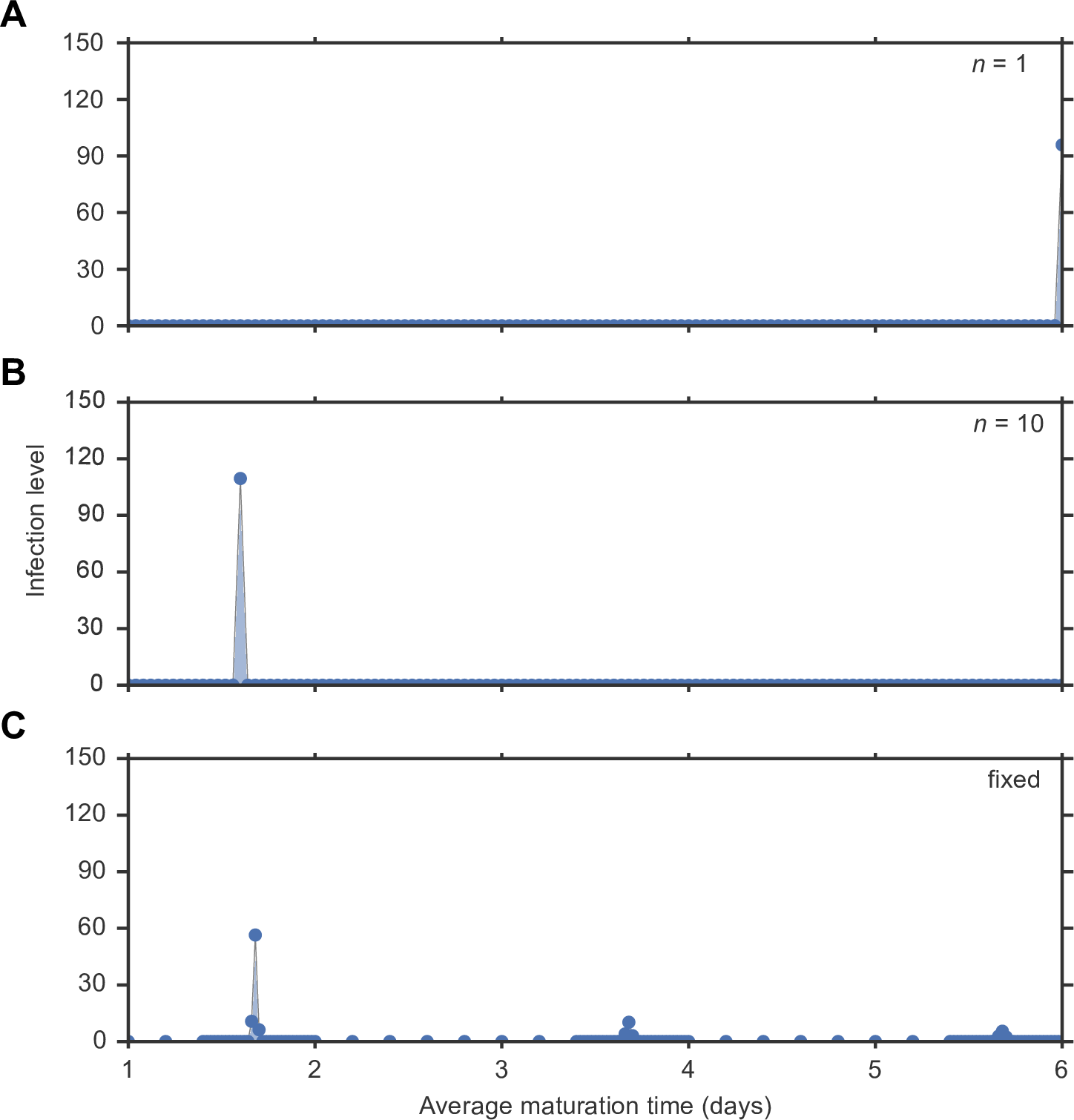

Supplement: S7 Fig — The infection level is measured as the concentration of mature infected cells y. (a) Results of deterministic model with n = 10 maturation steps. (b) Results of deterministic model with fixed maturation time τ = 1/m. The model parameters used are period T = 2 days, IC50 = 0.05, M = 1.0, Cmax = 8.0, and τ chosen such that the time-averaged drug efficacy is f = 0.85. For all shown simulations, we assume the death rate of immature cells to be zero (dw = 0). Results shown for 501, 501, and 108 different strains, for panels from A to C respectively, with average maturation times between 1 and 6 days. (TIF) [file pcbi.1005947.s009.tif]

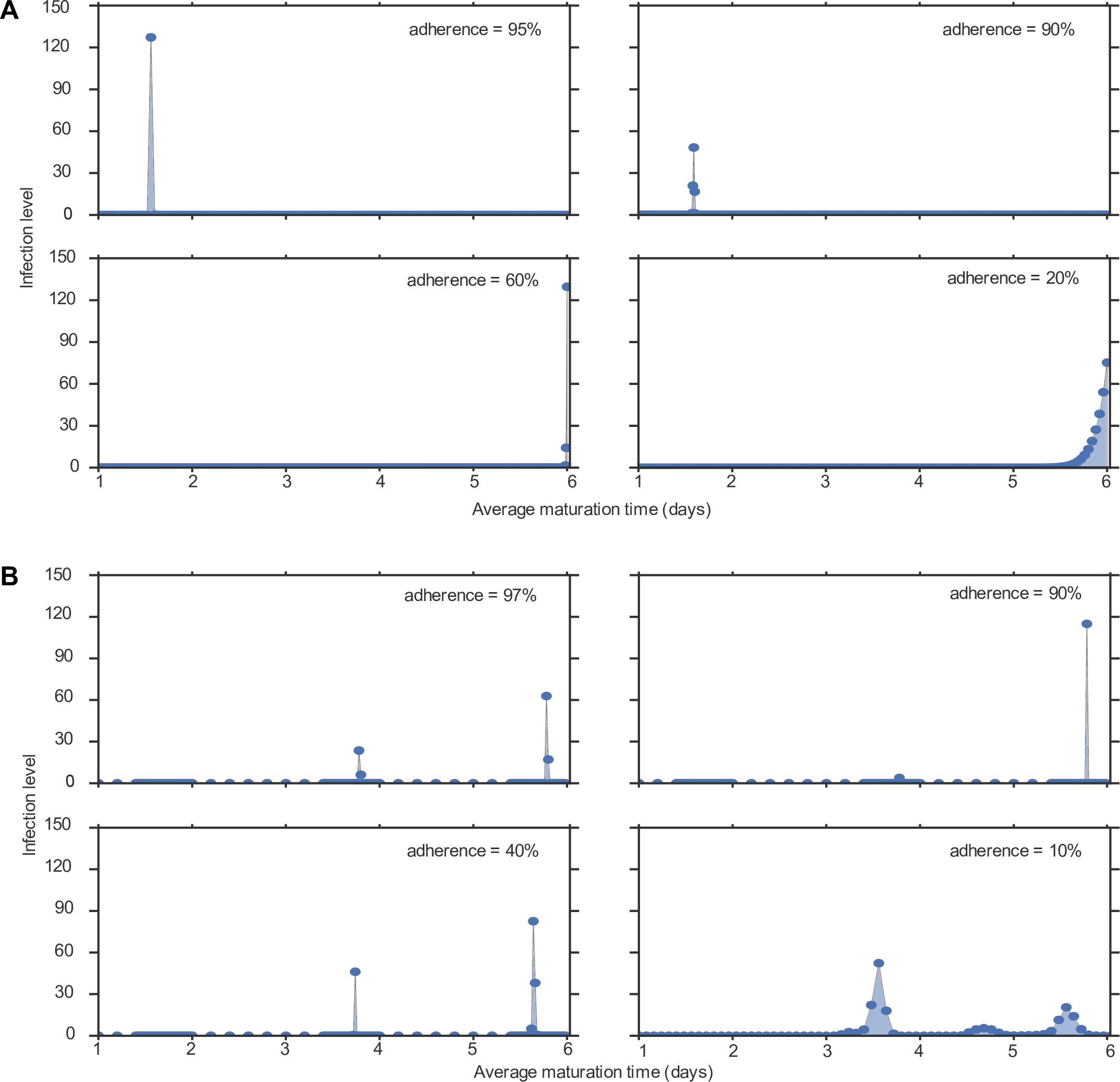

Supplement: S8 Fig — The infection level is measured as the concentration of mature infected cells y, under anti-viral therapy drug efficacy f = 0.85 and period T = 2 days. (A) Results of deterministic model with n = 10 maturation steps. (B) Results of deterministic model with fixed maturation time τ = 1/m. For all shown simulations, we assume the death rate of immature cells to be zero (dw = 0). The adherence level is the probability that any scheduled dose is taken. We assume that each dose is taken independently. Results shown for 501 and 108 different strains, for panels A and B, with average maturation times between 1 and 6 days. (TIF) [file pcbi.1005947.s010.tif]

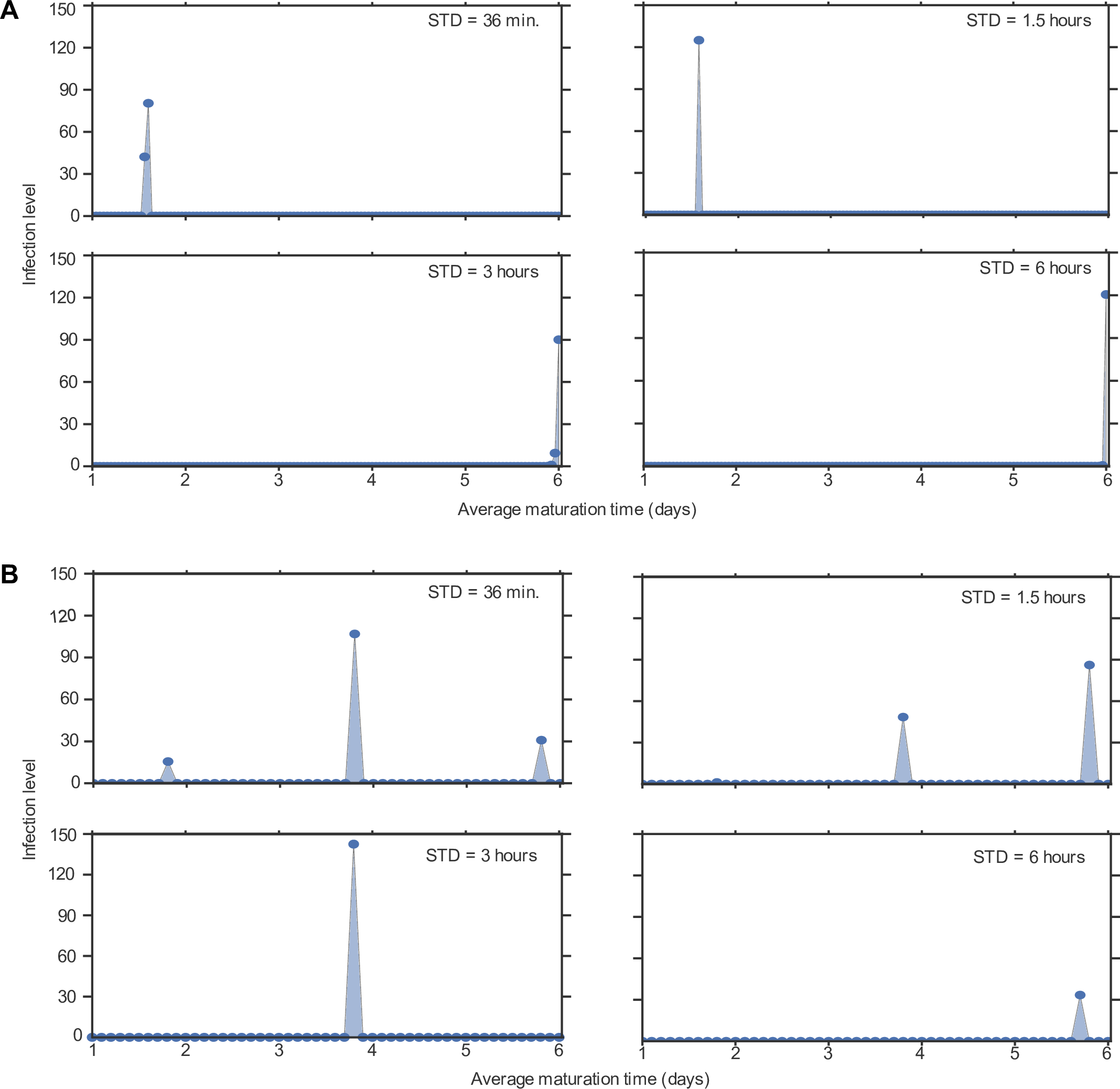

Supplement: S9 Fig — The infection level is measured as the concentration of mature infected cells y, under anti-viral therapy with drug efficacy f = 0.85 and period T = 2 days. (A) Results of deterministic model with n = 10 maturation steps. (B) Results of deterministic model with fixed maturation time τ = 1/m. For all shown simulations, we assume the death rate of immature cells to be zero (dw = 0). The drug dosage times are drawn from a normal (Gaussian) distribution with mean μ = 2 days and standard deviation σ between 36 minutes and 6 hours. Results shown for 501 and 101 different strains, for panels A and B, with average maturation times between 1 and 6 days. (TIF) [file pcbi.1005947.s011.tif]

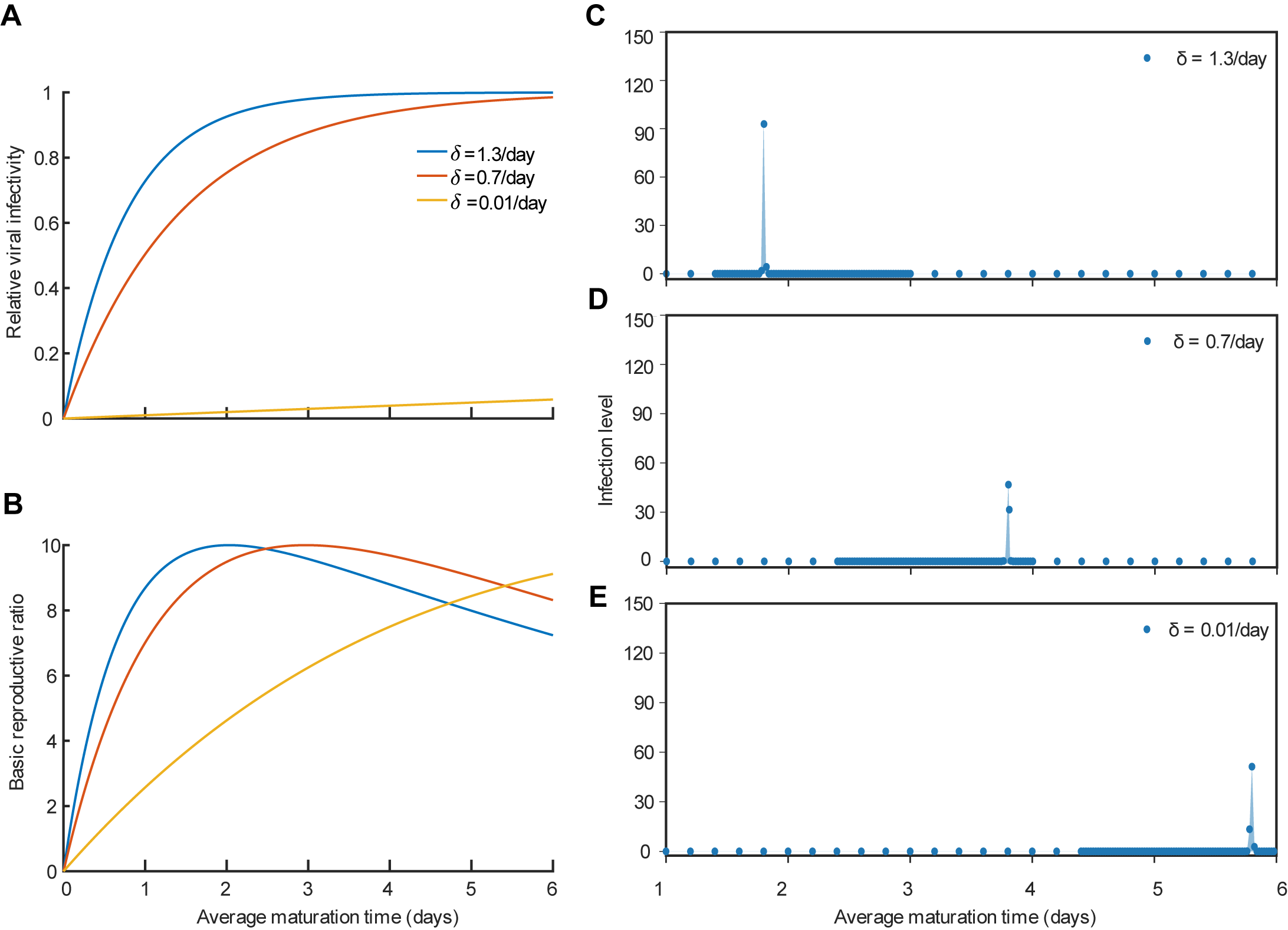

Supplement: S10 Fig — We consider a model in which there is a trade-off to the time spent in the immature phase, even in the absence of drug. The longer the time spent in this phase, the higher the eventual viral burst size and infectivity, but the more chance of dying without maturing. (A)The relationship between viral infectivity β (scaled by β0) and maturation time τ under the model of precursor production during maturation in the absence of drug. (B)The relationship between net viral fitness R0 and maturation time τ in the absence of drug. We scale the precursor production rate α so that the maximum R0 is constant across precursor decay rates. For both panels we use δ = 1.3, 0.7, or 0.01/day, dw = 0.1/day. (C-E) Equilibrium infection level for the multi-strain deterministic model under periodic drug treatment when there are trade-offs in life cycle length. The infection level (y-axis) is measured as the concentration of mature infected cells (y) once a steady-state has been reached. Each simulation included a collection of viral strains with the full range of fixed maturation times (τ) shown. Drug dynamics were given by the simple on-off model with a drug efficacy of 85% (f = 0.85). (C) Precursor decay rate δ = 1.3/day. (D) Precursor decay rate δ = 0.7/day. (E) Precursor decay rate δ = 0.01/day. For all shown simulations, we assume the death rate of immature cells to be dw = 0.1. (TIF) [file pcbi.1005947.s012.tif]

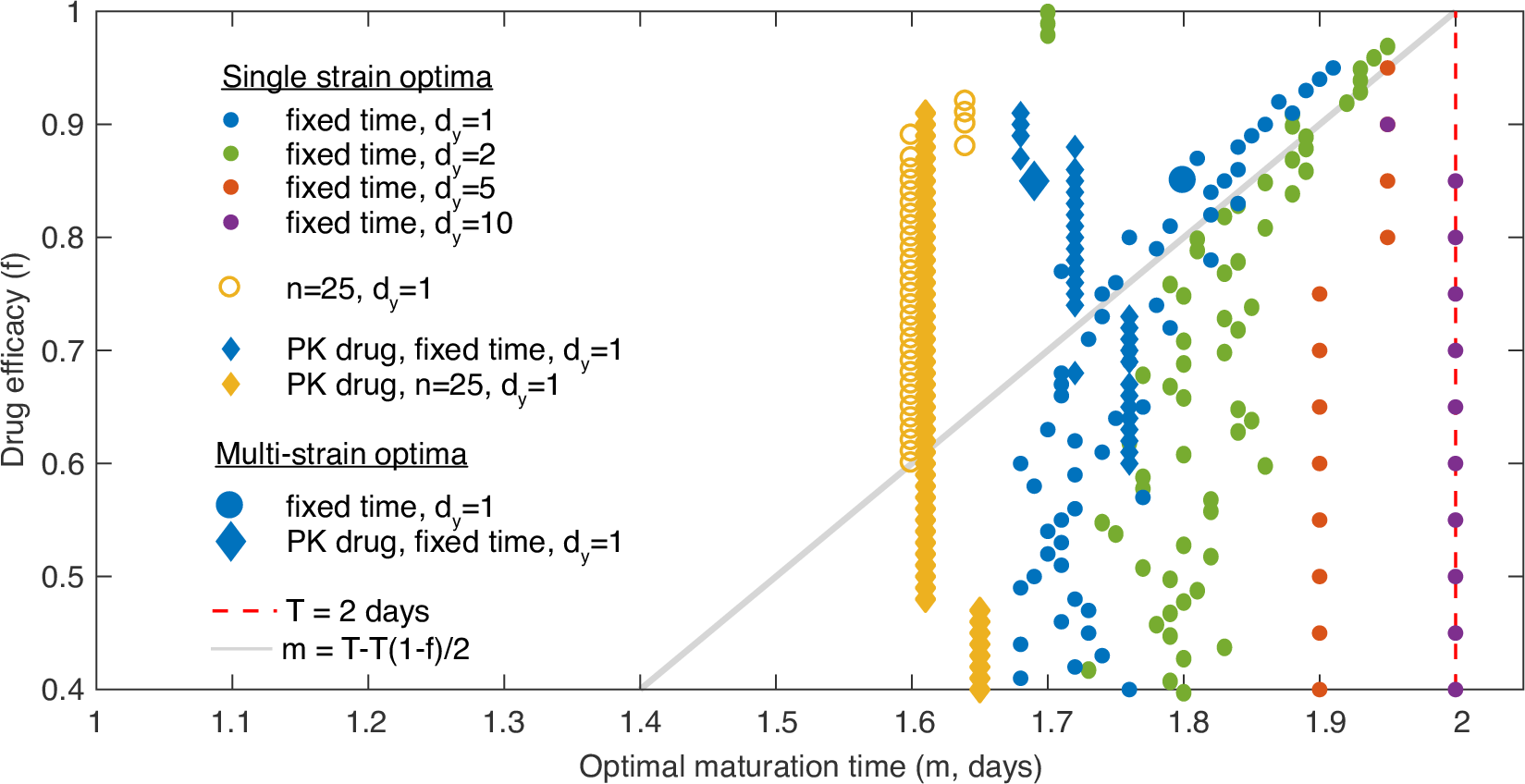

Supplement: S11 Fig — The optimal strain is defined as the one with the highest time-average equilibrium level of mature infected cells (y), either when simulated alone (single strain models) or in competition with other strains (multi-strain model). In all cases we only looked at strains with maturation times less than or equal to the drug period T = 2 days. When dy was altered from 1, β was increased by the same amount to keep R0 in the absence of drug the same. For all shown simulations, we assume the death rate of immature cells to be zero (dw = 0). The grey line describes the curve m = T − T(1 − f)/2. The total viral generation time is the sum of the maturation time (x-axis) and the average time between a cell maturing and a secondary infection occurring. When the average lifespan of a mature infected cell (1/dy) exceeds the time the drug is off (T(1 − f)) in the simple on-off model, then the average time until secondary infection is T(1 − f)/2), so the optimal maturation time is expected to be lower than the drug period by this amount. This prediction only works when dy is small, maturation time is tightly controlled, drug displays simple on-off kinetics, and drug efficacy is relatively high. (TIF) [file pcbi.1005947.s013.tif]

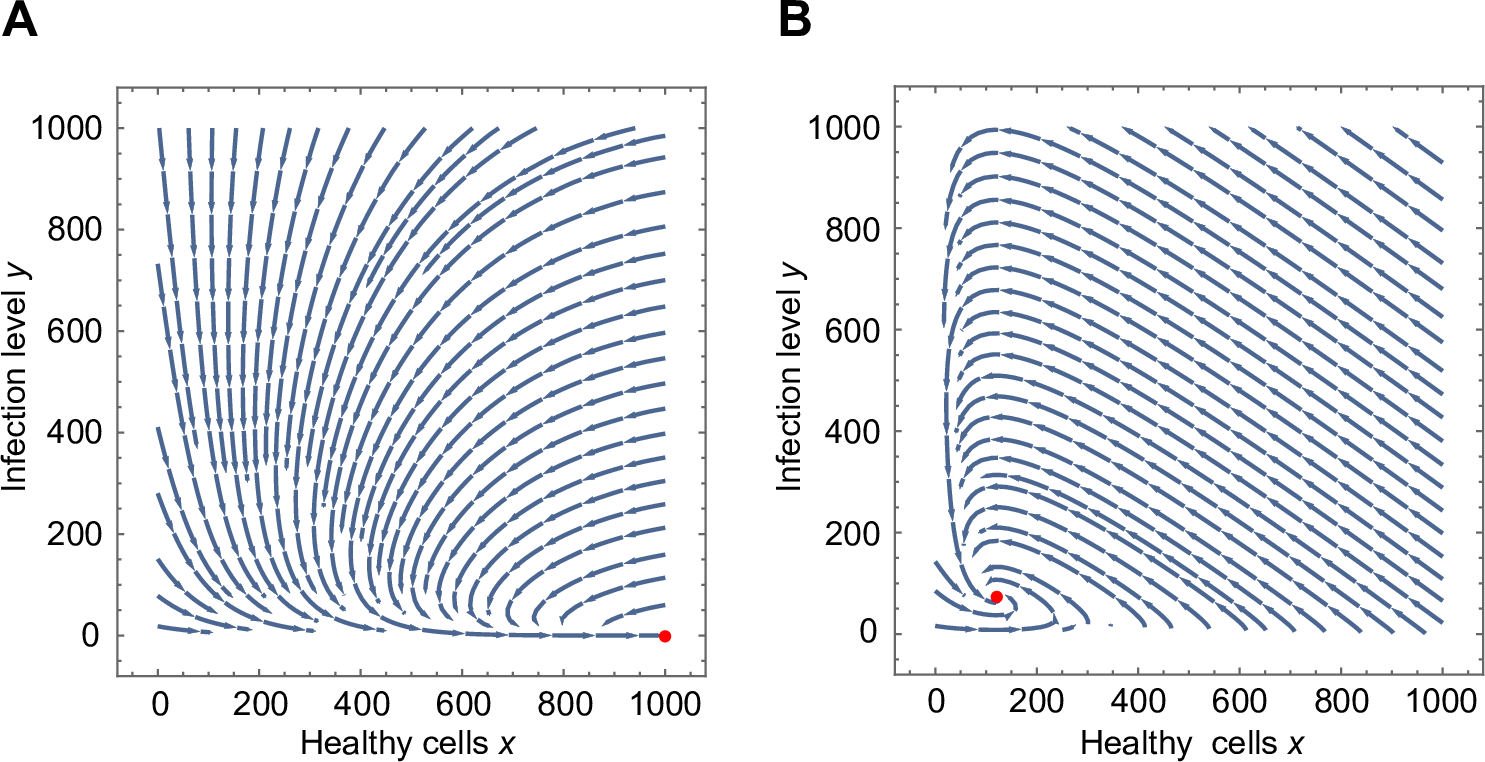

Supplement: S12 Fig — (A) Stability of no-infection steady state when R0 < 1. (B) Stability of infection steady state when R0 > 1. The corresponding equations are shown in Eq (1) and Eq. S.1. The red point represents the equilibrium point for the system. For all shown results, we set the death rate of immature cells to dw = 0.1, and we assume that the concentration of healthy cells x and the concentration of immature infected cells w are close to steady state. (TIF) [file pcbi.1005947.s014.tif]

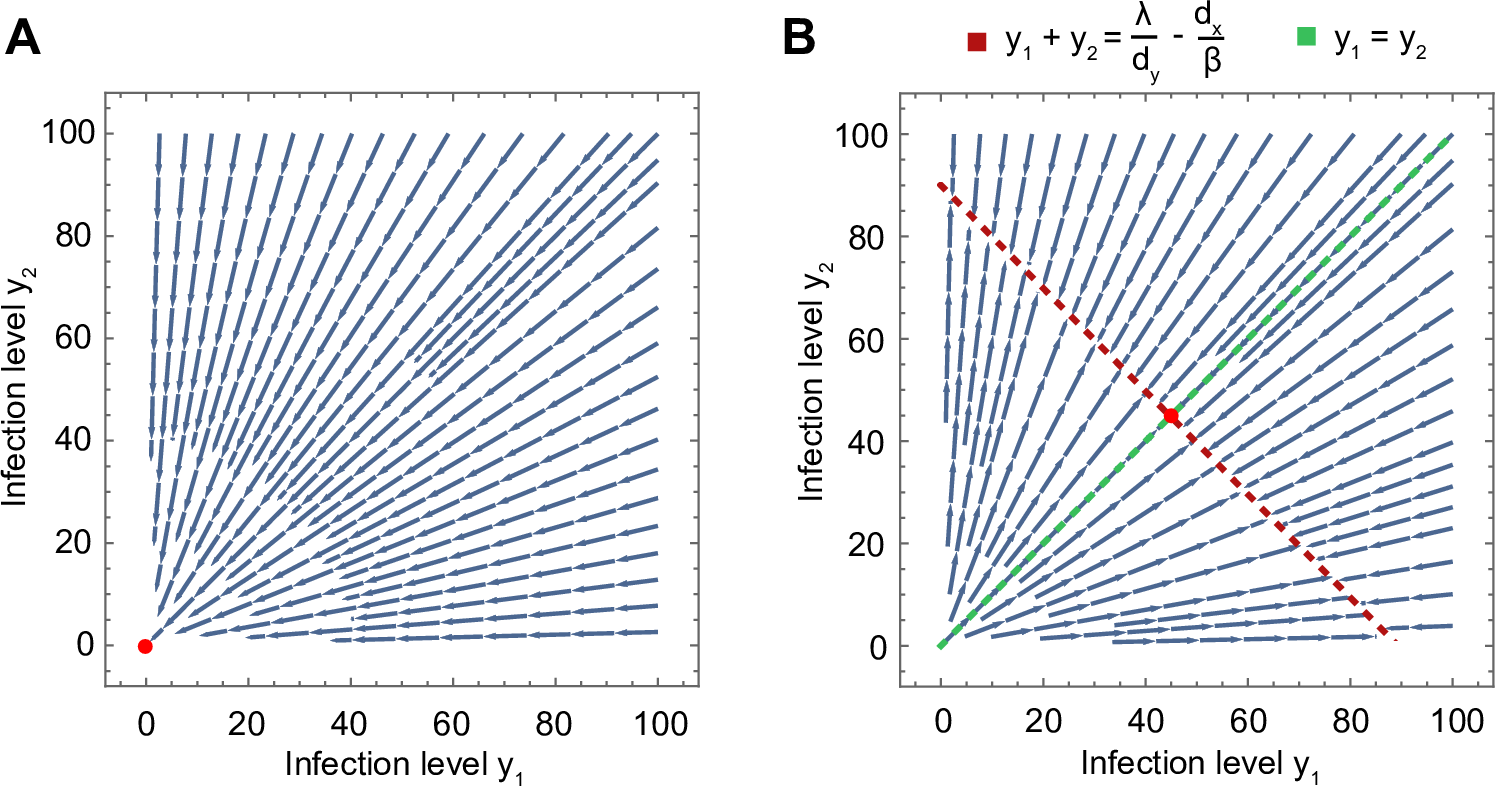

Supplement: S13 Fig — (A) Stability of no-infection steady state when R0 < 1 for both viral strains. (B) Stability of infection steady state when R0 > 1 for both viral strains. The corresponding equations are shown in Eq. S.3. The red point represents the equilibrium point for the system. For all shown results, we assume the death rate of immature cells to be zero (dw = 0), and that the concentration of healthy cells x and immature infected cells w1,2 are close to steady state. (TIF) [file pcbi.1005947.s015.tif]

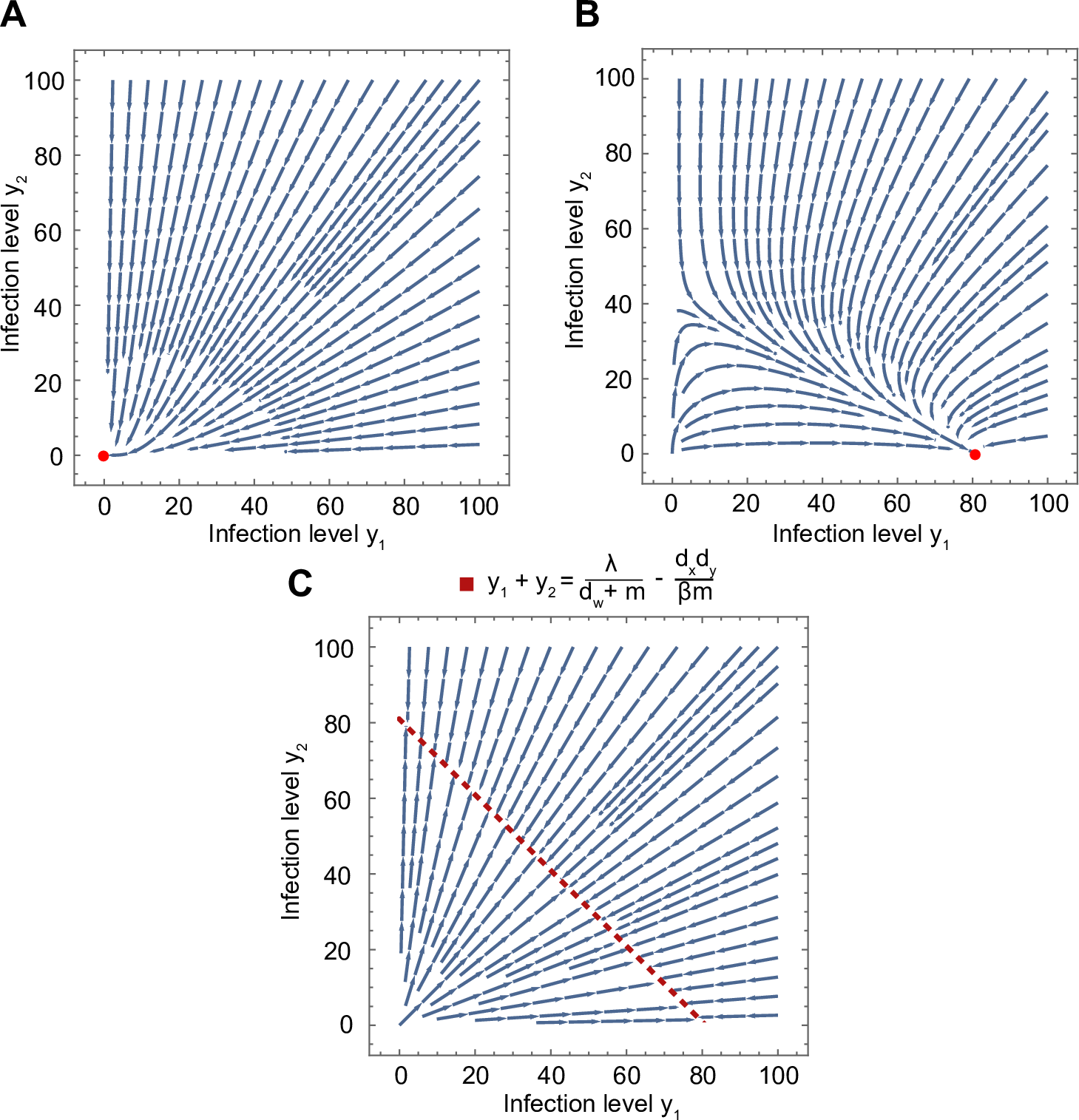

Supplement: S14 Fig — (A) Stability of no-infection steady state when R0 < 1 for both viral strains and m1 > m2. (B) Stability of infection steady state when the basic reproductive ratio R0 > 1 for both viral strains and m1 > m2. (C) Stability of infection steady state when the basic reproductive ratio R0 > 1 for both viral strains and m1 = m2. The corresponding equations are shown in Eq. S.3. The red point represents the equilibrium point for the system. For all shown results, we set the death rate of immature cells to dw = 0.1, and we assume that the concentration of healthy cells x and immature infected cells w1,2 are close to steady state. (TIF) [file pcbi.1005947.s016.tif]

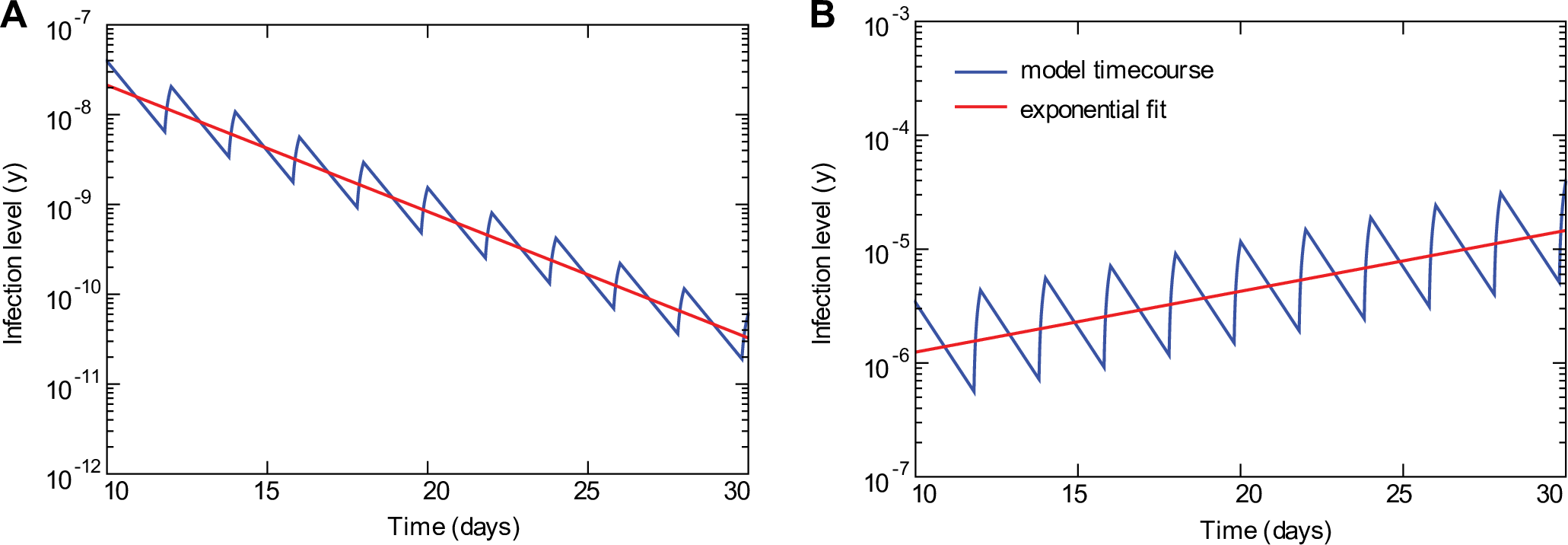

Supplement: S15 Fig — Infection dynamics with the fixed-delay model (Eq (3)) were numerically integrated starting from a small amount of infection introduced to the uninfected equilibrium. The level of mature infected cells is shown (blue line). To estimate r˜0 from these dynamics, we took points sampled at intervals of T and fit to an exponential curve (red line). We compared this to the value of r˜0 predicted from our analytic work (Eq. S.99). a) Maturation time of τ = 0.4: infection decreases with drug. The observed value of r˜0 (−0.324) matches excellently with the predicted value (−0.324). b) Maturation time of τ = 1.6: infection grows despite drug. The observed value of r˜0 (0.123) matches excellently with the predicted value (0.123). We used parameters from Table 1 along with dw = 0.1 and f = 0.9. Initial values were x(0) = 103 and y(0) = 10−6. Fits were conducted using points between times of 10 and 30 days using the method of least squares. (TIF) [file pcbi.1005947.s017.tif]
